# Structural Consensus among Antibodies Defines the Antigen Binding Site

**DOI:** 10.1371/journal.pcbi.1002388

**Published:** 2012-02-23

**Authors:** Vered Kunik, Bjoern Peters, Yanay Ofran

**Affiliations:** 1 The Goodman faculty of life sciences, Nanotechnology building, Bar Ilan University, Ramat Gan, Israel; 2 Division of Vaccine Discovery, La Jolla Institute for Allergy and Immunology, La Jolla, California, United States of America; University of Notre Dame, United States of America

## Abstract

The Complementarity Determining Regions (CDRs) of antibodies are assumed to account for the antigen recognition and binding and thus to contain also the antigen binding site. CDRs are typically discerned by searching for regions that are most different, in sequence or in structure, between different antibodies. Here, we show that ∼20% of the antibody residues that actually bind the antigen fall outside the CDRs. However, virtually all antigen binding residues lie in regions of structural consensus across antibodies. Furthermore, we show that these regions of structural consensus which cover the antigen binding site are identifiable from the sequence of the antibody. Analyzing the predicted contribution of antigen binding residues to the stability of the antibody-antigen complex, we show that residues that fall outside of the traditionally defined CDRs are at least as important to antigen binding as residues within the CDRs, and in some cases, they are even more important energetically. Furthermore, antigen binding residues that fall outside of the structural consensus regions but within traditionally defined CDRs show a marginal energetic contribution to antigen binding. These findings allow for systematic and comprehensive identification of antigen binding sites, which can improve the understanding of antigenic interactions and may be useful in antibody engineering and B-cell epitope identification.

## Author Summary

Antibodies are a primary adaptive defence mechanism against infection, and function by recognizing and binding to non-self antigens. While most of the sequence of all antibodies of a given individual is identical, relatively small variations turn each antibody into a specific binder of one antigen. It is widely assumed that antigen binding sites correspond to the so called Complementarity Determining Regions (CDRs) of the antibody, which are defined as the elements that are most different between antibodies. We analysed all known antibody-antigen complexes and found that about 20% of the residues that actually bind the antigen fall outside the CDRs. However, we also found that virtually all antigen binding residues fall within regions of structural consensus between antibodies. Moreover, we demonstrate that antigen binding residues that reside within these structural consensus regions but outside of the traditionally-defined CDRs make significant energetic contribution to antigen binding. Furthermore, we show that these regions are organized along the sequence of the antibody chains and are identifiable from the sequence of the antibody.

## Introduction

Antibody-Antigen (Ab-Ag) interactions are based on non-covalent binding between the antibody (Ab) and the antigen (Ag). Correct identification of the residues that mediate Ag recognition and binding would improve our understanding of antigenic interactions and may permit the modification and manipulation of Abs. For example, introducing mutations into the V-genes has been suggested as a way to improve Ab affinity [1]–[3]. However, mutations in the framework regions (FRs) rather than in the Ag binding residues themselves are more likely to evoke an undesired immune response [4]. Knowing which residues bind the Ag can help direct such mutations and be beneficial to Ab engineering [5]–[7]. It has been shown that Ag binding residues are primarily located in the so called complementarity determining regions (CDRs) [7]–[9]. Thus, the attempt to identify CDRs, and particularly the attempt to define their boundaries, has become the focus of extensive research over the last few decades [7], [8], [10]. Kabat and co-workers [9], [11] attempted to systematically identify CDRs in newly sequenced Abs. Their approach was based on the assumption that CDRs include the most variable positions in Abs and therefore could be identified by aligning the fairly limited number of Abs available then. Based on this alignment they introduced a numbering scheme for the residues in the hypervariable regions and determined which positions mark the beginning and the end of each CDR. The Kabat numbering scheme was developed when no structural information was available. Chothia et al. [12], [13] analyzed a small number of Ab structures and determined the relationship between the sequences of the Abs and the structures of their CDRs. The boundaries of the FRs and the CDRs were determined and the latter have been shown to adopt a restricted set of conformations based on the presence of certain residues at key positions in the CDRs and the flanking FRs. This analysis suggested that the sites of insertions and deletions in CDRs L1 and H1 are different than those suggested by Kabat. Thus, the Chothia numbering scheme is almost identical to the Kabat scheme, but based on structural considerations, places the insertions in CDRs L1 and H1 at different positions. As more experimental data became available, the analysis was performed anew, re-defining the boundaries of the CDRs. These definitions of CDRs are mostly based on manual analysis and may require adjustments as the structure of more Abs become available. Abhinandan et al. [14] aligned Ab sequences in the context of structure and found that approximately 10% of the sequences in the manually annotated Kabat database have erroneous numbering. A more recent attempt to define CDRs is that of the IMGT database [15] which curates nucleotide sequence information for immunoglobulins (IG), T-cell receptors (TcR) and Major Histocompatibility Complex (MHC) molecules. It proposes a uniform numbering system for IG and TcR sequences, based on aligning more than 5000 IG and TcR variable region sequences, taking into account and combining the Kabat definition of FRs and CDRs [16], structural data [17] and Chothia's characterization of the hypervariable loops [12]. Their numbering scheme does not differentiate between the various immunoglobulins (i.e., IG or TcR), the chain type (i.e., heavy or light) or the species.

A drawback of these numbering schemes is that CDRs length variability is accommodated with either annotation of insertion (Kabat and Chothia) or by providing excess numbers (IMGT). Abs with unusually long insertions may be hard to annotate this way, and therefore their CDRs may not be identified correctly. Honegger and Pluckthun [18] suggested a structurally improved version of the IMGT scheme. Instead of introducing unidirectional insertions and deletions as in the IMGT and Chothia schemes, they were placed symmetrically around a key position. MacCallum et al. [8] have proposed focusing on the specific notion of Ag binding residues rather than the more vague concept of CDRs. They suggested that these residues could be identified based on structural analysis of the binding patterns of canonical loops. Other studies have dubbed those Ag binding residues Specificity Determining Regions (SDRs) [5], [7]. Here, we analyze Ag-Ab complexes and show that virtually all Ag binding residues fall within regions of structural consensus. We refer to these regions as Ag Binding Regions (ABRs). We show that these regions can be identified from the Ab sequence as well. We used “Paratome”, an implementation of a structural approach for the identification of structural consensus in Abs [19]. While residues identified by Paratome cover virtually all the Ag binding sites, the CDRs (as identified by the commonly used CDR identification tools) miss significant portions of them. We refer to the Ag binding residues which are identified by Paratome but are not identified by any of the common CDR identification methods, as Paratome-unique residues. Similarly, Ag binding residues that are identified by any of the common CDR identification methods but are not identified by Paratome are referred to as CDRs-unique residues. We show that Paratome-unique residues make crucial energetic contribution to Ab-Ag interactions, while CDRs-unique residues have a rather minor contribution. These results allow for better identification of Ag binding sites and thus for better identification of B-cell epitopes. They may also help improve vaccine and Ab design.

## Results

### Structural consensus defines ABRs

**Figure 1:**
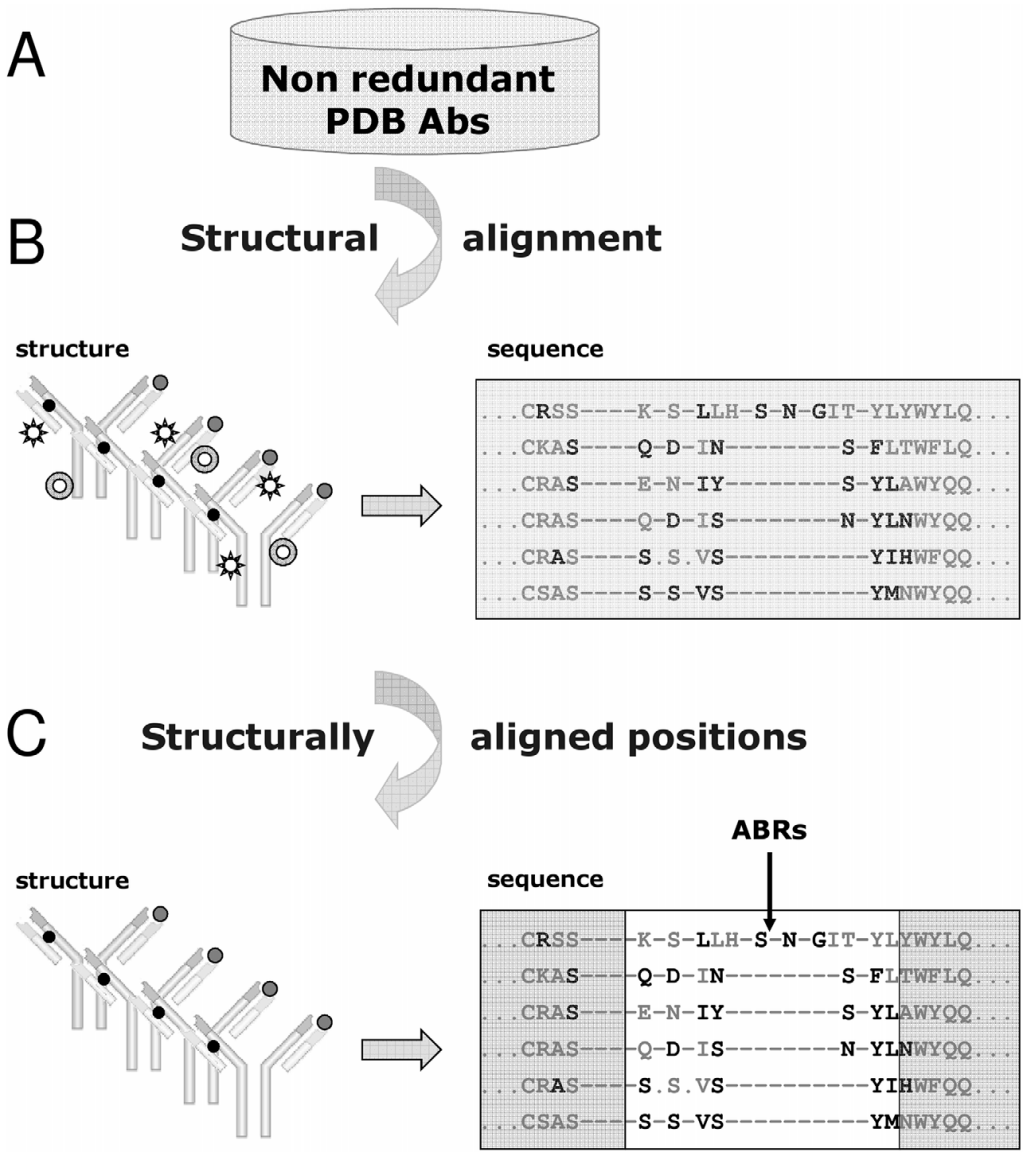
Structure-based identification of ABRs. (A) Using the non-redundant set of all Ab-Ag complexes in the PDB, (B) we created a multiple structure alignment of the Abs. Residues that are in contact with the Ag were identified by searching for structurally aligned positions that systematically create contacts with the Ag (black and grey solid circles) and disregarded positions that contact the Ag only sporadically (open shapes). (C) The contacting positions were mapped to the sequence representation of the multiple structure alignment (bold letters). The stretches of amino acids in which at least 10% of the Abs are in contact with the Ag were defined as ABRs (white rectangle).

The outline of our structure-based ABRs identification method is delineated in Figure 1. Briefly, the algorithm structurally aligns all known Abs and marks the residues that contact the Ag in each of them. We have shown [19], [20] that in this multiple structure alignment there is a consensus among Abs that some structurally aligned positions contact the Ag. These positions form six sequence stretches along the Ab sequence that roughly correspond to the six CDRs. Beyond the edges of these stretches there were no structurally aligned positions in which more than 10% of the Abs contact the Ag. Thus, we defined the boundaries of the ABRs based on these stretches and marked the ABRs in all the Abs in our dataset.

### Paratome: Automatic sequence based ABRs identification

**Figure 2:**
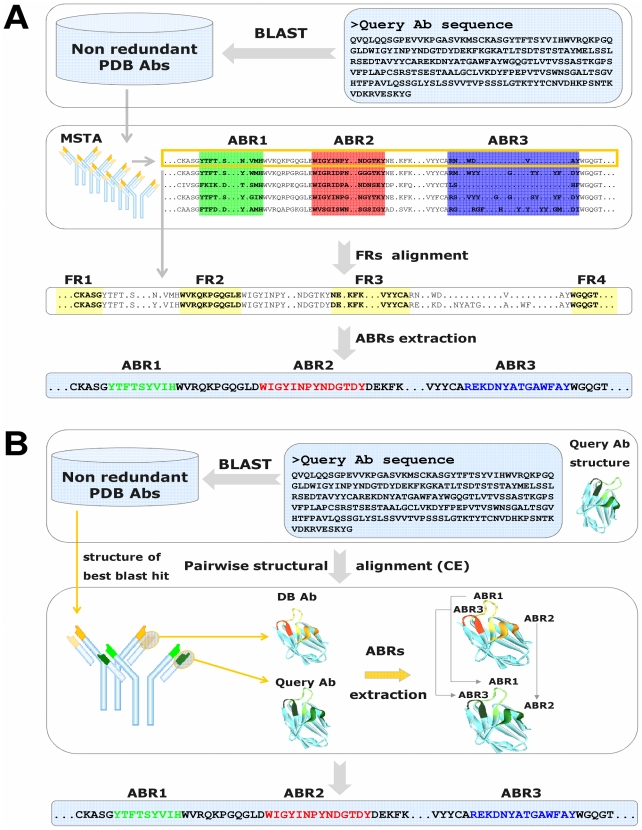
Automated ABRs Identification (**A**) **Sequence based ABRs identification.** A BLAST search is performed using the query Ab sequence versus the dataset of non-redundant PDB Abs. Using the best hit from the BLAST search, the query and annotated Abs FRs are aligned and hence the query sequence ABRs are inferred based on the location of the annotated sequence ABRs in the MSTA. (**B**) **Structure based ABRs identification.** A BLAST search is performed using the sequence of the query Ab versus our dataset of Abs. Using the best hit from the BLAST search, the query and annotated Abs are structurally aligned. The ABRs of the query Ab are inferred based on the location of the annotated Ab ABRs in the MSTA.

Figure 2 depicts the automated ABRs identification tool we developed. Given a query sequence (Figure 2A) a BLAST search is performed against all Abs in the dataset described above. The best hit (i.e., lowest E-value) is used to infer the positions of the ABRs in the query sequence, based on its alignment to the annotated Ab from the dataset. When the query Ab has a known 3-D structure, it can be used to identify the ABRs as described in Figure 2B (see Methods).

### Content statistics

**Figure 3:**
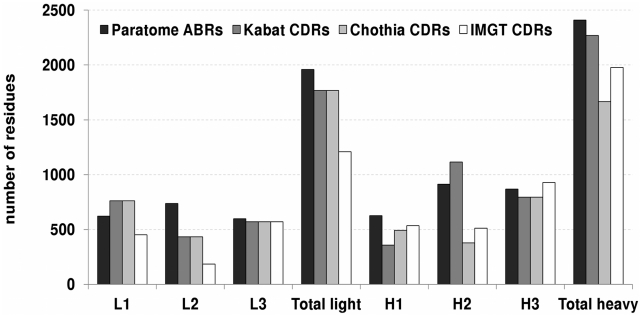
Total number of residues identified by each method for all Ab-Ag complexes in the test set. L1–L3 are ABR/CDR1-3 of the light chain. H1–H3 are ABR/CDR1-3 of the heavy chain. Total light and heavy are the sum of all identified residues in the light and heavy chains respectively.

Figure 3 summarizes the number of residues identified by each method on the test set. In all regions except L1 and H2, Paratome identified a slightly larger number of residues than any other method. The largest differences were recorded in L2 and H2. In L2, Paratome had 50% more residues identified than Kabat and Chothia and four times the number of residues identified by IMGT. For H2, Kabat and Paratome identified twice the number of residues suggested by Chothia and IMGT.

### Structural consensus regions contain virtually all Ag binding residues

**Figure 4:**
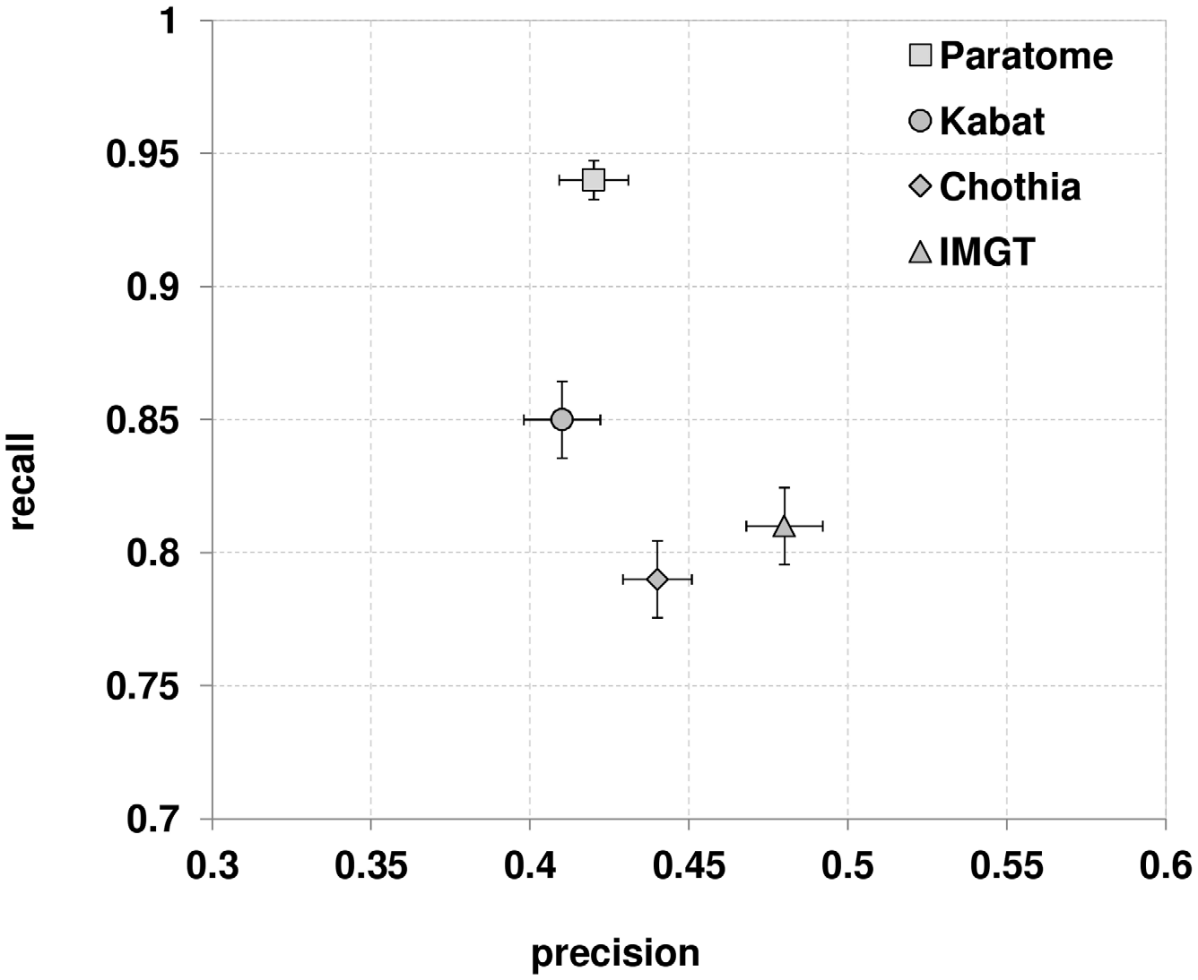
Recall and precision of Ag binding sites identification. Average precision and recall were calculated for the Abs in the test set for Paratome, Kabat, Chothia and IMGT methods. Error bars represent standard error of the mean.

For each Ab in our test dataset we recorded the average recall of the residues that actually bind the Ag by each method. Given the typical trade-off between recall and precision in which the increase of one is at the cost of decreasing the other, we measured the average precision of each method. The results are presented in Figure 4. The ABRs identified by Paratome included 94% of Ag binding residues, followed by Kabat (85%), IMGT (81%) and Chothia (79%) CDRs. Precision rates ranged between 48% (IMGT) and 41% (Kabat), with Chothia (44%) and Paratome (42%) in between.

### ABRs-specific residues cover 10–17% of the Ag binding sites

Table 1 compares the consensus sets and the method specific sets of residues. The Paratome-Kabat consensus set is the largest (3476 residues), covering 83.54% of the Ag binding sites. Paratome-Chothia consensus set covered 77.08% of the Ag binding sites (3203 residues), and Paratome-IMGT consensus set covered 79.47% of the Ag binding sites (3077 residues). In all consensus sets, approximately 50% of the residues are Ag binding residues. ΔParatome contains a substantially larger percentage of Ag binding residues than ΔKabat, ΔChothia and ΔIMGT (20.8%, 26.23% and 20.6% respectively, compared with 5.03%, 4.88% and 6.88% respectively).

**Table 1:** Ag binding sites coverage by consensus and method-specific residues.

Residues set	# of residues	# of residues in contact	binding sites coverage
consensus Paratome - Kabat	3476	1664	83.54%
ΔParatome	1018	212	10.77%
ΔKabat	695	35	1.78%
consensus Paratome - Chothia	3202	1517	77.08%
ΔParatome	1292	339	17.23%
ΔChothia	348	17	0.86%
consensus Paratome - IMGT	3077	1564	79.47%
ΔParatome	1417	292	14.84%
ΔIMGT	218	15	0.76%

For each set, we recorded the total number of residues, the number of Ag contacting residues and the percentage of Ag binding sites coverage. In all of the comparisons, Paratome-specific residues covered a significantly larger portion of the Ag binding sites.

Moreover, ΔParatome residues cover a significantly larger portion of the Ag binding sites. ΔParatome residues covered 10.77% of the Ag binding sites while ΔKabat covered merely 1.78% of the Ag binding sites. The coverage of ΔParatome (14.84%) was 20 times larger than that of ΔIMGT (0.76%). When compared to Chothia, the coverage of ΔParatome (17.23%) was, again, more than an order of magnitude greater than that of ΔChothia's (0.86%). In each comparison, Paratome-specific residues covered a significantly larger portion of the Ag binding sites than the alternative method-specific residues. Thus, indicating that structural consensus regions capture more of the Ag binding portion of Abs.

### Differences in ratios of Ag binding residues

**Figure 5:**
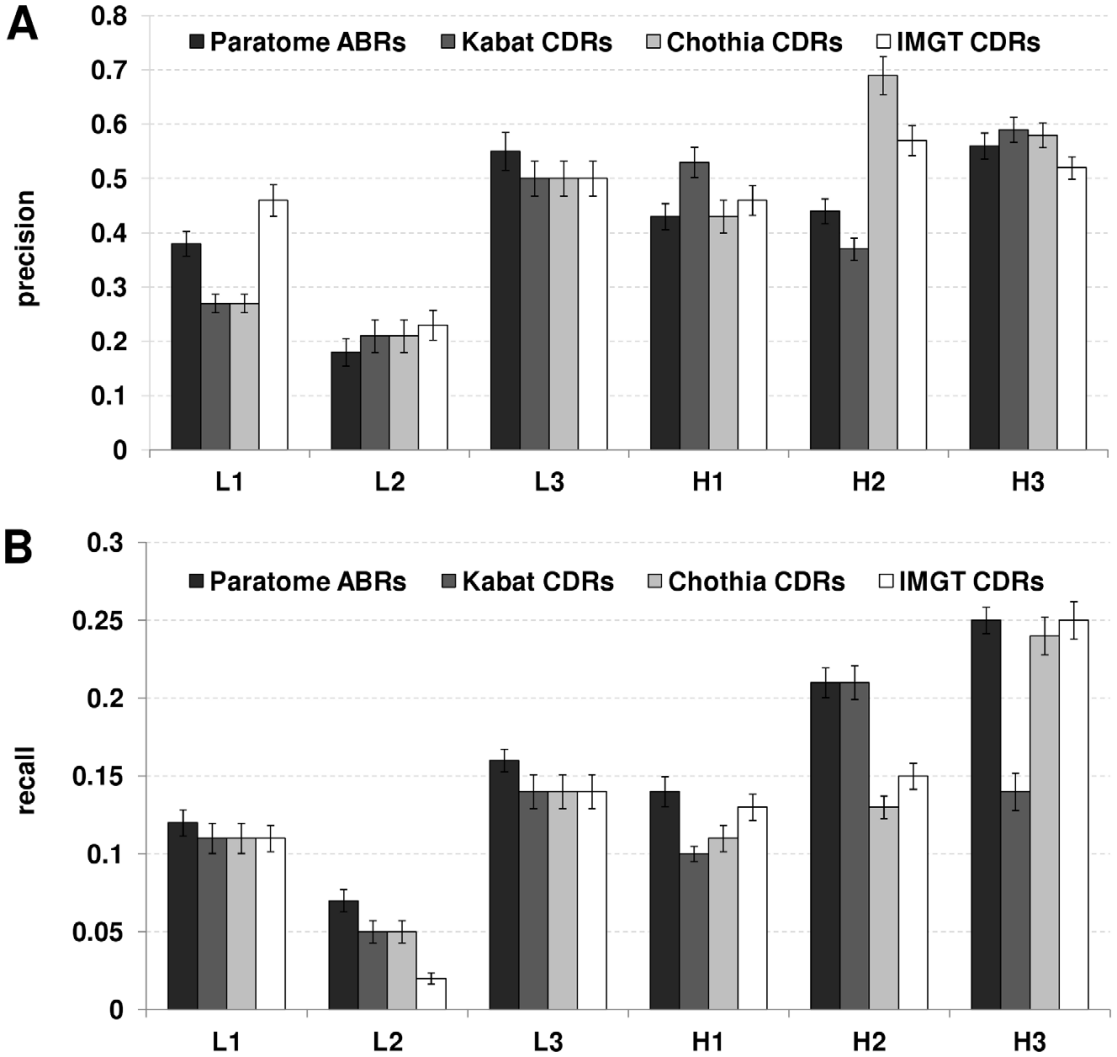
Average Ag binding sites recall and precision of light and heavy chains for all ABRs/CDRs. (A) Average Ag binding sites precision (B) Average Ag binding sites recall. Error bars represent standard error of the mean.

Figure 5A shows the average precision for each ABR/CDR on the light and heavy chains as defined by each of the methods. L2 has the lowest precision in all methods. For L3, all the methods have a similar precision, with a slightly higher rate for Paratome (0.55). IMGT has the highest precision for L1 (0.46), followed by Paratome (0.38) and Chothia and Kabat has the lowest precision (0.27). The largest difference between the methods is in H2 where Chothia has the highest precision (0.69), followed by IMGT (0.57), then Paratome (0.43) and Kabat (0.37).

Figure 5B summarizes the average recall of each method for each of the six regions. For all methods, L2 has the lowest recall (2–7%). This is expected considering L2 has the lowest precision (see Figure 5A). For L1, all methods show similar recall (11–12%). The same holds for H3, which covers the largest fraction of the Ag binding sites (24–25%). H2 shows the highest diversity; For Paratome and Kabat it covers 21% of the Ag binding sites while for Chothia and IMGT recall ranged between 13–15%, respectively. In all cases, Paratome shows the highest recall. Note that while the overall recall ranges between 0.7–1 (see Figure 4), the recall of each of the six regions ranges between 0–0.3. This is due to the fact that the total recall is the accumulation of the recall obtained by each of the six regions.

### Paratome-unique residues are important for Ag binding

**Figure 6:**
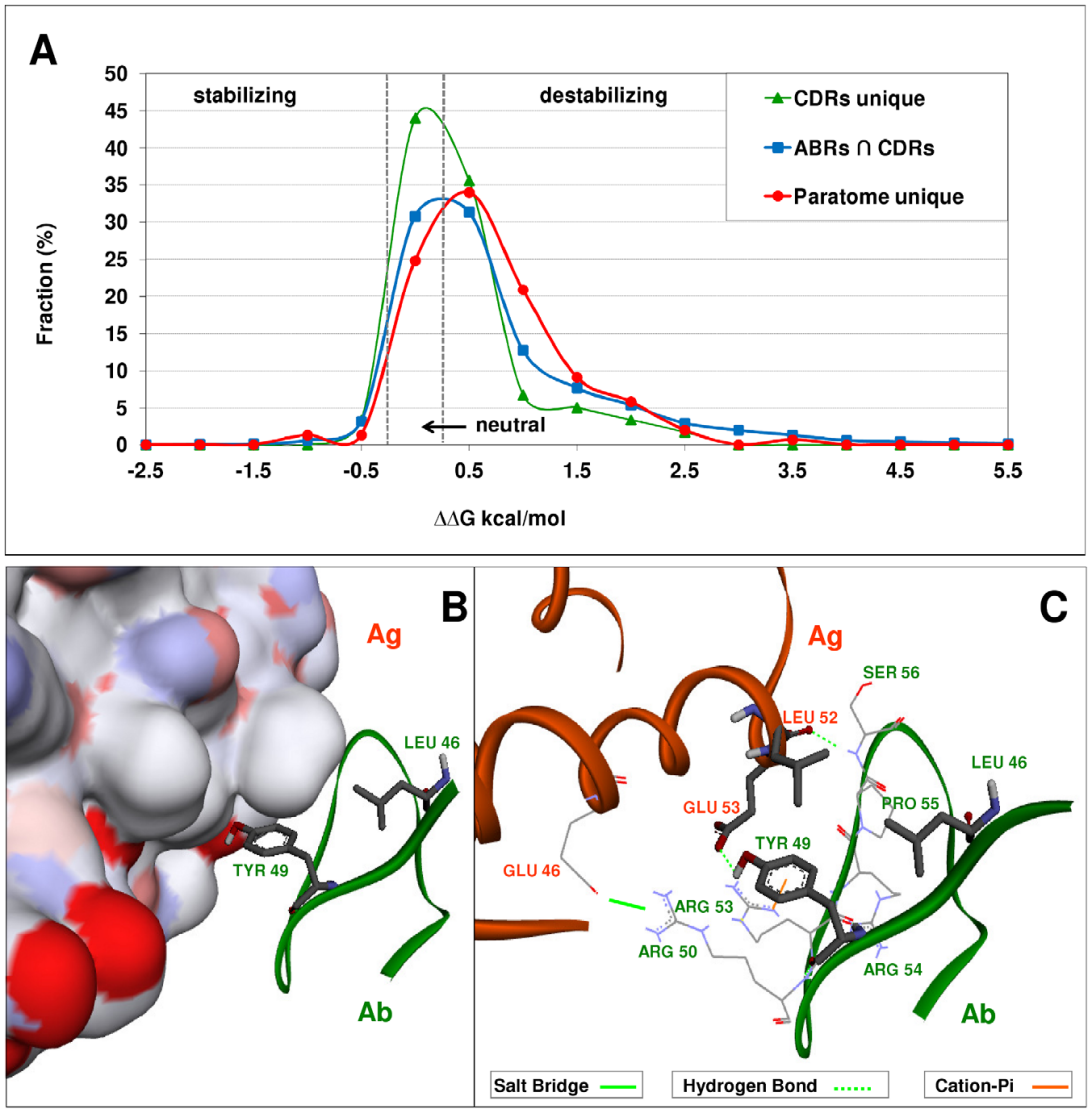
Contribution of Paratome-unique and CDR-unique residues to the binding energy in Ab-Ag complexes. (A) The distributions of ΔΔG values of an in-silico alanine scan analysis of Paratome-unique, CDRs-unique and CDR Ag binding residues. ΔΔG values ranging between −0.25 and 0.25 were defined as neutral. ΔΔG values<−0.25 were defined as stabilizing. ΔΔG values >0.25 were defined as destabilizing. It is clear from the distributions that typically, a Paratome-unique residue is at least as energetically important as a residue in the CDRs, while a CDR unique residue is less energetically important relative to residues within the CDRs that are identified by Paratome. (B)+(C) A detailed analysis of anti-IL-15 Ab with human IL-15 (PDB 2xqb). (B) The surface of the Ag chain is rendered according to atom charge. Due to the hydrogen bond with antigenic GLU53, TYR49 is located in high proximity to the Ag. Ab LEU46 is located in proximity to antigenic LEU52. (C) Seven residues from L2 (green, solid ribbon) interact with the Ag (orange, solid ribbon). Two of them (LEU46 and TYR49) are not identified as part of the CDR by any other CDR identification method. These Paratome-unique residues and the antigenic residues they contact (LEU52 and GLU53) are depicted in sticks. LEU46 forms a hydrophobic interaction with LEU52. TYR49 forms a hydrogen bond with antigenic GLU53 as well as a cation- п interaction with ARG53 of the Ab. Both contribute substantially to Ag binding (see text).

To gain insight into the extent to which Paratome-unique residues contribute to Ag binding, we searched the non-redundant set of Abs for Ag binding residues residing within structural consensus regions that are not identified by any of the CDR identification methods. We obtained 153 Paratome-unique residues, originating from 104 Abs (Table S3). Using the FoldX algorithm [21], [22], we performed an in-silico alanine scan in which each Paratome-unique residue and each Ag binding residue identified by the CDR identification methods (2707 residues) within the 104 Abs were mutated to Alanine. Additionally, we searched the non-redundant set of Abs for Ag binding residues residing within CDRs that are not identified by Paratome (i.e. CDRs-unique residues). We found 59 CDRs-unique residues, stemming from 41 Abs (Table S4). To each CDRs-unique residue we performed an in-silico alanine scan in which it was mutated to Alanine. The distribution of the predicted interaction energy (ΔΔG) of these mutants is presented in figure 6A. Destabilizing residues in this analysis (ΔΔG>0.25) are residues whose mutation to alanine is predicted to destabilize the Ab-Ag complex. These residues, therefore, are likely to be important for Ag binding. Paratome-unique residues have a slightly higher percentage of destabilizing residues (49%) than Ag binding residues that fall within the CDRs according to Kabat, Chothia or IMGT (44.15%). While it is not clear whether the differences between Paratome-unique and Ag binding residues within the CDRs are significant, it is obvious that the former are at least as important to stability as the latter. In contrast, CDRs-unique residues have substantially lower contribution to binding: only 27% of them are destabilizing and the vast majority of them (70%) are neutral. To demonstrate the importance of Paratome-unique residues we show a more detailed analysis of the complex of IL-15 with an anti-IL-15 Ab (PDB ID 2xqb). Two Ag binding residues, LEU46 and TYR49, which were identified by Paratome to be part of ABR L2, were not identified by any of the CDR identification methods (Table S1). Figure 6B shows these residues relative to the surface of the Ag. It can be seen that TYR49 protrudes into the surface of the Ag, while LEU46 is located opposite to the antigenic LEU52, forming a hydrophobic interaction. As shown is Figure 6C, only seven residues from L2 interact with the Ag, and two of them are Paratome-unique residues. TYR49 forms one of the two hydrogen bonds between the Ag and ABR L2. The results of the FoldX in-silico single-point mutations analysis indicate that mutating ARG50, ARG53 and TYR49 to Alanine have the most significant destabilizing effect (Table S2). Not surprisingly, due to the salt bridge it forms with antigenic GLU46, mutating ARG50 had the most prominent destabilizing effect. The next most destabilizing mutation to Alanine was of TYR49 which forms a hydrogen bond between ABR L2 and antigenic GLU53. The third most destabilizing mutation to Alanine was of ARG53, which forms a cation-п interaction with TYR49. As expected, mutating LEU46 to Alanine has a weak destabilizing effect on the binding energy. Hence, Ag binding residues within the structural consensus regions that fall outside the CDRs may play a pivotal role in Ag binding and recognition. The amino acid composition of Paratome-unique residues is presented in Table S8.

## Discussion

Ab-Ag recognition is the basis for the vast usage of Abs for molecular identification in research and in the clinic [23]–[26]. Thus, identifying Ag binding sites facilitates the understanding of the underlying biology as well as Ab design and engineering. In a previous study [19], we have shown that structural analysis can lead to the identification of residues that roughly correspond to the CDRs. Here we further developed this approach, and tried to determine whether it can be used to identify the Ag binding regions within Abs. To our knowledge, this study is the first to quantitatively compare the residues identified by the most commonly used CDR identification methods. The residues that reside within the structural consensus regions cover most of the observed Ag binding residues (94%), a significantly higher coverage than with the other methods. The coverage obtained by Kabat, Chothia and IMGT stemmed almost entirely from the residues that were within the structural consensus regions. While CDR residues unique to Kabat, Chothia and IMGT comprised less than 2% of the Ag binding sites, ABRs residues unique to Paratome covered 10–17% of the Ag binding residues. Nevertheless, there are cases in which the structural consensus regions did not contain Ag binding residues while a CDR identification method identified them. For a detailed example, see Figure S1. Approximately 2% of the Ag contacting residues are located remotely from the ABRs/CDRs and thus should be considered as true negatives. Therefore, the actual recall of Paratome is 96%.

Interestingly, all Paratome-unique residues come from either L2 or H2. However, when we compare each method separately to Paratome there are differences in other CDRs as well. Table S9 shows the distribution of CDRs from which the Ag binding residues that are identified by Paratome but are missed by one of the other methods originated, in a pairwise comparison.

MacCallum et al. [8] demonstrated that for some of the CDRs, the residues that contact the Ag correspond better with the Kabat definition of CDRs than with that of Chothia. This finding may, to some extent, explain the fact that the ABRs residues have the highest overlap with the residues identified by Kabat, and that for both H2 rather than H3, comprises the largest number of residues. Attempts to increase Ab affinity have suggested that CDRs L3 and H3 are prevalently responsible for high energy interactions with the Ag [27], [28]. This coincides with our observation that ABRs/CDRs L3 and H3 have the largest fraction of Ag binding residues for both Paratome and Kabat. For Chothia and IMGT, however, the CDRs with most Ag contacting residues are H2 and H3 (Figure 5A). Notably, for all methods except for Chothia, H2 and H3 rather than L3 and H3, cover a significantly larger percentage of the Ag binding residues (Figure 5B).

This analysis of Ag binding residues recognition demonstrates that relying on structural consensus rather than sequence differences, enables to identify Ag binding residues significantly better than the commonly used CDR identification methods. Additionally, a detailed in-silico single point mutation analysis of all Ag binding residues demonstrates that Paratome-unique residues contribute to Ag binding at least as much as residues within the CDRs and substantially more than Ag binding residues that are not identified by Paratome and are identified by CDR identification methods. This may prove useful for applications aimed at identifying and manipulating Ag binding residues.

## Materials and Methods

### Extraction of 3D structures

The outline of our structure-based ABRs identification method is delineated in Figure 1. To identify all Ab-Ag structures in the PDB [29] we performed a BLAST [30] search against the August 2009 version of the PDB using an arbitrarily chosen Fab sequence as a query. The search was performed separately for the light and heavy chains and thus two lists were obtained, a heavy chains list (2000 chains from 962 structures) and a light chains list (2500 chains from 1047 structures). To obtain an E-value cut-off that will ascertain that the hits for the light chain do not contain any heavy chains and vice versa, we performed a BLAST search using the heavy chain of the query Fab against the hits of light chain and another BLAST search using the light chain of the query Fab against the hits of the heavy chain. Based on these analyses we determined that results with an E-value≤1e-6 should be further analysed (1280 heavy chains from 855 structures and 1846 light chains from 961 structures remained). To discard all T-cell receptors or MHC molecules complexes from our lists, we searched for a BLAST E-value that will exclude all T-cell receptors and MHC molecules from the dataset. We arbitrarily chose MHC-I, MHC-II, TCR type A and TCR Type B sequences and performed a separate BLAST search against the hits of light and heavy chains. Results with an E-value of 1e-6, 1e-6, 1e-12 and 1e-28, respectively, or smaller, were discarded. Furthermore, we removed files that contained the keywords TcR or MHC, duplicate chains from the same PDB and complexes that did not contain both a heavy Ab chain and a light Ab chain. This resulted in a list containing 1568 Ab chains from 784 structures. We then screened the list so each complex holds one heavy Ab chain, one light Ab chain, and a single Ag chain which is not an Ab and contains at least five amino acids. We did not include non-peptide Ags in the analysis. The final list from which we removed redundancy contained 352 structures.

### Redundancy removal

Redundancy removal was performed using Blastclust [31] with sequence identity ≥97% and coverage ≥95%. We ran Blastclust separately for the sequences of the light chains and for the sequences of the heavy chains and obtained 96 clusters, 48 for each. To determine which sequences to remove in each cluster, we chose the Ab-Ag interactions as the distinguishing criterion for redundancy removal. For each PDB complex in a given Blastclust cluster, we identified all residue-residue contacts (see below for contact definition). The similarity between any two complexes (i.e., lists of Ab-Ag contacts) within each cluster was measured as the number of identical contacts (i.e., the same amino acid and alignment position within the Ab and the same amino acid and position within the Ag) divided by the total number of contacts in the shorter of the two lists. Since the similarity score on its own is not sufficient for separating the non-redundant complexes from the redundant ones, we plotted a histogram of the similarity scores to obtain a discriminating cut-off. Most of the complexes in any given Blastclust cluster had a similarity score greater than 0.90 while only 25% of all complexes had a similarity score smaller than 0.77. Therefore the latter was chosen as the cut-off, rendering complexes with a similarity score <0.77 non-redundant. For each group of complexes with a similarity score above the cut-off, the complex with the highest number of interactions was chosen as the representative complex. This process removed 152 redundant complexes and the resulting non-redundant set included 200 experimentally determined 3-D structures of Ab-Ag complexes from the PDB.

### Ag Binding Regions identification - Paratome

Using a structure-based approach [19] that is presented in Figure 1, we determined the ABRs of the Abs in our dataset of non-redundant known Ab-Ag complexes from the PDB. The algorithm structurally aligns all Abs whose 3-D structure was experimentally determined bound to their protein Ag, using the MUSTANG multiple structure alignment algorithm [32]. Next, it marks the residues in each structure that contact the residues on the Ag. Then, it searches for structurally aligned positions that create such contacts across at least 10% of the Abs. These positions form six sequence stretches along the Ab sequence that correspond to the six CDRs. Beyond the edges of these clusters, there were no structurally aligned positions in which more than 10% of the Abs created contacts with the Ag. Therefore, these were defined as the ABRs edges. Applying this algorithm to the dataset, we automatically identified all ABRs defined by our method without any manual intervention.

### CDR identification - Kabat, Chothia, and IMGT

Kabat, Chothia and IMGT establish Ab sequence numbering schemes that define in a straightforward manner the location of the CDRs within the sequence. Applying the various numbering schemes to the Ab sequences in our dataset we obtained the residues composing the CDRs according to each of the methods. We used the online AbNum tool [8] to number the Abs in our dataset according to Kabat and Chothia. The boundaries of the CDRs were defined as described in AbNum (see table of CDR definitions [33]). To obtain the CDRs according to IMGT, we coupled the Kabat numbering obtained by applying AbNum with a conversion code available at the IMGT web site.

### Automatic sequence based ABRs identification

Considering that the three dimensional structure of most known Abs is not yet known, the ability to identify the ABRs based merely on its sequence, is highly desirable. We constructed an automated ABRs identification tool capable of identifying the ABRs of an Ab from its amino acid sequence. Given a query sequence, the tool works as follows: First, a BLAST search is performed using the query Ab sequence, against all Abs in our dataset. As described above, all Abs in this non-redundant set were annotated and the ABRs within each of them were identified based on the multiple structure alignment (MSTA). The best hit from this BLAST search (i.e., lowest E-value) is then used to infer the positions of the ABRs in the query sequence from the annotated sequence. Since our aim is to identify the ABRs, aligning the entire input Ab sequence, including its yet unidentified stretches of ABRs, may lead to misalignments. Therefore, standard application of global sequence alignment algorithms is not suitable for this task. To overcome this, we align only the FRs, using the Smith-Waterman local alignment algorithm [34]. This is based on the premise that the FRs originate from a limited set of genes and undergo fewer mutations than the highly variable ABRs [25]. Therefore aligning the FRs is less error-prone than aligning the entire Ab. The FRs of the annotated sequence are identified based on the location of the ABRs in the MSTA, which in turn is used to identify the FRs of the query sequence and thus identifying the ABRs. Figure 2A summarizes the process of sequence based ABRs identification.

### Automatic structure based ABRs identification

When the query Ab has a known 3-D structure, it can be used to identify the ABRs. The first stage in this case is, once again, to identify the top BLAST hit for the sequence of the query Ab. Next, the query Ab and the top BLAST hit in the non-redundant Abs set are structurally aligned using the Combinatorial Extension (CE) algorithm [35]. Finally, the MSTA and the pairwise structural alignment are used to transfer the locations of the ABRs from the annotated Ab structure to the structure of the query Ab. Figure 2B summarizes the process of structure based ABRs identification.

### Generation of the test dataset

To assess the identification of Ag binding sites, we curated a test set of Ab-Ag complexes that were not used to construct our tool. We started by recording all Ab complexes added to the PDB between September 1^st^ 2009 and February 22^nd^ 2011 (8522 entries). We identified which of these entries are Abs by performing a BLAST search against the same arbitrarily chosen light and heavy chain Ab Fabs as well as MHC-I, MHC-II, TCR-A and TCR-B sequences described previously at the process of constructing the initial dataset of Ab-Ag complexes (see Extraction of 3D structures). A PDB entry was retained if i) At least one of its chains was similar to the light chain (e-value≤2e-32), ii) At least one of its other chains (belonging to the same biological unit, as defined by remark 350 within the PDB file) was similar to the heavy chain (e-value≤2e-26) and iii) Neither of these chains was similar to any of the MHC or TCR molecules. The resulting list was further analysed to include only interacting Ab-Ag complexes. An Ab-Ag complex was defined as interacting if at least one of the atoms in the Ab molecule was ≤6 Å from any atom of the Ag molecule [36]. The final list contains 69 distinct Ab-Ag complexes.

### Extraction of Ag binding residues

For each Ab in the test set of 69 Ab-Ag complexes, we identified all Ab residues that contact the Ag (see next paragraph for definition of contact). Then we applied to each Ab the implementation of the CDR identification tools of Kabat, Chothia and IMGT. Finally, we used the structure based approach described above to identify the ABRs. Thus, each Ab in the test dataset has five lists, the list of all Ab binding residues (“gold standard”) and four lists of identified residues, one for each of the compared methods. Applying the sequence based approach presented above to the test set resulted in identification of a set of Ag binding residues that is 99% identical to the set identified using the structural approach.

An Ab amino acid and an Ag amino acid are considered as interacting if at least one of their respective atoms were ≤6 Å of each other [36]. While this is a permissive cut-off that introduces into the dataset many non-interacting residues, it allows for an unbiased dataset [37]. To demonstrate that the results are not sensitive to this choice, we repeated the analysis also for distance cut-offs of 4 Å, 4.5 Å, 5 Å and 5.5 Å (Figure S2). The results show that the recall of Paratome remains superior to that of all other methods, and that the inevitable changes in recall and precision that stem from changing the positive set have the same effect on all the methods.

### Comparing regions identified by different methods

For each method and for each Ab within the test dataset we recorded:

1. The number of residues identified within each of the six regions (ABR/CDR).
2. The percentage of ABR/CDR residues that contact the Ag out of the total number of ABR/CDR residues.
3. The fraction of ABR/CDR residues that contact the Ag out of the entire set of Ab residues contacting the Ag.

**Figure 7:**
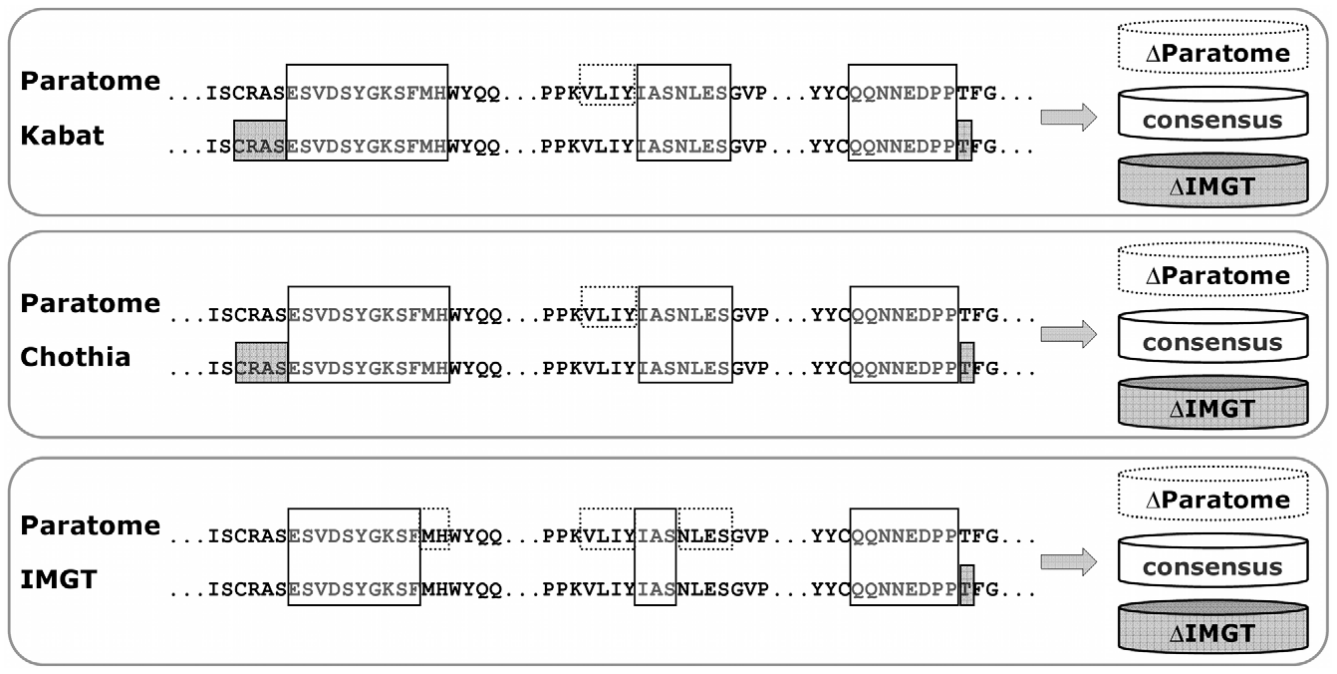
Comparison of consensus and method-specific residues. A light chain is analysed by all methods. In the top comparison, the residues identified by Paratome (grey, top line) are compared to those identified by Kabat (grey, second line). For L1, for example, both methods agree on the fragment ESVDSYGKSFMH, however, according to Kabat, CDR L1 includes also three amino acids N-terminal to this fragment (RAS, marked with a grey box) while according to Paratome these are not included in ABR L1. ABR L2, is identified by Paratome to be longer than the CDR L2 identified by Kabat by four residues (VLIY, marked with a dashed box).

We then performed a pairwise comparison between Paratome and each of the other methods. We examined three sets of residues: the consensus residues (i.e. residues identified by both our method and the other method), and two sets of method-specific residues (i) residues identified by Paratome yet not by the other method (ΔParatome) and (ii) residues identified by the other method yet not by Paratome (e.g., ΔKabat.). For each of the three sets, we recorded the number of residues it includes, the number of Ab binding residues and what fraction of the Ag binding sites is covered by these residues. Figure 7 shows an example of the comparison of method-specific residues and consensus residues for all methods, for one Ab light chain.

All data are available in tables S6 and S7.

### Assessing performance

To assess the performance of the extracted sets according to each of the methods, we computed for each Ab, the precision:
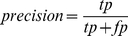
(1)where tp is the number of true positive predictions, namely the number of residues predicted to bind the Ag that were observed in the structure to be in contact with the Ag. fp (false positive) is the number of residues predicted to bind the Ag that were not observed to be in contact with the Ag. Measures such as precision are typically complemented by the recall measure, defined as:
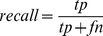
(2)where fn is the number of false negative predictions (i.e. residues not predicted to bind the Ag that were observed to be in contact with the Ag). The average recall and precision were computed for the Abs in the test set.

### Extraction of Paratome-unique and CDRs-unique residues

The ABRs/CDRs were obtained for each of the Abs in the non-redundant train set according to Kabat, Chothia, IMAGT and Paratome as described above. Next, all Ag binding residues were extracted from the PDB structure, and mapped to the ABRs and CDRs. We then searched for Ag binding residues that are identified by Paratome but not by any CDR identification method. Next, we searched for Ag binding residues that are identified by any of the CDR identification methods but not by Paratome (Table S3, S4 and S5).

### Single-point mutations analysis

To assess the contribution of Paratome-unique Ag binding residues to Ab-Ag binding, we used the FoldX algorithm [21], [22], a molecular modelling software that computationally predicts the effect of mutations on the binding energy. It is based on empirical energy terms correlated with experimental ΔΔG measurements [21]. We computed the effect of mutating each Ag binding residue to Alanine on Ab-Ag binding energy (ΔΔG). We applied FoldX (version 3.0b4) to all Paratome-unique residues (Table S3) and to all CDRs-unique residues (Table S4), in the following manner: i) 3D structures were taken from the PDB (PDB accession numbers and relevant chains are listed in Table S5) and optimized using the FoldX repairPDB function, ii) Structures corresponding to each of the single-point mutants were generated using the FoldX BuildModel protein mutagenesis function, iii) The interaction energy of the WT structure and the mutated structure with the Ag were calculated using the ComplexAnalysis energy calculation function of FoldX, iv). ΔΔG values were obtained using the following equation:
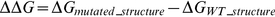
(3)Mutations for which ΔΔG>0.25 were defined as destabilizing mutations, whereas mutations for which ΔΔG<−0.25 were defined as stabilizing. Mutations were defined as neutral if 0.25≥ΔΔG≥−0.25.

## Supporting Information

Figure S1 **Ag binding residues not identified by Paratome (PDB 1kb5).** (A) The definition of ABR H2 according to Paratome. The distance of GLN61 (Ab heavy chain) is less than 6 Å from VAL57 and LYS55 on the Ag. Nevertheless, GLN61 is erroneously not defined to be a part of H2 according to Paratome. (B) The definition CDR H2 by Kabat. Kabat's definition of H2 identifies GLN61 to be part of the CDR.
(TIF)

Figure S2 **Recall and precision of Ag binding sites identification using various distance cutoffs.** An Ab amino acid and an Ag amino acid were defined as interacting if at least one of their respective atoms were ≤6 Å of each other. To demonstrate that the superior performance of Paratome does not stem from using this permissive cutoff, average precision and recall were computed for the Abs in the test set for all methods, using various distance cutoffs. Error bars represent standard error of the mean. (A) Recall and precision for a 4 Å cutoff. (B) Recall and precision for a 4.5 Å cutoff. (C) Recall and precision for a 5 Å cutoff. (D) Recall and precision for a 5.5 Å cutoff.
(TIF)

Table S1 **ABRs, CDRs and Ag binding residues of anti IL-15 Ab (PDB ID 2xqb) according to Paratome, Kabat, Chothia and IMGT.**
(PDF)

Table S2 **The effect of mutating ABR L2 Ag binding residues to Alanine on the binding energy (ΔΔG) between the Ab and the Ag (PDB ID 2xqb).**
(PDF)

Table S3 **The list of Paratome unique residues within the train set on which we performed our in-silico alanine scan analysis.**
(PDF)

Table S4 **The list of CDRs unique residues within the train set on which we performed our in-silico alanine scan analysis.**
(PDF)

Table S5 **The list of ABRs and CDRs for which we performed the in-silico alanine scan analysis as well as which residues are Ag binding residues.**
(PDF)

Table S6 **Train dataset.** Contains the list of PDB structures used to construct Paratome.
(PDF)

Table S7 **Test dataset.** Contains the list of PDB structures used to test Paratome.
(PDF)

Table S8 **The amino acid composition of Paratome-unique Ag binding residues.**
(PDF)

Table S9 **The percentage of Paratome-unique residues for each ABR/CDR.**
(PDF)
