# Supplementary figures and images for: Structural Consensus among Antibodies Defines the Antigen Binding Site
Source: PLoS Comput Biol. 2012 Feb 23;8(2):e1002388. doi: 10.1371/journal.pcbi.1002388 (PMC3285572; doi:10.1371/journal.pcbi.1002388)

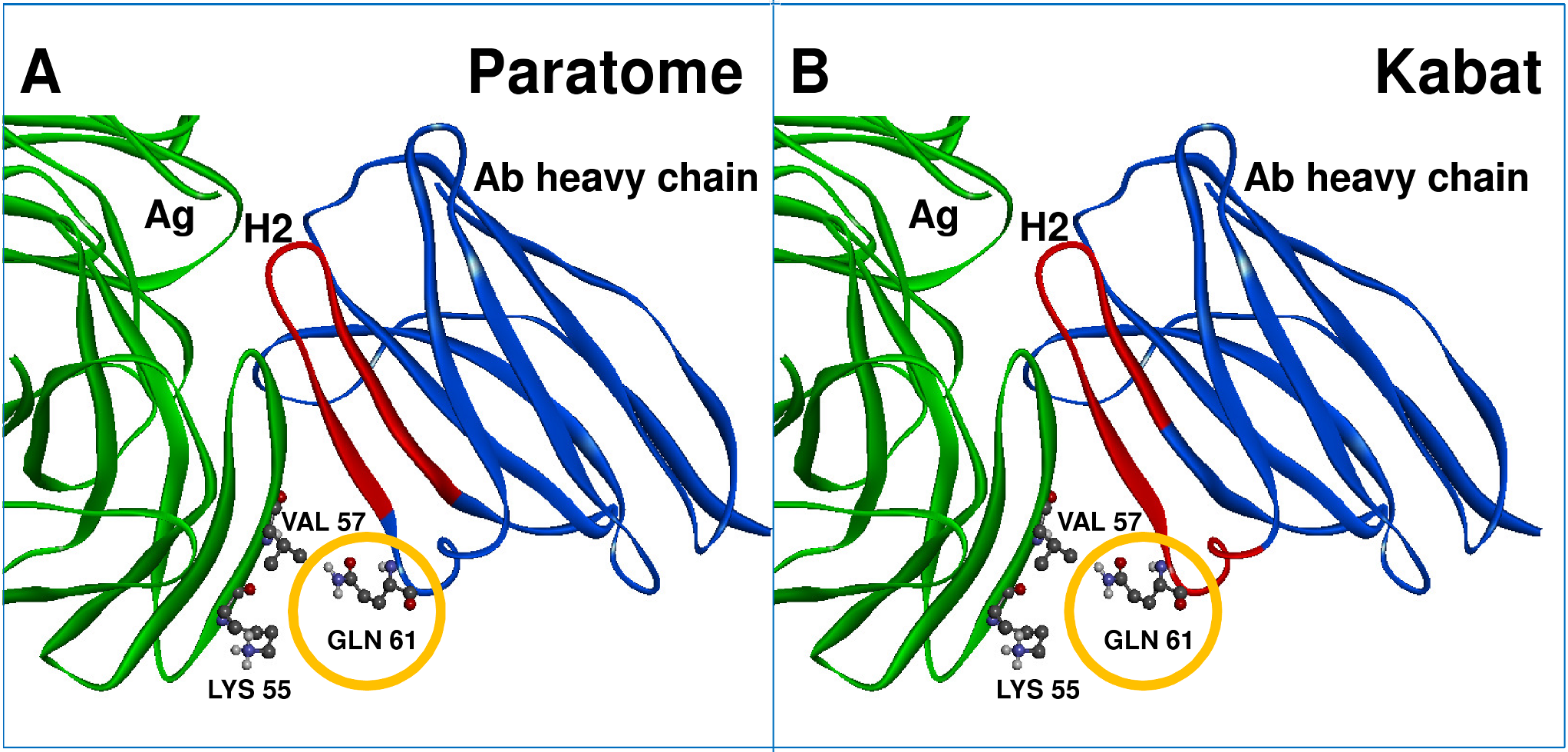

Supplement: Figure S1 — Ag binding residues not identified by Paratome (PDB 1kb5). (A) The definition of ABR H2 according to Paratome. The distance of GLN61 (Ab heavy chain) is less than 6 Å from VAL57 and LYS55 on the Ag. Nevertheless, GLN61 is erroneously not defined to be a part of H2 according to Paratome. (B) The definition CDR H2 by Kabat. Kabat's definition of H2 identifies GLN61 to be part of the CDR. (TIF) [file pcbi.1002388.s001.tif]

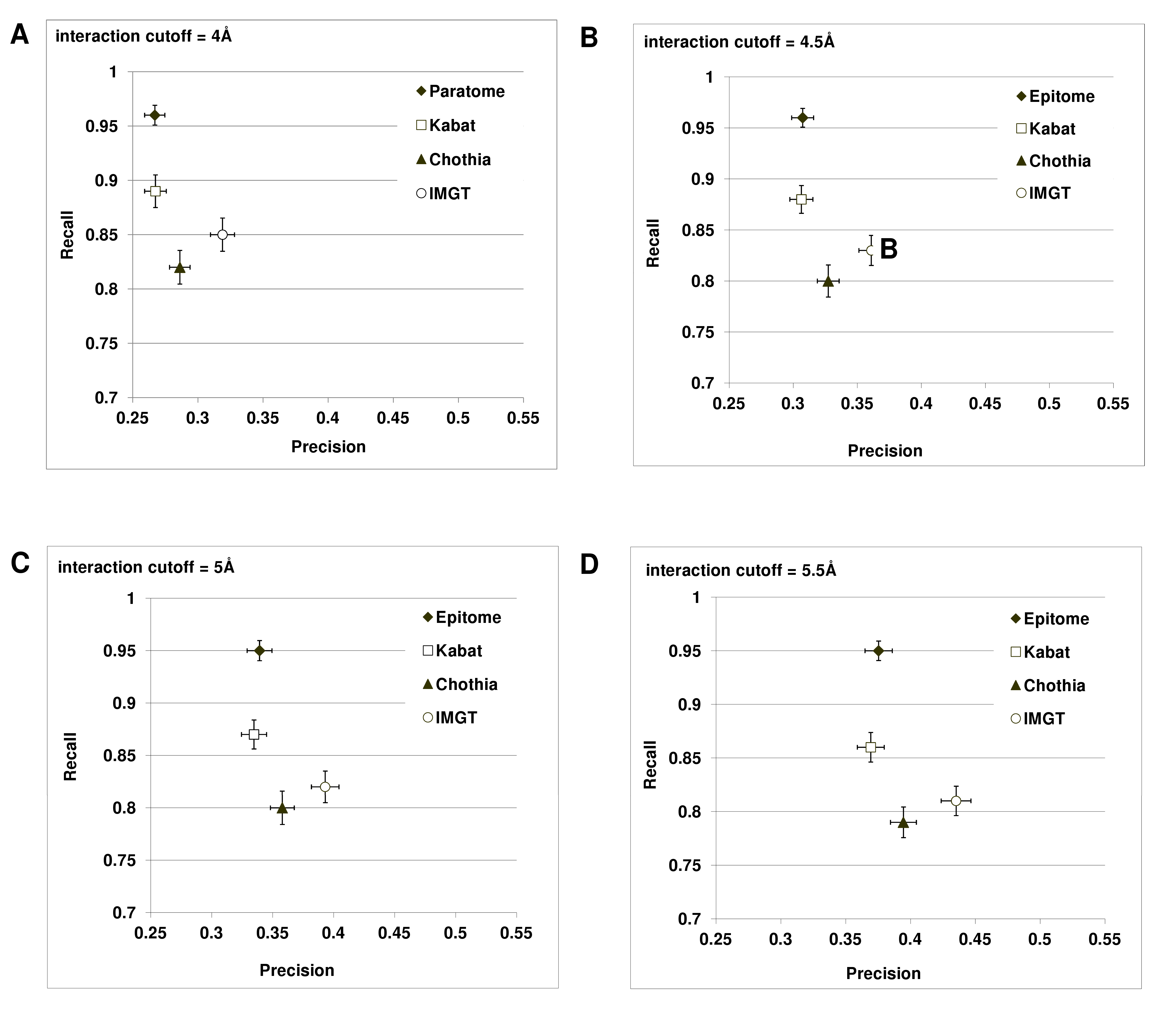

Supplement: Figure S2 — Recall and precision of Ag binding sites identification using various distance cutoffs. An Ab amino acid and an Ag amino acid were defined as interacting if at least one of their respective atoms were ≤6 Å of each other. To demonstrate that the superior performance of Paratome does not stem from using this permissive cutoff, average precision and recall were computed for the Abs in the test set for all methods, using various distance cutoffs. Error bars represent standard error of the mean. (A) Recall and precision for a 4 Å cutoff. (B) Recall and precision for a 4.5 Å cutoff. (C) Recall and precision for a 5 Å cutoff. (D) Recall and precision for a 5.5 Å cutoff. (TIF) [file pcbi.1002388.s002.tif]
