# Supplementary material for: Structural Consensus among Antibodies Defines the Antigen Binding Site
Source: PLoS Comput Biol. 2012 Feb 23;8(2):e1002388. doi: 10.1371/journal.pcbi.1002388 (PMC3285572; doi:10.1371/journal.pcbi.1002388)
Supplement: Table S1 — ABRs, CDRs and Ag binding residues of anti IL-15 Ab (PDB ID 2xqb) according to Paratome, Kabat, Chothia and IMGT. (PDF) [file pcbi.1002388.s003.pdf]

We extracted the ABRs and CDRs according to each of the CDR identification methods as well as the Ag binding residues of PDB ID 2xqb (see Materials and Methods). Table S1 depicts the obtained ABRs and CDRs.

Table S1. ABRs and CDRs of anti IL-15 Ab (PDB ID 2xqb) according to each of the identification methods.

	Paratome	Kabat	Chothia	IMGT
H1	YSFSSFGIS	SFGIS	GYSFSSF	GYSFSSFG
H2	WLGWISAFNGYTKY	WISAFNGYTKYAQKFQD	SAFNGY	ISAFNGYT
H3	RDPAAWPLQQSLAWFDP	DPAAWPLQQSLAWFDP	DPAAWPLQQSLAWFDP	ARDPAAWPLQQSLAWFDP
L1	TSNLKRNYVY	SGSTSNLKRNYVY	SGSTSNLKRNYVY	TSNLKRNY
L2	LLIYRDRRRPS	RDRRRPS	RDRRRPS	RDR
L3	AWYDRELSEW	AWYDRELSEWV	AWYDRELSEWV	AWYDRELSEWV
