# Supplementary material for: Structural Consensus among Antibodies Defines the Antigen Binding Site
Source: PLoS Comput Biol. 2012 Feb 23;8(2):e1002388. doi: 10.1371/journal.pcbi.1002388 (PMC3285572; doi:10.1371/journal.pcbi.1002388)
Supplement: Table S2 — The effect of mutating ABR L2 Ag binding residues to Alanine on the binding energy (ΔΔG) between the Ab and the Ag (PDB ID 2xqb). (PDF) [file pcbi.1002388.s004.pdf]

ABR L2 (i.e. LLIYRDRRRPS) of PDB ID 2xqb was the only ABR for which off-CDRs residues were found. To examine the effect of mutating to Alanine the off-CDRs residues on the binding energy, and thus on the stability of the Ab-Ag complex, we performed an in-silico single-point mutations analysis using the FoldX algorithm (see Methods), to L2 ABR residues. Table S2 shows the results of mutating L2 ABR residues to Alanine on the binding energy (ΔΔG) between the Ab and the Ag.

Table 2S. The effect of mutating ABR L2 Ab binding residues to Alanine on the binding energy.

Ab residue	ΔΔG	Effect of mutation
**LEU46**	**0.32**	**NEUTRAL**
LEU47	0	NEUTRAL
ILE48	-0.04	NEUTRAL
**TYR49**	**2.59**	**DESTABILIZING**
**ARG50**	**3.77**	**DESTABILIZING**
ASP51	-0.18	NEUTRAL
ARG52	0.35	DESTABILIZING
**ARG53**	**1.71**	**DESTABILIZING**
ARG54	0.36	DESTABILIZING
PRO55	0.34	DESTABILIZING
SER56	0.31	DESTABILIZING
