# Supplementary material for: Structural Consensus among Antibodies Defines the Antigen Binding Site
Source: PLoS Comput Biol. 2012 Feb 23;8(2):e1002388. doi: 10.1371/journal.pcbi.1002388 (PMC3285572; doi:10.1371/journal.pcbi.1002388)
Supplement: Table S3 — The list of Paratome unique residues within the train set on which we performed our in-silico alanine scan analysis. (PDF) [file pcbi.1002388.s005.pdf]

We searched the train dataset (see table S6) for Paratome unique residues and found 153 such residues originating from 104 Abs.. Table S3 lists the obtained Paratome unique residues.

Table S3. The list of Paratome unique residues within the train set on which we performed our in-silico alanine scan analysis.

PDB ID	chain	location	Residue type
**1A3R**	L	46	L
**1A3R**	L	49	Y
**1ADQ**	L	49	Y
**1AFV**	H	47	W
**1AR1**	D	49	Y
**1BGX**	L	46	L
**1BGX**	L	49	Y
**1BGX**	L	47	L
**1BGX**	L	48	I
**1BGX**	H	47	W
**1BOG**	A	49	Y
**1BOG**	B	49	G
**1BOG**	B	47	W
**1BVK**	A	49	Y
**1CE1**	L	49	Y
**1CFN**	A	49	Y
**1CFS**	A	49	Y
**1CFS**	A	46	T
**1CFS**	A	48	I
**1CFT**	A	49	Y
**1CU4**	L	49	F
**1CU4**	L	46	R
**1CZ8**	H	47	W
**1DQJ**	A	49	K
**1E4W**	L	49	Y
**1E4W**	L	46	L
**1E4X**	L	49	Y
**1E4X**	L	46	L
**1EO8**	L	49	Y
**1F58**	H	47	W
**1FE8**	L	49	Y
**1FJ1**	A	49	H
**1FJ1**	A	46	L
**1FNS**	H	261	W
**1GGI**	L	49	Y
**1GGI**	L	46	L
**1GGI**	H	47	W
**1H0D**	B	47	W
**1HH9**	A	49	Y
**1HH9**	B	47	W

**1HI6**	A	49	Y
**1HI6**	A	46	T
**1HI6**	A	48	I
**1HI6**	B	49	G
**1IQD**	A	50	Y
**1IQD**	B	47	W
**1J1P**	L	49	K
**1JHL**	L	49	Y
**1KB9**	K	49	Y
**1KC5**	H	48	W
**1KCR**	L	49	Y
**1KCR**	H	48	W
**1KCS**	H	48	W
**1KEN**	L	50	Y
**1KTR**	H	54	W
**1LK3**	H	47	W
**1MHP**	H	47	W
**1MLC**	B	47	W
**1MPA**	H	47	W
**1N64**	H	47	W
**1N8Z**	A	49	Y
**1NAK**	L	46	L
**1NAK**	L	49	Y
**1NAK**	H	49	G
**1NAK**	H	47	Y
**1NCD**	L	49	Y
**1NDG**	A	49	K
**1NDM**	A	49	K
**1NFD**	G	49	Y
**1NL0**	H	47	W
**1NSN**	L	49	K
**1OSP**	L	49	S
**1OTS**	D	48	Y
**1P2C**	B	347	W
**1P4B**	L	56	I
**1P4B**	L	57	G
**1PKQ**	A	49	Y
**1PZ5**	B	47	W
**1QKZ**	H	49	A
**1QKZ**	H	47	W
**1S78**	C	46	L
**1S78**	C	49	Y
**1TET**	H	47	W
**1TPX**	C	49	Y
**1TPX**	C	46	R
**1TPX**	B	47	W
**1UAC**	L	49	K
**1V7M**	L	45	L
**1V7M**	L	48	Y

**1VFB**	A	49	Y
**1XCQ**	B	47	W
**1XCT**	B	47	W
**1XGU**	A	49	K
**1XGY**	L	49	Y
**1XGY**	H	47	W
**1YQV**	H	47	W
**1YY9**	C	49	K
**2A6I**	A	49	Y
**2A6I**	B	47	W
**2ADF**	L	49	H
**2ADF**	H	47	W
**2AEP**	L	49	Y
**2ARJ**	L	49	Y
**2B2X**	L	48	Y
**2B2X**	H	47	W
**2BDN**	L	49	S
**2CK0**	H	47	W
**2CMR**	H	47	W
**2EH8**	L	49	Y
**2EH8**	L	46	R
**2EH8**	H	47	W
**2FJH**	L	49	Y
**2FJH**	H	47	W
**2FX7**	H	49	G
**2FX7**	H	47	W
**2H9G**	A	49	Y
**2HFG**	L	49	Y
**2HH0**	L	54	R
**2HKF**	L	54	Y
**2IFF**	H	47	W
**2IGF**	H	47	W
**2IPU**	H	47	W
**2J88**	L	49	Y
**2J88**	L	48	V
**2NR6**	C	49	Y
**2NR6**	D	47	W
**2OR9**	L	46	L
**2OR9**	L	49	Y
**2OSL**	H	49	G
**2OSL**	H	47	W
**2OTU**	A	49	E
**2OZ4**	L	49	E
**2OZ4**	L	46	L
**2Q8A**	L	49	Y
**2Q8A**	L	46	L
**2R0K**	H	47	W
**2R0L**	H	47	W
**2R0W**	H	47	W

**2R29**	L	50	L
**2R29**	L	53	Y
**2R4S**	L	49	Y
**2R4S**	L	46	T
**2R4S**	L	48	I
**2R56**	L	49	Y
**2V17**	L	49	Y
**2VDK**	L	49	Y
**2VWE**	C	49	Y
**3BKJ**	H	47	W
**3BKY**	H	47	W
**3BT2**	H	47	W
**3CXD**	L	49	Y
**3CXD**	L	46	L
**3CXD**	L	48	V
