# Supplementary material for: Structural Consensus among Antibodies Defines the Antigen Binding Site
Source: PLoS Comput Biol. 2012 Feb 23;8(2):e1002388. doi: 10.1371/journal.pcbi.1002388 (PMC3285572; doi:10.1371/journal.pcbi.1002388)
Supplement: Table S4 — The list of CDRs unique residues within the train set on which we performed our in-silico alanine scan analysis. (PDF) [file pcbi.1002388.s006.pdf]

We searched the train dataset (see table S6) for CDRs unique residues and found 59 such residues. Table S4 lists the obtained CDRs unique residues.

Table S4. The list of CDRs unique residues within the train set for which we performed our in-silico alanine scan analysis.

PDB ID	Chain type	Location	Residue type
**1A3R**	HEAVY	93	D
**1ADQ**	HEAVY	26	G
**1AHW**	HEAVY	65	Q
**1AR1**	HEAVY	26	G
**1BGX**	HEAVY	63	K
**1BGX**	HEAVY	64	S
**1BGX**	HEAVY	61	S
**1BGX**	HEAVY	62	L
**1BGX**	HEAVY	26	G
**1EO8**	HEAVY	26	G
**1FBI**	HEAVY	65	K
**1FSK**	HEAVY	97	T
**1HI6**	HEAVY	97	T
**1IQD**	HEAVY	26	G
**1JPS**	HEAVY	65	Q
**1JPS**	HEAVY	62	P
**1JRH**	HEAVY	61	P
**1KB5**	HEAVY	64	K
**1KB5**	HEAVY	61	Q
**1KB9**	HEAVY	26	G
**1KTR**	HEAVY	107	E
**1NCD**	HEAVY	64	K
**1NCD**	HEAVY	61	E
**1OAZ**	HEAVY	26	G
**1QFU**	HEAVY	26	G
**1S78**	HEAVY	64	K
**1S78**	HEAVY	61	Q
**1S78**	HEAVY	60	N
**1SM3**	HEAVY	26	G
**1UJ3**	HEAVY	365	Q
**1XCQ**	HEAVY	26	G
**1XGY**	HEAVY	93	A
**1YNT**	HEAVY	562	Q
**1ZA3**	HEAVY	63	V
**1ZA3**	HEAVY	64	K
**1ZA3**	HEAVY	61	D
**1ZA3**	HEAVY	65	G
**1ZA3**	HEAVY	62	S
**1ZTX**	HEAVY	26	G
**2A6I**	HEAVY	97	A
**2B2X**	HEAVY	60	L
**2B4C**	HEAVY	64	R
**2B4C**	HEAVY	61	Q

**2BDN**	HEAVY	26	G
**2BOC**	HEAVY	62	E
**2EH8**	HEAVY	93	A
**2HH0**	HEAVY	137	D
**2HH0**	HEAVY	135	K
**2HH0**	HEAVY	108	R
**2HH0**	HEAVY	107	G
**2HKF**	HEAVY	26	G
**2OZ4**	HEAVY	65	K
**2OZ4**	HEAVY	26	G
**2Q8A**	HEAVY	26	G
**2R0K**	HEAVY	26	G
**2R29**	HEAVY	26	G
**1TJI**	LIGHT	26	S
**1YY9**	LIGHT	26	S
**2OTU**	LIGHT	25	S
