# Supplementary material for: Structural Consensus among Antibodies Defines the Antigen Binding Site
Source: PLoS Comput Biol. 2012 Feb 23;8(2):e1002388. doi: 10.1371/journal.pcbi.1002388 (PMC3285572; doi:10.1371/journal.pcbi.1002388)
Supplement: Table S5 — The list of ABRs and CDRs for which we performed the in-silico alanine scan analysis as well as which residues are Ag binding residues. (PDF) [file pcbi.1002388.s007.pdf]

Table S5. The list of ABRs and CDRs for which we performed the in-silico alanine scan analysis as well as which residues are Ag binding residues. Ag binding residues are marked in bold.

ID	Paratome	Kabat	Chothia	IMGT
**1A3R-L1**	QSLL**N**S**RT**QKN**YLT**	KSSQSLL**N**S**RT**QKN**YLT**	KSSQSLL**N**S**RT**QKN**YLT**	QSLL**N**S**RT**QKN**Y**
**1A3R-L2**	**L**LI**YW**ASTRES	**W**ASTRES	**W**ASTRES	**W**AS
**1A3R-L3**	**QNNYNY**P**L**	**QNNYNY**P**L**T	**QNNYNY**P**L**T	**QNNYNY**P**L**T
**1A3R-H1**	FNI**KDIY**I**H**	**DIY**I**H**	GFNI**KDI**	GFNI**KDIY**
**1A3R-H2**	WIG**RLDPAN**G**YTK**Y	**RLDPAN**G**YTK**YDPKFQG	**DPAN**G**Y**	**LDPAN**G**YT**
**1A3R-H3**	**GYYSYYDMD**Y	**YYSYYDMD**Y	**YYSYYDMD**Y	**DGYYSYYDMD**Y
**1ACY-L1**	ESV**DSY**GKS**F**MH	RASESV**DSY**GKS**F**MH	RASESV**DSY**GKS**F**MH	ESV**DSY**GKS**F**
**1ACY-L2**	VLIYIASNLES	IASNLES	IASNLES	IAS
**1ACY-L3**	QQ**NNEDPP**	QQ**NNEDPP**T	QQ**NNEDPP**T	QQ**NNEDPP**T
**1ACY-H1**	FSITR**TNYC**WH	R**TNYC**WH	GFSITR**TNY**	GFSITR**TN**
**1ACY-H2**	WMG**R**I**CYE**G**S**IYY	**R**I**CYE**G**S**IYYSPSIKS	**CYE**G**S**	I**CYE**G**S**I
**1ACY-H3**	R**E**N**HMYET**YFDV	**E**N**HMYET**YFDV	**E**N**HMYET**YFDV	SR**E**N**HMYET**YFDV
**1ADQ-L1**	NIGSK**S**VH	GGNNIGSK**S**VH	GGNNIGSK**S**VH	NIGSK**S**
**1ADQ-L2**	LVV**YD**DS**DRPP**	**D**DS**DRPP**	**D**DS**DRPP**	**D**DS
**1ADQ-L3**	QVWDSSSDHA	QVWDSSSDHAV	QVWDSSSDHAV	QVWDSSSDHAV
**1ADQ-H1**	F**T**F**DDY**AMH	**DY**AMH	**G**F**T**F**DDY**	**G**F**T**F**DDY**A
**1ADQ-H2**	WVSGIS**W**NTGTIIY	GIS**W**NTGTIIYADSVKG	S**W**NTGT	IS**W**NTGTI
**1ADQ-H3**	KT**RSYVV**A**AE**Y**Y**F**HY**	T**RSYVV**A**AE**Y**Y**F**HY**	T**RSYVV**A**AE**Y**Y**F**HY**	AKT**RSYVV**A**AE**Y**Y**F**HY**
**1AFV-L1**	ESV**DNYGI**S**F**MN	RASESV**DNYGI**S**F**MN	RASESV**DNYGI**S**F**MN	ESV**DNYGI**S**F**
**1AFV-L2**	LLIYAASNLGS	AASNLGS	AASNLGS	AAS
**1AFV-L3**	QQ**SKEVPL**	QQ**SKEVPL**T	QQ**SKEVPL**T	QQ**SKEVPL**T
**1AFV-H1**	YTFTSS**W**I**H**	SS**W**I**H**	GYTFTSS	GYTFTSS**W**
**1AFV-H2**	**W**IG**E**IHPNSGNT**N**Y	**E**IHPNSGNT**N**YNEKFKG	HPNSGN	IHPNSGNT
**1AFV-H3**	R**WRYGSPY**YFDY	**WRYGSPY**YFDY	**WRYGSPY**YFDY	AR**WRYGSPY**YFDY
**1AHW-L1**	**Q**DI**R**K**Y**LN	KAS**Q**DI**R**K**Y**LN	KAS**Q**DI**R**K**Y**LN	**Q**DI**R**K**Y**
**1AHW-L2**	TLIY**Y**ATSLAD	**Y**ATSLAD	**Y**ATSLAD	**Y**AT
**1AHW-L3**	LQ**HGESPY**	LQ**HGESPY**T	LQ**HGESPY**T	LQ**HGESPY**T
**1AHW-H1**	**FNIKDYY**MH	**DYY**MH	G**FNIKDY**	G**FNIKDYY**
**1AHW-H2**	WIG**L**I**DPEN**G**NTIY**	**L**I**DPEN**G**NTIY**DPKF**Q**G	**DPEN**G**N**	I**DPEN**G**NT**
**1AHW-H3**	R**DNSY**YFDY	**DNSY**YFDY	**DNSY**YFDY	AR**DNSY**YFDY
**1AR1-L1**	E**N**I**YSY**LA	RASE**N**I**YSY**LA	RASE**N**I**YSY**LA	E**N**I**YSY**
**1AR1-L2**	FLV**YN**A**KT**LG**E**	**N**A**KT**LG**E**	**N**A**KT**LG**E**	**N**A**K**
**1AR1-L3**	QH**HY**GTPPL	QH**HY**GTPPLT	QH**HY**GTPPLT	QH**HY**GTPPLT
**1AR1-H1**	**FT**FS**SY**TMS	**SY**TMS	**GFT**FS**SY**	**GFT**FS**SY**T
**1AR1-H2**	WVASIN**N**GGG**R**TYY	SIN**N**GGG**R**TYYPDTVKG	N**N**GGG**R**	IN**N**GGG**R**T
**1AR1-H3**	**RHEYY**YAMDY	**HEYY**YAMDY	**HEYY**YAMDY	V**RHEYY**YAMDY
**1BGX-L1**	SSV**SYMY**	SASSSV**SYMY**	SASSSV**SYMY**	SSV**SY**
**1BGX-L2**	**LLIYDS**T**NLAS**	**DS**T**NLAS**	**DS**T**NLAS**	**DS**T
**1BGX-L3**	QQ**WSTYPL**	QQ**WSTYPL**T	QQ**WSTYPL**T	QQ**WSTYPL**T
**1BGX-H1**	**YS**I**TSD**YA**W**N	**SD**YA**W**N	**GYS**I**TSD**Y	**GYS**I**TSD**Y
**1BGX-H2**	**W**MG**Y**I**TYSGTTDYN**	**Y**I**TYSGTTDYN**P**SLKS**	**TYSGT**	I**TYSGTT**
**1BGX-H3**	**R**Y**YYGYWYFDV**	Y**YYGYWYFDV**	Y**YYGYWYFDV**	A**R**Y**YYGYWYFDV**
**1BOG-L1**	QDINS**F**L**T**	KASQDINS**F**L**T**	KASQDINS**F**L**T**	QDINS**F**
**1BOG-L2**	TLI**YR**ANRLM**I**	**R**ANRLM**I**	**R**ANRLM**I**	**R**AN
**1BOG-L3**	LQ**YDDF**P**L**	LQ**YDDF**P**L**T	LQ**YDDF**P**L**T	LQ**YDDF**P**L**T

**1BOG-H1**	YIF**TDYEIH**	**DYEIH**	GYIF**TDY**	GYIF**TDYE**
**1BOG-H2**	**W**I**GGIH**PG**SSGTA**Y	**GIH**PG**SSGTA**YNQKFKG	**H**PG**SSG**	**IH**PG**SSGT**
**1BOG-H3**	**RKDY**	**KDY**	**KDY**	T**RKDY**
**1BVK-L1**	GNI**HNY**LA	RASGNI**HNY**LA	RASGNI**HNY**LA	GNI**HNY**
**1BVK-L2**	LLI**YY**TT**T**LAD	**Y**TT**T**LAD	**Y**TT**T**LAD	**Y**TT
**1BVK-L3**	QH**FWST**P**R**	QH**FWST**P**R**T	QH**FWST**P**R**T	QH**FWST**P**R**T
**1BVK-H1**	F**S**L**TGYG**VN	**GYG**VN	GF**S**L**TGY**	GF**S**L**TGYG**
**1BVK-H2**	WIGMI**WGDG**NTDY	MI**WGDG**NTDYNSALKS	**WGDG**N	I**WGDG**NT
**1BVK-H3**	RE**RDYR**LDY	E**RDYR**LDY	E**RDYR**LDY	ARE**RDYR**LDY
**1CE1-L1**	QNIDK**Y**LN	KASQNIDK**Y**LN	KASQNIDK**Y**LN	QNIDK**Y**
**1CE1-L2**	LLI**YN**TNNLQT	**N**TNNLQT	**N**TNNLQT	**N**TN
**1CE1-L3**	L**QHISR**P**R**	L**QHISR**P**R**T	L**QHISR**P**R**T	L**QHISR**P**R**T
**1CE1-H1**	FTFT**D**F**Y**MN	**D**F**Y**MN	GFTFT**D**F	GFTFT**D**F**Y**
**1CE1-H2**	WIG**F**I**R**DKA**K**GYTT**E**Y	**F**I**R**DKA**K**GYTT**E**YNPSVKG	**R**DKA**K**GYT	I**R**DKA**K**GYTT
**1CE1-H3**	R**EGHTAAP**FDY	**EGHTAAP**FDY	**EGHTAAP**FDY	AR**EGHTAAP**FDY
**1CFN-L1**	QDINS**F**L**T**	KASQDINS**F**L**T**	KASQDINS**F**L**T**	QDINS**F**
**1CFN-L2**	TLI**YR**ANRLMI	**R**ANRLMI	**R**ANRLMI	**R**AN
**1CFN-L3**	LQ**YDDF**PL	LQ**YDDF**PLT	LQ**YDDF**PLT	LQ**YDDF**PLT
**1CFN-H1**	YIFT**DYE**IH	**DYE**IH	GYIFT**DY**	GYIFT**DYE**
**1CFN-H2**	WIGGI**H**PGSSGTAY	GI**H**PGSSGTAYNQKFKG	**H**PGSSG	I**H**PGSSGT
**1CFN-H3**	**RKDY**	**KDY**	**KDY**	T**RKDY**
**1CFS-L1**	QDIN**SF**L**T**	KASQDIN**SF**L**T**	KASQDIN**SF**L**T**	QDIN**SF**
**1CFS-L2**	**T**L**IYR**AN**R**L**M**I	**R**AN**R**L**M**I	**R**AN**R**L**M**I	**R**AN
**1CFS-L3**	LQ**YDDF**P**L**	LQ**YDDF**P**L**T	LQ**YDDF**P**L**T	LQ**YDDF**P**L**T
**1CFS-H1**	YIFT**DYEIH**	**DYEIH**	GYIFT**DY**	GYIFT**DYE**
**1CFS-H2**	WIG**GIH**PG**S**S**GTA**Y	**GIH**PG**S**S**GTA**YNQKFKG	**H**PG**S**S**G**	**IH**PG**S**S**GT**
**1CFS-H3**	**RKD**Y	**KD**Y	**KD**Y	T**RKD**Y
**1CFT-L1**	QD**IN**S**F**LT	KASQD**IN**S**F**LT	KASQD**IN**S**F**LT	QD**IN**S**F**
**1CFT-L2**	TLI**YR**ANRLMI	**R**ANRLMI	**R**ANRLMI	**R**AN
**1CFT-L3**	LQ**YDDF**PL	LQ**YDDF**PLT	LQ**YDDF**PLT	LQ**YDDF**PLT
**1CFT-H1**	YIFT**DYE**IH	**DYE**IH	GYIFT**DY**	GYIFT**DYE**
**1CFT-H2**	WIGGI**H**PG**S**SG**TA**Y	GI**H**PG**S**SG**TA**YNQKFKG	**H**PG**S**SG	I**H**PG**S**SG**T**
**1CFT-H3**	**RKD**Y	**KD**Y	**KD**Y	T**RKD**Y
**1CU4-L1**	QSLL**DSD**G**K**T**YLI**	KSSQSLL**DSD**G**K**T**YLI**	KSSQSLL**DSD**G**K**T**YLI**	QSLL**DSD**G**K**T**Y**
**1CU4-L2**	**R**LI**FL**VSKR**D**S	**L**VSKR**D**S	**L**VSKR**D**S	**L**VS
**1CU4-L3**	**WQGTH**FP**H**	**WQGTH**FP**H**T	**WQGTH**FP**H**T	**WQGTH**FP**H**T
**1CU4-H1**	FN**IKDYY**I**Q**	**DYY**I**Q**	GFN**IKDY**	GFN**IKDYY**
**1CU4-H2**	WIG**WIDPE**NG**NSE**Y	**WIDPE**NG**NSE**YAPRFQG	**DPE**NG**N**	**IDPE**NG**NS**
**1CU4-H3**	A**DLHD**Y	**DLHD**Y	**DLHD**Y	NA**DLHD**Y
**1CZ8-L1**	QDISNYLN	SASQDISNYLN	SASQDISNYLN	QDISNY
**1CZ8-L2**	VLIYFTSSLHS	FTSSLHS	FTSSLHS	FTS
**1CZ8-L3**	QQ**YSTV**P**W**	QQ**YSTV**P**W**T	QQ**YSTV**P**W**T	QQ**YSTV**P**W**T
**1CZ8-H1**	YD**FTHYGMN**	**HYGMN**	GYD**FTHY**	GYD**FTHYG**
**1CZ8-H2**	**W**VG**WINTYT**G**EPT**Y	**WINTYT**G**EPT**YAADFKR	**NTYT**G**E**	**INTYT**G**EP**
**1CZ8-H3**	K**YPYYYGTSHW**YFDV	**YPYYYGTSHW**YFDV	**YPYYYGTSHW**YFDV	AK**YPYYYGTSHW**YFDV
**1DQJ-L1**	**QSISNN**LH	RAS**QSISNN**LH	RAS**QSISNN**LH	**QSISNN**
**1DQJ-L2**	LLI**KY**AS**Q**SIS	**Y**AS**Q**SIS	**Y**AS**Q**SIS	**Y**AS
**1DQJ-L3**	QQ**SNSW**P**Y**	QQ**SNSW**P**Y**T	QQ**SNSW**P**Y**T	QQ**SNSW**P**Y**T
**1DQJ-H1**	**D**SV**TSDY**WS	**SDY**WS	G**D**SV**TSD**	G**D**SV**TSDY**
**1DQJ-H2**	YMG**Y**I**SYSGS**T**Y**Y	**Y**I**SYSGS**T**Y**YHPSLKS	**SYSGS**	I**SYSGS**T

1DQJ-H3	SWGGDV	WGGDV	WGGDV	ASWGGDV
1E4W-L1	QDISHYLN	RASQDISHYLN	RASQDISHYLN	QDISHY
1E4W-L2	LLIYYTSTLHS	YTSTLHS	YTSTLHS	YTS
1E4W-L3	QQGGALPF	QQGGALPFT	QQGGALPFT	QQGGALPFT
1E4W-H1	FTFTNYWMH	NYWMH	GFTFTNY	GFTFTNYW
1E4W-H2	WIGEILPSNGRTNY	EILPSNGRTNYNEKFKT	LPSNGR	ILPSNGRT
1E4W-H3	RSPSDY	SPSDY	SPSDY	ARSPSDY
1E4X-L1	QDISHYLN	RASQDISHYLN	RASQDISHYLN	QDISHY
1E4X-L2	LLIYYTSTLHS	YTSTLHS	YTSTLHS	YTS
1E4X-L3	QQGGALPF	QQGGALPFT	QQGGALPFT	QQGGALPFT
1E4X-H1	FTFTNYWMH	NYWMH	GFTFTNY	GFTFTNYW
1E4X-H2	WIGEILPSNGRTNY	EILPSNGRTNYNEKFKT	LPSNGR	ILPSNGRT
1E4X-H3	RSPSDY	SPSDY	SPSDY	ARSPSDY
1E6J-L1	SSVSYMH	SASSSVSYMH	SASSSVSYMH	SSVSY
1E6J-L2	PWIYEISKLAS	EISKLAS	EISKLAS	EIS
1E6J-L3	QQWNYPF	QQWNYPFT	QQWNYPFT	QQWNYPFT
1E6J-H1	YTFTSYTMH	SYTMH	GYTFTSY	GYTFTSYT
1E6J-H2	WIGYINPSSGYSNY	YINPSSGYSNYNQKFKD	NPSSGY	INPSSGYS
1E6J-H3	RPVVRLGYNFDY	PVVRLGYNFDY	PVVRLGYNFDY	SRPVVRLGYNFDY
1EGJ-L1	ESVYSYGDSFMH	RANESVYSYGDSFMH	RANESVYSYGDSFMH	ESVYSYGDSF
1EGJ-L2	LLIYLASNLAS	LASNLAS	LASNLAS	LAS
1EGJ-L3	QQNNEDPW	QQNNEDPWT	QQNNEDPWT	QQNNEDPWT
1EGJ-H1	YTFTDYYMK	DYYMK	GYTFTDY	GYTFTDYY
1EGJ-H2	WIGDINPSNGGTLY	DINPSNGGTLYNQKFKG	NPSNGG	INPSNGGT
1EGJ-H3	RGDGIHGGFAY	GDGIHGGFAY	GDGIHGGFAY	SRGDGIHGGFAY
1EO8-L1	SDISYMH	SASSDISYMH	SASSDISYMH	SDISY
1EO8-L2	IWIYDTSKLAS	DTSKLAS	DTSKLAS	DTS
1EO8-L3	HQRSSYP	HQRSSYPT	HQRSSYPT	HQRSSYPT
1EO8-H1	YSFSTYFIE	TYFIE	GYSFSTY	GYSFSTYF
1EO8-H2	WIGEILPGSDNTNF	EILPGSDNTNFNEKFKD	LPGSDN	ILPGSDNT
1EO8-H3	RPTGRLWFSY	PTGRLWFSY	PTGRLWFSY	ARPTGRLWFSY
1F58-L1	QGVDFDGASFMN	KASQGVDFDGASFMN	KASQGVDFDGASFMN	QGVDFDGASF
1F58-L2	LLIFAASTLES	AASTLES	AASTLES	AAS
1F58-L3	QQSHEDPL	QQSHEDPLT	QQSHEDPLT	QQSHEDPLT
1F58-H1	YSITSGYSWH	SGYSWH	GYSITSGY	GYSITSGY
1F58-H2	WMGYIHYSAGTNY	YIHYSAGTNYNPSLKS	HYSAG	IHYSAGT
1F58-H3	REEAMPYGNQAYYYAMDC	EEAMPYGNQAYYYAMDC	EEAMPYGNQAYYYAMDC	AREEAMPYGNQAYYYAMDC
1F90-L1	QSLLYSNGKTYLN	ESSQSLLYSNGKTYLN	ESSQSLLYSNGKTYLN	QSLLYSNGKTY
1F90-L2	RLIYLVSKLDS	LVSKLDS	LVSKLDS	LVS
1F90-L3	VQGTHFPR	VQGTHFPRT	VQGTHFPRT	VQGTHFPRT
1F90-H1	YSITSDYAWN	SDYAWN	GYSITSDY	GYSITSDY
1F90-H2	WMGYITYSGSTGY	YITYSGSTGYNPSLKS	TYSGS	ITYSGST
1F90-H3	SYDDYTWFTY	YDDYTWFTY	YDDYTWFTY	ASYDDYTWFTY
1FBI-L1	QDISNYLN	RASQDISNYLN	RASQDISNYLN	QDISNY
1FBI-L2	LLIYYTSRLHS	YTSRLHS	YTSRLHS	YTS
1FBI-L3	QQGYTLPY	QQGYTLPYT	QQGYTLPYT	QQGYTLPYT
1FBI-H1	YTFTSYWMH	SYWMH	GYTFTSY	GYTFTSYW
1FBI-H2	WIGEIDPSDSYPNY	EIDPSDSYPNYNEKFKG	DPSDSY	IDPSDSYP
1FBI-H3	SLYYYGTSYGVLDY	LYYYGTSYGVLDY	LYYYGTSYGVLDY	ASLYYYGTSYGVLDY
1FE8-L1	QDIGNYLN	RASQDIGNYLN	RASQDIGNYLN	QDIGNY

**1FE8-L2**	LLI**YY**TS**R**LHS	**Y**TS**R**LHS	**Y**TS**R**LHS	**Y**TS
**1FE8-L3**	QN**GGTN**P**W**	QN**GGTN**P**W**T	QN**GGTN**P**W**T	QN**GGTN**P**W**T
**1FE8-H1**	FSL**TTYG**VS	**TYG**VS	GFSL**TTY**	GFSL**TTYG**
**1FE8-H2**	WLGVI**WGDGN**T**T**Y	VI**WGDGN**T**T**YHSALIS	**WGDGN**	I**WGDGN**T
**1FE8-H3**	G**NYYGMD**Y	**NYYGMD**Y	**NYYGMD**Y	AG**NYYGMD**Y
**1FJ1-L1**	QDI**N**K**Y**IA	KASQDI**N**K**Y**IA	KASQDI**N**K**Y**IA	QDI**N**K**Y**
**1FJ1-L2**	**LL**I**HY**TS**TLQP**	**Y**TS**TLQP**	**Y**TS**TLQP**	**Y**TS
**1FJ1-L3**	LQ**YD**N**L**Q**R**	LQ**YD**N**L**Q**R**T	LQ**YD**N**L**Q**R**T	LQ**YD**N**L**Q**R**T
**1FJ1-H1**	YT**FTDYSMY**	**DYSMY**	GYT**FTDY**	GYT**FTDYS**
**1FJ1-H2**	RMG**WINTET**GEPTY	**WINTET**GEPTYADDFKG	**NTET**GE	**INTET**GEP
**1FJ1-H3**	**RGLDS**	**GLDS**	**GLDS**	A**RGLDS**
**1FNS-L1**	QDI**N**K**Y**LN	SASQDI**N**K**Y**LN	SASQDI**N**K**Y**LN	QDI**N**K**Y**
**1FNS-L2**	LLIFYTSSLHS	YTSSLHS	YTSSLHS	YTS
**1FNS-L3**	QQ**YEKL**P**W**	QQ**YEKL**P**W**T	QQ**YEKL**P**W**T	QQ**YEKL**P**W**T
**1FNS-H1**	FSL**T**DYGVD	DYGVD	GFSL**T**DY	GFSL**T**DYG
**1FNS-H2**	**W**LG**M**I**WGDGSTD**Y	**M**I**WGDGSTD**YNSALKS	**WGDGS**	I**WGDGST**
**1FNS-H3**	R**D**P**ADYGNY**D**Y**ALDY	**D**P**ADYGNY**D**Y**ALDY	**D**P**ADYGNY**D**Y**ALDY	VR**D**P**ADYGNY**D**Y**ALDY
**1FPT-L1**	QSLV**HSN**G**K**T**Y**LH	SSSQSLV**HSN**G**K**T**Y**LH	SSSQSLV**HSN**G**K**T**Y**LH	QSLV**HSN**G**K**T**Y**
**1FPT-L2**	LLIY**K**VSNRFS	**K**VSNRFS	**K**VSNRFS	**K**VS
**1FPT-L3**	SQ**ST**H**V**P**Y**	SQ**ST**H**V**P**Y**T	SQ**ST**H**V**P**Y**T	SQ**ST**H**V**P**Y**T
**1FPT-H1**	YAFTNY**L**I**Q**	NY**L**I**Q**	GYAFTNY	GYAFTNY**L**
**1FPT-H2**	WIG**VIN**PG**SGGTD**Y	**VIN**PG**SGGTD**YNANFKG	**N**PG**SGG**	**IN**PG**SGGT**
**1FPT-H3**	R**DFYDYDV**GFDY	**DFYDYDV**GFDY	**DFYDYDV**GFDY	AR**DFYDYDV**GFDY
**1FRG-L1**	QSLF**N**SGKRKN**F**LT	KSSQSLF**N**SGKRKN**F**LT	KSSQSLF**N**SGKRKN**F**LT	QSLF**N**SGKRKN**F**
**1FRG-L2**	LLIYWASTRES	WASTRES	WASTRES	WAS
**1FRG-L3**	QN**DYSH**P**L**	QN**DYSH**P**L**T	QN**DYSH**P**L**T	QN**DYSH**P**L**T
**1FRG-H1**	FTFS**S**F**G**M**S**	**S**F**G**M**S**	GFTFS**S**F	GFTFS**S**F**G**
**1FRG-H2**	WVA**TISNGGGY**T**Y**Y	**TISNGGGY**T**Y**YQDSVKG	**SNGGGY**	**ISNGGGY**T
**1FRG-H3**	R**RER**Y**DEKGF**AY	**RER**Y**DEKGF**AY	**RER**Y**DEKGF**AY	AR**RER**Y**DEKGF**AY
**1FSK-L1**	ENV**DTYVF**	KASENV**DTYVF**	KASENV**DTYVF**	ENV**DTY**
**1FSK-L2**	LLLY**G**PSNRYT	**G**PSNRYT	**G**PSNRYT	**G**PS
**1FSK-L3**	G**QSYSY**P**Y**	G**QSYSY**P**Y**T	G**QSYSY**P**Y**T	G**QSYSY**P**Y**T
**1FSK-H1**	YTFT**SYWIN**	**SYWIN**	GYTFT**SY**	GYTFT**SYW**
**1FSK-H2**	WVG**N**I**FPSD**S**Y**T**N**Y	**N**I**FPSD**S**Y**T**N**YNQKFKD	**FPSD**S**Y**	I**FPSD**S**Y**T
**1FSK-H3**	**RGARDTWF**AY	**GARDTWF**AY	**GARDTWF**AY	**TRGARDTWF**AY
**1G9N-L1**	ESVSSDLA	RASESVSSDLA	RASESVSSDLA	ESVSSD
**1G9N-L2**	LLIYGASTRAT	GASTRAT	GASTRAT	GAS
**1G9N-L3**	QQYNN**WPP**RY	QQYNN**WPP**RYT	QQYNN**WPP**RYT	QQYNN**WPP**RYT
**1G9N-H1**	DTFIRYSFT	RYSFT	GDTFIRY	GDTFIRYS
**1G9N-H2**	WMGRI**I**T**ILDV**A**H**Y	RI**I**T**ILDV**A**H**YAPHLQG	**I**T**ILDV**	I**I**T**ILDV**A
**1G9N-H3**	GVYEG**EA**D**EGEYRN**NGFLKH	VYEG**EA**D**EGEYRN**NGFLKH	VYEG**EA**D**EGEYRN**NGFLKH	AGVYEG**EA**D**EGEYRN**NGFLKH
**1GGI-L1**	ESVD**D**DGNSFL**H**	RASESVD**D**DGNSFL**H**	RASESVD**D**DGNSFL**H**	ESVD**D**DGNSF
**1GGI-L2**	**LLIYR**SSNL**I**S	**R**SSNL**I**S	**R**SSNL**I**S	**R**SS
**1GGI-L3**	**QQSN**E**D**P**L**	**QQSN**E**D**P**L**T	**QQSN**E**D**P**L**T	**QQSN**E**D**P**L**T
**1GGI-H1**	FSLSTYGMGV**S**	TYGMGV**S**	GFSLSTYGM	GFSLSTYG
**1GGI-H2**	**W**LA**H**I**FWD**G**D**K**R**Y	**H**I**FWD**G**D**K**R**YNPSLKS	**FWD**G**D**	I**FWD**G**D**K
**1GGI-H3**	**QEGYI**Y	**EGYI**Y	**EGYI**Y	V**QEGYI**Y
**1H0D-L1**	ESV**DNY**GIS**F**MS	RASESV**DNY**GIS**F**MS	RASESV**DNY**GIS**F**MS	ESV**DNY**GIS**F**
**1H0D-L2**	LLIYAASNQGS	AASNQGS	AASNQGS	AAS
**1H0D-L3**	QQ**SKEV**P**L**	QQ**SKEV**P**L**T	QQ**SKEV**P**L**T	QQ**SKEV**P**L**T

1H0D-H1	FTFSSYTMS	SYTMS	GFTFSSY	GFTFSSYT
1H0D-H2	WVATISSGGGNTYY	TISSGGGNTYYPDSVKG	SSGGGN	ISSGGGNT
1H0D-H3	RLGDYGYAYTMDY	LGDYGYAYTMDY	LGDYGYAYTMDY	TRLGDYGYAYTMDY
1HH9-L1	QDINSFLT	KASQDINSFLT	KASQDINSFLT	QDINSF
1HH9-L2	TLIYRANRLMI	RANRLMI	RANRLMI	RAN
1HH9-L3	LQYDDFPL	LQYDDFPLT	LQYDDFPLT	LQYDDFPLT
1HH9-H1	YIFTDYEIH	DYEIH	GYIFTDY	GYIFTDYE
1HH9-H2	WIGGIHPGSSGTAY	GIHPGSSGTAYNQKFKG	HPGSSG	IHPGSSGT
1HH9-H3	RKDY	KDY	KDY	TRKDY
1HI6-L1	QDINSFLT	KASQDINSFLT	KASQDINSFLT	QDINSF
1HI6-L2	TLIYRANRLMI	RANRLMI	RANRLMI	RAN
1HI6-L3	LQYDDFPL	LQYDDFPLT	LQYDDFPLT	LQYDDFPLT
1HI6-H1	YIFTDYEIH	DYEIH	GYIFTDY	GYIFTDYE
1HI6-H2	WIGGIHPGSSGTAY	GIHPGSSGTAYNQKFKG	HPGSSG	IHPGSSGT
1HI6-H3	RKDY	KDY	KDY	TRKDY
1HIM-L1	QSLFNSGKQKNYLT	TSSQSLFNSGKQKNYLT	TSSQSLFNSGKQKNYLT	QSLFNSGKQKNY
1HIM-L2	VLIYWASTRES	WASTRES	WASTRES	WAS
1HIM-L3	QNDYSNPL	QNDYSNPLT	QNDYSNPLT	QNDYSNPLT
1HIM-H1	FSFSSYGMS	SYGMS	GFSFSSY	GFSFSSYG
1HIM-H2	WVATISNGGGYTYY	TISNGGGYTYYPDSVKG	SNGGGY	ISNGGGYT
1HIM-H3	RRERYDENGFAY	RERYDENGFAY	RERYDENGFAY	ARRERYDENGFAY
1HYS-L1	QDISSYLN	SASQDISSYLN	SASQDISSYLN	QDISSY
1HYS-L2	LLIYYTSSLHS	YTSSLHS	YTSSLHS	YTS
1HYS-L3	QQYSKFPW	QQYSKFPWT	QQYSKFPWT	QQYSKFPWT
1HYS-H1	FSLSTSGIGVT	TSGIGVT	GFSLSTSGI	GFSLSTSG
1HYS-H2	WLATIWWDDDNRY	TIWWDDDNRYNPSLKS	WWDDD	IWWDDDN
1HYS-H3	QSAITSVTDSAMDH	SAITSVTDSAMDH	SAITSVTDSAMDH	AQSAITSVTDSAMDH
1I8K-L1	TDIDDDMN	MTSTDIDDDMN	MTSTDIDDDMN	TDIDDD
1I8K-L2	FLISEGNTLRP	EGNTLRP	EGNTLRP	EGN
1I8K-L3	LQSFNVPL	LQSFNVPLT	LQSFNVPLT	LQSFNVPLT
1I8K-H1	FTFRKFGMS	KFGMS	GFTFRKF	GFTFRKFG
1I8K-H2	WVASISTGGYNTYY	SISTGGYNTYYSDNVKG	STGGYN	ISTGGYNT
1I8K-H3	RGYSSTSYAMDY	GYSSTSYAMDY	GYSSTSYAMDY	TRGYSSTSYAMDY
1I9R-L1	QRVSSSTYSYMH	RASQRVSSSTYSYMH	RASQRVSSSTYSYMH	QRVSSSTYSY
1I9R-L2	LLIKYASNLES	YASNLES	YASNLES	YAS
1I9R-L3	QHSWEIPP	QHSWEIPPT	QHSWEIPPT	QHSWEIPPT
1I9R-H1	YIFTSYYMY	SYYMY	GYIFTSY	GYIFTSYY
1I9R-H2	WIGEINPSNGDTNF	EINPSNGDTNFNEKFKS	NPSNGD	INPSNGDT
1I9R-H3	RSDGRNDMDS	SDGRNDMDS	SDGRNDMDS	TRSDGRNDMDS
1IGC-L1	ENVVTYVS	KASENVVTYVS	KASENVVTYVS	ENVVTY
1IGC-L2	LLIYGASNRYT	GASNRYT	GASNRYT	GAS
1IGC-L3	GQGNSYPY	GQGNSYPYT	GQGNSYPYT	GQGNSYPYT
1IGC-H1	FTFSSFGMH	SFGMH	GFTFSSF	GFTFSSFG
1IGC-H2	WVAYISSGSSTLHY	YISSGSSTLHYADTVKG	SSGSST	ISSGSSTL
1IGC-H3	RWGNYPYYAMDY	WGNYPYYAMDY	WGNYPYYAMDY	ARWGNYPYYAMDY
1IQD-L1	QSFSSSYLA	RASQSFSSSYLA	RASQSFSSSYLA	QSFSSSY
1IQD-L2	LLIYGASTRAT	GASTRAT	GASTRAT	GAS
1IQD-L3	QKYGTSAI	QKYGTSAIT	QKYGTSAIT	QKYGTSAIT
1IQD-H1	YTLTELPVH	ELPVH	GYTLTEL	GYTLTELP
1IQD-H2	WVGSFDPESGESIY	SFDPESGESIYAREFQG	DPESGE	FDPESGES

1IQD-H3	VPDPDAFDI	PDPDAFDI	PDPDAFDI	AVPDPDAFDI
1J1P-L1	QSIGNNLH	RASQSIGNNLH	RASQSIGNNLH	QSIGNN
1J1P-L2	LLIKYASQSIS	YASQSIS	YASQSIS	YAS
1J1P-L3	QQANSWPY	QQANSWPYT	QQANSWPYT	QQANSWPYT
1J1P-H1	DSITSDYWS	SDYWS	GDSITSD	GDSITSDY
1J1P-H2	YMGYVSYSGSTYY	YVSYSGSTYYNPSLKS	SYSGS	VSYSGST
1J1P-H3	NWDGDY	WDGDY	WDGDY	ANWDGDY
1JHL-L1	KSISKSLA	RASKSISKSLA	RASKSISKSLA	KSISKS
1JHL-L2	LLIYSGSTLQS	SGSTLQS	SGSTLQS	SGS
1JHL-L3	QQHNEYPW	QQHNEYPWT	QQHNEYPWT	QQHNEYPWT
1JHL-H1	YTFISYWIN	SYWIN	GYTFISY	GYTFISYW
1JHL-H2	WIGNIYPSDSYTNY	NIYPSDSYTNYNQKFKD	YPSDSY	IYPSDSYT
1JHL-H3	RDDNYGAMDY	DDNYGAMDY	DDNYGAMDY	TRDDNYGAMDY
1JPS-L1	RDIKSYLN	RASRDIKSYLN	RASRDIKSYLN	RDIKSY
1JPS-L2	VLIYYATSLAE	YATSLAE	YATSLAE	YAT
1JPS-L3	LQHGESPW	LQHGESPWT	LQHGESPWT	LQHGESPWT
1JPS-H1	FNIKEYYMH	EYYMH	GFNIKEY	GFNIKEYY
1JPS-H2	WVGLIDPEQGNTIY	LIDPEQGNTIYDPKFQD	DPEQGN	IDPEQGNT
1JPS-H3	RDTAAYFDY	DTAAYFDY	DTAAYFDY	ARDTAAYFDY
1JRH-L1	EDIYNRLA	KASEDIYNRLA	KASEDIYNRLA	EDIYNR
1JRH-L2	LLISGATSLET	GATSLET	GATSLET	GAT
1JRH-L3	QQYWSTW	QQYWSTWT	QQYWSTWT	QQYWSTWT
1JRH-H1	FSLTTYGMGVG	TYGMGVG	GFSLTTYGM	GFSLTTYG
1JRH-H2	WLAHIWWDDDKYY	HIWWDDDKYYNPSLKS	WWDDD	IWWDDDK
1JRH-H3	RRAPFYGNHAMDY	RAPFYGNHAMDY	RAPFYGNHAMDY	ARRAPFYGNHAMDY
1KB5-L1	KNIYSYLA	RASKNIYSYLA	RASKNIYSYLA	KNIYSY
1KB5-L2	LLVYNAKTLGE	NAKTLGE	NAKTLGE	NAK
1KB5-L3	QHHYGTPY	QHHYGTPYT	QHHYGTPYT	QHHYGTPYT
1KB5-H1	YSFTGYNMN	GYNMN	GYSFTGY	GYSFTGYN
1KB5-H2	WIGNIDPYYGGISY	NIDPYYGGISYNQKFKG	DPYYGG	IDPYYGGI
1KB5-H3	RSRTDLYYFDY	SRTDLYYFDY	SRTDLYYFDY	ARSRTDLYYFDY
1KB9-L1	QDINNFLN	RASQDINNFLN	RASQDINNFLN	QDINNF
1KB9-L2	LLIYYTSRLHA	YTSRLHA	YTSRLHA	YTS
1KB9-L3	QHHIKFPW	QHHIKFPWT	QHHIKFPWT	QHHIKFPWT
1KB9-H1	YSITSGYYWN	SGYYWN	GYSITSGY	GYSITSGY
1KB9-H2	WVGYISNVGDNNY	YISNVGDNNYNPSLKD	SNVGD	ISNVGDN
1KB9-H3	RSEYYSVTGYAMDY	SEYYSVTGYAMDY	SEYYSVTGYAMDY	ARSEYYSVTGYAMDY
1KC5-L1	ENVDTYVS	KASENVDTYVS	KASENVDTYVS	ENVDTY
1KC5-L2	LLIYGASNRYT	GASNRYT	GASNRYT	GAS
1KC5-L3	GQSYSYPL	GQSYSYPLT	GQSYSYPLT	GQSYSYPLT
1KC5-H1	YSITSDYAWN	SDYAWN	GYSITSDY	GYSITSDY
1KC5-H2	WMAYISYSGSTTY	YISYSGSTTYNPSLKS	SYSGS	ISYSGST
1KC5-H3	RGGTGFDY	GGTGFDY	GGTGFDY	ARGGTGFDY
1KCR-L1	ENVGTYVS	KASENVGTYVS	KASENVGTYVS	ENVGTY
1KCR-L2	LLIYGASNRYT	GASNRYT	GASNRYT	GAS
1KCR-L3	GQTYSYP	GQTYSYPT	GQTYSYPT	GQTYSYPT
1KCR-H1	YSITSDYAWN	SDYAWN	GYSITSDY	GYSITSDY
1KCR-H2	WMGYIRNGGSTTY	YIRNGGSTTYNPSLAS	RNGGS	IRNGGST
1KCR-H3	RGGTGFTY	GGTGFTY	GGTGFTY	ARGGTGFTY
1KCS-L1	ENVGTYVS	KASENVGTYVS	KASENVGTYVS	ENVGTY

**1KCS-L2**	LLIYGASNRYT	GASNRYT	GASNRYT	GAS
**1KCS-L3**	G**QSYSSPL**	G**QSYSSPL**T	G**QSYSSPL**T	G**QSYSSPL**T
**1KCS-H1**	YSITS**DYA**W**N**	S**DYA**W**N**	SYSITS**DY**	SYSITS**DY**
**1KCS-H2**	**W**MG**Y**I**SY**SGST**S**Y	**Y**I**SY**SGST**S**YNPSLKS	**SY**SGS	I**SY**SGST
**1KCS-H3**	**RGGTGF**PY	**GGTGF**PY	**GGTGF**PY	A**RGGTGF**PY
**1KEN-L1**	STIT**SS**FLY	SASSTIT**SS**FLY	SASSTIT**SS**FLY	STIT**SS**F
**1KEN-L2**	LWI**Y**STS**NLAS**	STS**NLAS**	STS**NLAS**	STS
**1KEN-L3**	HQWETFPR	HQWETFPRT	HQWETFPRT	HQWETFPRT
**1KEN-H1**	**Y**SITSG**Y**YWT	SG**Y**YWT	G**Y**SITSG**Y**	G**Y**SITSG**Y**
**1KEN-H2**	WMGYISYDGSNNYN	YISYDGSNNYNPSLKN	SYDGS	ISYDGSN
**1KEN-H3**	AF**YYDYDFFF**D**Y**	F**YYDYDFFF**D**Y**	F**YYDYDFFF**D**Y**	AAF**YYDYDFFF**D**Y**
**1KTR-L1**	QSIV**HSN**G**N**T**Y**L**E**	RSSQSIV**HSN**G**N**T**Y**L**E**	RSSQSIV**HSN**G**N**T**Y**L**E**	QSIV**HSN**G**N**T**Y**
**1KTR-L2**	LLIYKVSNRFS	KVSNRFS	KVSNRFS	KVS
**1KTR-L3**	**FQGSHV**P**F**	**FQGSHV**P**F**T	**FQGSHV**P**F**T	**FQGSHV**P**F**T
**1KTR-H1**	YTFTDY**Y**M**N**	DY**Y**M**N**	GYTFTDY	GYTFTDY**Y**
**1KTR-H2**	**W**IG**D**INPNNGGT**S**Y	**D**INPNNGGT**S**YNQKFKG	NPNNGG	INPNNGGT
**1KTR-H3**	S**QSGA**Y	**QSGA**Y	**QSGA**Y	**E**S**QSGA**Y
**1LK3-L1**	ESVTS**R**M**H**	KASESVTS**R**M**H**	KASESVTS**R**M**H**	ESVTS**R**
**1LK3-L2**	LLIY**K**ASNLAS	**K**ASNLAS	**K**ASNLAS	**K**AS
**1LK3-L3**	QQ**SWNGPL**	QQ**SWNGPL**T	QQ**SWNGPL**T	QQ**SWNGPL**T
**1LK3-H1**	YTFTDF**Y**I**H**	DF**Y**I**H**	GYTFTDF	GYTFTDF**Y**
**1LK3-H2**	**W**IG**Y**I**N**P**NSGYTNY**	**Y**I**N**P**NSGYTNY**NEKFKN	**N**P**NSGY**	I**N**P**NSGYT**
**1LK3-H3**	RG**VPGNNW**FPY	G**VPGNNW**FPY	G**VPGNNW**FPY	TRG**VPGNNW**FPY
**1MHP-L1**	S**SVNH**MF	SASS**SVNH**MF	SASS**SVNH**MF	S**SVNH**
**1MHP-L2**	PWIY**L**TSNLAS	**L**TSNLAS	**L**TSNLAS	**L**TS
**1MHP-L3**	QQ**WSGNPW**	QQ**WSGNPW**T	QQ**WSGNPW**T	QQ**WSGNPW**T
**1MHP-H1**	FTF**SRYT**MS	**RYT**MS	GFTF**SRY**	GFTF**SRYT**
**1MHP-H2**	**W**VA**TISGG**G**H**T**YY**	**TISGG**G**H**T**YY**LDSVKG	**SGG**G**H**	**ISGG**G**H**T
**1MHP-H3**	RGF**GDGG**YFDV	GF**GDGG**YFDV	GF**GDGG**YFDV	TRGF**GDGG**YFDV
**1MLC-L1**	Q**S**ISN**N**LH	RASQ**S**ISN**N**LH	RASQ**S**ISN**N**LH	Q**S**ISN**N**
**1MLC-L2**	LLIK**Y**VSQSSS	**Y**VSQSSS	**Y**VSQSSS	**Y**VS
**1MLC-L3**	QQ**SNSW**P**R**	QQ**SNSW**P**R**T	QQ**SNSW**P**R**T	QQ**SNSW**P**R**T
**1MLC-H1**	YTF**STYW**I**E**	**TYW**I**E**	GYTF**STY**	GYTF**STYW**
**1MLC-H2**	**W**IG**E**I**L**PG**SGSTY**Y	**E**I**L**PG**SGSTY**YNEKFKG	**L**PG**SGS**	I**L**PG**SGST**
**1MLC-H3**	R**GDG**NYGY	**GDG**NYGY	**GDG**NYGY	AR**GDG**NYGY
**1MPA-L1**	QALV**HSN**G**N**T**Y**LH	RSSQALV**HSN**G**N**T**Y**LH	RSSQALV**HSN**G**N**T**Y**LH	QALV**HSN**G**N**T**Y**
**1MPA-L2**	LLIYKVSNRFS	KVSNRFS	KVSNRFS	KVS
**1MPA-L3**	S**QSTHVPR**	S**QSTHVPR**T	S**QSTHVPR**T	S**QSTHVPR**T
**1MPA-H1**	YSF**TSYWLH**	**SYWLH**	GYSF**TSY**	GYSF**TSYW**
**1MPA-H2**	**W**IG**G**I**Y**PGNRDT**R**Y	**G**I**Y**PGNRDT**R**YTQRFKD	**Y**PGNRD	I**Y**PGNRDT
**1MPA-H3**	**IIYFD**YADFIMDY	**IYFD**YADFIMDY	**IYFD**YADFIMDY	S**IIYFD**YADFIMDY
**1N0X-L1**	**HSIRSRR**VA	RSS**HSIRSRR**VA	RSS**HSIRSRR**VA	**HSIRSRR**
**1N0X-L2**	LVIHGVSNRAS	GVSNRAS	GVSNRAS	GVS
**1N0X-L3**	Q**VYGASS**Y	Q**VYGASS**YT	Q**VYGASS**YT	Q**VYGASS**YT
**1N0X-H1**	YRFSNFVIH	NFVIH	GYRFSNF	GYRFSNFV
**1N0X-H2**	WMG**W**INPYNG**NKE**F	**W**INPYNG**NKE**FSAKFQD	NPYNG**N**	INPYNG**NK**
**1N0X-H3**	RVGPYSWDD**SPQ**DNYYMDV	VGPYSWDD**SPQ**DNYYMDV	VGPYSWDD**SPQ**DNYYMDV	ARVGPYSWDD**SPQ**DNYYMDV
**1N64-L1**	QSLL**N**S**RT**RKN**Y**LA	KSSQSLL**N**S**RT**RKN**Y**LA	KSSQSLL**N**S**RT**RKN**Y**LA	QSLL**N**S**RT**RKN**Y**
**1N64-L2**	VLIYWASTRES	WASTRES	WASTRES	WAS
**1N64-L3**	KQ**AYIP**P**L**	KQ**AYIP**P**L**T	KQ**AYIP**P**L**T	KQ**AYIP**P**L**T

**1N64-H1**	YT**FTDFSMH**	**DFSMH**	GYT**FTDF**	GYT**FTDFS**
**1N64-H2**	**W**MG**WVNTET**GEPTY	**WVNTET**GEPTYADDFKG	**NTET**GE	**VNTET**GEP
**1N64-H3**	R**FLLRQ**YFDV	**FLLRQ**YFDV	**FLLRQ**YFDV	AR**FLLRQ**YFDV
**1N6Q-L1**	QDISS**Y**LN	SASQDISS**Y**LN	SASQDISS**Y**LN	QDISS**Y**
**1N6Q-L2**	LLIY**Y**TSSLHS	**Y**TSSLHS	**Y**TSSLHS	**Y**TS
**1N6Q-L3**	QQ**YSKFPW**	QQ**YSKFPW**T	QQ**YSKFPW**T	QQ**YSKFPW**T
**1N6Q-H1**	FSL**STSG**IGVT	**TSG**IGVT	GFSL**STSG**I	GFSL**STSG**
**1N6Q-H2**	WLATI**WWD**D**D**N**R**Y	TI**WWD**D**D**N**R**YNPSLKS	**WWD**D**D**	I**WWD**D**D**N
**1N6Q-H3**	QSA**ITSVTDS**AMDH	SA**ITSVTDS**AMDH	SA**ITSVTDS**AMDH	AQSA**ITSVTDS**AMDH
**1N8Z-L1**	Q**DVNTA**VA	RASQ**DVNTA**VA	RASQ**DVNTA**VA	Q**DVNTA**
**1N8Z-L2**	LLI**YS**AS**F**LYS	**S**AS**F**LYS	**S**AS**F**LYS	**S**AS
**1N8Z-L3**	QQ**HYTT**PP	QQ**HYTT**PPT	QQ**HYTT**PPT	QQ**HYTT**PPT
**1N8Z-H1**	FNIKDT**Y**IH	DT**Y**IH	GFNIKDT	GFNIKDT**Y**
**1N8Z-H2**	WVA**R**I**Y**PT**N**G**YTR**Y	**R**I**Y**PT**N**G**YTR**YADSVKG	**Y**PT**N**G**Y**	I**Y**PT**N**G**YT**
**1N8Z-H3**	R**W**G**GDGFY**AMDY	**W**G**GDGFY**AMDY	**W**G**GDGFY**AMDY	SR**W**G**GDGFY**AMDY
**1NAK-L1**	QSLGHSSGNT**Y**L**H**	RSSQSLGHSSGNT**Y**L**H**	RSSQSLGHSSGNT**Y**L**H**	QSLGHSSGNT**Y**
**1NAK-L2**	**L**LIY**K**VSNR**F**S	**K**VSNR**F**S	**K**VSNR**F**S	**K**VS
**1NAK-L3**	**F**Q**TT**HDP**Y**	**F**Q**TT**HDP**Y**T	**F**Q**TT**HDP**Y**T	**F**Q**TT**HDP**Y**T
**1NAK-H1**	VSIT**SGYWN**	**SGYWN**	GVSIT**SG**	GVSIT**SGY**
**1NAK-H2**	**Y**M**GYI**SKSGSAYY	**YI**SKSGSAYYNPSLKS	SKSGS	**I**SKSGSA
**1NAK-H3**	**IDDFD**I	**DDFD**I	**DDFD**I	A**IDDFD**I
**1NCD-L1**	QDV**ST**AVV	KASQDV**ST**AVV	KASQDV**ST**AVV	QDV**ST**A
**1NCD-L2**	LLI**YW**A**STRHI**	**W**A**STRHI**	**W**A**STRHI**	**W**A**S**
**1NCD-L3**	QQ**HYSP**P**W**	QQ**HYSP**P**W**T	QQ**HYSP**P**W**T	QQ**HYSP**P**W**T
**1NCD-H1**	YTF**TNYG**MN	**NYG**MN	GYTF**TNY**	GYTF**TNYG**
**1NCD-H2**	WMG**WINTNT**G**E**PTY	**WINTNT**G**E**PTYG**E**EF**K**G	**NTNT**G**E**	**INTNT**G**E**P
**1NCD-H3**	R**GEDNF**G**SL**S**D**Y	**GEDNF**G**SL**S**D**Y	**GEDNF**G**SL**S**D**Y	AR**GEDNF**G**SL**S**D**Y
**1NDG-L1**	Q**SISNN**LH	RASQ**SISNN**LH	RASQ**SISNN**LH	Q**SISNN**
**1NDG-L2**	LLI**KY**AS**Q**SIS	**Y**AS**Q**SIS	**Y**AS**Q**SIS	**Y**AS
**1NDG-L3**	QQ**SNSW**P**Y**	QQ**SNSW**P**Y**T	QQ**SNSW**P**Y**T	QQ**SNSW**P**Y**T
**1NDG-H1**	**D**S**IIRDY**WS	**RDY**WS	G**D**S**IIRD**	G**D**S**IIRDY**
**1NDG-H2**	YMG**Y**I**SFSGN**T**F**Y	**Y**I**SFSGN**T**F**YHPSLKS	**SFSGN**	I**SFSGN**T
**1NDG-H3**	**NWD**G**TY**	**WD**G**TY**	**WD**G**TY**	A**NWD**G**TY**
**1NDM-L1**	Q**SISNN**LH	RASQ**SISNN**LH	RASQ**SISNN**LH	Q**SISNN**
**1NDM-L2**	LLI**KY**AS**Q**SIS	**Y**AS**Q**SIS	**Y**AS**Q**SIS	**Y**AS
**1NDM-L3**	QQ**SNSW**P**Y**	QQ**SNSW**P**Y**T	QQ**SNSW**P**Y**T	QQ**SNSW**P**Y**T
**1NDM-H1**	**DS**I**TSDY**WS	**SDY**WS	G**DS**I**TSD**	G**DS**I**TSDY**
**1NDM-H2**	YMG**Y**I**SYSGS**T**Y**Y	**Y**I**SYSGS**T**Y**YHPSLKS	**SYSGS**	I**SYSGS**T
**1NDM-H3**	**RWE**MDY	**WE**MDY	**WE**MDY	A**RWE**MDY
**1NFD-L1**	Q**LPKNFAY**	SGDQ**LPKNFAY**	SGDQ**LPKNFAY**	Q**LPKNF**
**1NFD-L2**	LLI**YMD**N**K**RPS	**MD**N**K**RPS	**MD**N**K**RPS	**MD**N
**1NFD-L3**	L**SS**YGDN**NDL**	L**SS**YGDN**NDL**V	L**SS**YGDN**NDL**V	L**SS**YGDN**NDL**V
**1NFD-H1**	FTFSDF**W**M**Y**	DF**W**M**Y**	GFTFSDF	GFTFSDF**W**
**1NFD-H2**	WVG**R**I**K**NIP**N**NYATEY	**R**I**K**NIP**N**NYATEYADSVRG	**K**NIP**N**NYA	I**K**NIP**N**NYAT
**1NFD-H3**	R**AGRFDHF**DY	**AGRFDHF**DY	**AGRFDHF**DY	TR**AGRFDHF**DY
**1NL0-L1**	TSNIG**NN**YVS	SGSTSNIG**NN**YVS	SGSTSNIG**NN**YVS	TSNIG**NN**Y
**1NL0-L2**	LMIYDVSKRPS	DVSKRPS	DVSKRPS	DVS
**1NL0-L3**	AA**W**D**D**SLSE**F**	AA**W**D**D**SLSE**F**L	AA**W**D**D**SLSE**F**L	AA**W**D**D**SLSE**F**L
**1NL0-H1**	FTF**STYA**M**H**	**TYA**M**H**	GFTF**STY**	GFTF**STYA**
**1NL0-H2**	**W**VA**IISYD**GS**KKY**Y	**IISYD**GS**KKY**YADSVKG	**SYD**GS**K**	**ISYD**GS**KK**

**1NL0-H3**	R**ASIAAAR**VLDY	**ASIAAAR**VLDY	**ASIAAAR**VLDY	AR**ASIAAAR**VLDY
**1NMA-L1**	**QD**I**SNY**LN	RAS**QD**I**SNY**LN	RAS**QD**I**SNY**LN	**QD**I**SNY**
**1NMA-L2**	LLIY**Y**TSNLHS	**Y**TSNLHS	**Y**TSNLHS	**Y**TS
**1NMA-L3**	QQ**DFTL**PF	QQ**DFTL**PFT	QQ**DFTL**PFT	QQ**DFTL**PFT
**1NMA-H1**	YTFTNYNMY	NYNMY	GYTFTNY	GYTFTNYN
**1NMA-H2**	WIGIF**Y**P**GNGDTS**Y	IF**Y**P**GNGDTS**YNQKFKD	**Y**P**GNGD**	F**Y**P**GNGDT**
**1NMA-H3**	RSGG**SYRYD**GGFDY	SGG**SYRYD**GGFDY	SGG**SYRYD**GGFDY	ARSGG**SYRYD**GGFDY
**1NSN-L1**	QSV**STSSF**R**Y**MH	RASQSV**STSSF**R**Y**MH	RASQSV**STSSF**R**Y**MH	QSV**STSSF**R**Y**
**1NSN-L2**	LLI**KY**AS**N**L**E**S	**Y**AS**N**L**E**S	**Y**AS**N**L**E**S	**Y**AS
**1NSN-L3**	QHS**WEIPY**	QHS**WEIPY**T	QHS**WEIPY**T	QHS**WEIPY**T
**1NSN-H1**	**Y**SI**TSD**Y**A**W**N**	**SD**Y**A**W**N**	G**Y**SI**TSD**Y	G**Y**SI**TSD**Y
**1NSN-H2**	WMG**Y**I**TYSGTTS**Y	**Y**I**TYSGTTS**YNPSLKS	**TYSGT**	I**TYSGTT**
**1NSN-H3**	R**GN**GD	**GN**GD	**GN**GD	TR**GN**GD
**1OAZ-L1**	TGAVT**TSNY**AN	RSSTGAVT**TSNY**AN	RSSTGAVT**TSNY**AN	TGAVT**TSNY**
**1OAZ-L2**	GLIGGT**NNRAP**	GT**NNRAP**	GT**NNRAP**	GT**N**
**1OAZ-L3**	ALWY**SN**HL	ALWY**SN**HLV	ALWY**SN**HLV	ALWY**SN**HLV
**1OAZ-H1**	**YT**F**TSYW**MH	**SYW**MH	**GYT**F**TSY**	**GYT**F**TSYW**
**1OAZ-H2**	WIGRI**D**P**N**GGGTKY	RI**D**P**N**GGGTKYNEKFKS	**D**P**N**GGG	I**D**P**N**GGGT
**1OAZ-H3**	**R**M**WYYGTYY**FDY	M**WYYGTYY**FDY	M**WYYGTYY**FDY	A**R**M**WYYGTYY**FDY
**1OB1-L1**	SS**LSSSY**LH	TATSS**LSSSY**LH	TATSS**LSSSY**LH	SS**LSSSY**
**1OB1-L2**	LWIYTTSNLAS	TTSNLAS	TTSNLAS	TTS
**1OB1-L3**	HQ**FHHS**P**Y**	HQ**FHHS**P**Y**T	HQ**FHHS**P**Y**T	HQ**FHHS**P**Y**T
**1OB1-H1**	YT**FTTAGI**Q	**TAGI**Q	GYT**FTTA**	GYT**FTTAG**
**1OB1-H2**	WIG**WINTHS**GVP**Q**Y	**WINTHS**GVP**Q**YADDFKG	**NTHS**GV	**INTHS**GVP
**1OB1-H3**	R**NYYRFDG**GMDF	**NYYRFDG**GMDF	**NYYRFDG**GMDF	AR**NYYRFDG**GMDF
**1ORQ-L1**	SSVSSS**Y**LH	RARSSVSSS**Y**LH	RARSSVSSS**Y**LH	SSVSSS**Y**
**1ORQ-L2**	LWIYSTSNLAS	STSNLAS	STSNLAS	STS
**1ORQ-L3**	QQ**Y**SGNPW	QQ**Y**SGNPWT	QQ**Y**SGNPWT	QQ**Y**SGNPWT
**1ORQ-H1**	YSI**TSLYA**WN	**SLYA**WN	GYSI**TSLY**	GYSI**TSLY**
**1ORQ-H2**	WMG**YINYS**G**Y**TSY	**YINYS**G**Y**TSYNPSLKS	**NYS**G**Y**	**INYS**G**Y**T
**1ORQ-H3**	R**GVDYF**AMDY	**GVDYF**AMDY	**GVDYF**AMDY	TR**GVDYF**AMDY
**1ORS-L1**	SSVSSS**Y**LH	TASSSVSSS**Y**LH	TASSSVSSS**Y**LH	SSVSSS**Y**
**1ORS-L2**	LWIYSTSNLAS	STSNLAS	STSNLAS	STS
**1ORS-L3**	HQ**FH**RSL	HQ**FH**RSLT	HQ**FH**RSLT	HQ**FH**RSLT
**1ORS-H1**	YSI**TNNYA**WN	**NNYA**WN	GYSI**TNNY**	GYSI**TNNY**
**1ORS-H2**	WMG**Y**I**NYS**G**T**TSY	**Y**I**NYS**G**T**TSYNPSLKS	**NYS**G**T**	I**NYS**G**T**T
**1ORS-H3**	R**GYDYF**AMDY	**GYDYF**AMDY	**GYDYF**AMDY	VR**GYDYF**AMDY
**1OSP-L1**	E**D**I**YSRL**A	KASE**D**I**YSRL**A	KASE**D**I**YSRL**A	E**D**I**YSR**
**1OSP-L2**	LLI**SGATSLE**T	**GATSLE**T	**GATSLE**T	**GAT**
**1OSP-L3**	QQ**YW**SPPP	QQ**YW**SPPPT	QQ**YW**SPPPT	QQ**YW**SPPPT
**1OSP-H1**	**E**PIT**SG**FWD	**SG**FWD	G**E**PIT**SG**	G**E**PIT**SG**F
**1OSP-H2**	FMGYIR**Y**GGGTYY	YIR**Y**GGGTYYNPSLKS	R**Y**GGG	IR**Y**GGGT
**1OSP-H3**	**R**S**RDYYGSS**GFAF	S**RDYYGSS**GFAF	S**RDYYGSS**GFAF	A**R**S**RDYYGSS**GFAF
**1OTS-L1**	SSVSYIH	SASSSVSYIH	SASSSVSYIH	SSVSY
**1OTS-L2**	RWI**YD**TS**K**LTS	**D**TS**K**LTS	**D**TS**K**LTS	**D**TS
**1OTS-L3**	QQWSSHPQ	QQWSSHPQT	QQWSSHPQT	QQWSSHPQT
**1OTS-H1**	FDYSRYWMS	RYWMS	GFDYSRY	GFDYSRYW
**1OTS-H2**	WIGEINPVSSTINY	EINPVSSTINYTPSLKD	NPVSST	INPVSSTI
**1OTS-H3**	RL**YYGY**GYW**Y**FDV	L**YYGY**GYW**Y**FDV	L**YYGY**GYW**Y**FDV	ARL**YYGY**GYW**Y**FDV
**1P2C-L1**	**QSI**SN**N**LH	RAS**QSI**SN**N**LH	RAS**QSI**SN**N**LH	**QSI**SN**N**

1P2C-L2	LLIKYTSQSMS	YTSQSMS	YTSQSMS	YTS
1P2C-L3	QQSGSWPR	QQSGSWPRT	QQSGSWPRT	QQSGSWPRT
1P2C-H1	YTFTTYWIE	TYWIE	GYTFTTY	GYTFTTYW
1P2C-H2	WIGEILPGSDSTYY	EILPGSDSTYYNEKVKG	LPGSDS	ILPGSDST
1P2C-H3	RGDGFYVY	GDGFYVY	GDGFYVY	ARGDGFYVY
1P4B-L1	GAVTTSNYASW	RSSTGAVTTSNYAS	RSSTGAVTTSNYAS	TGAVTTSNY
1P4B-L2	LIGGTNNRAPG	GTNNRAP	GTNNRAP	GTN
1P4B-L3	LWYSNHWV	ALWYSNHWV	ALWYSNHWV	ALWYSNHWV
1P4B-H1	FSLTDYGVN	DYGVN	GFSLTDY	GFSLTDYG
1P4B-H2	WLGVIWGDGITDY	VIWGDGITDYNSALKS	WGDGI	IWGDGIT
1P4B-H3	TGLFDY	GLFDY	GLFDY	VTGLFDY
1PKQ-L1	QSLLNSGNQKNYLA	KSSQSLLNSGNQKNYLA	KSSQSLLNSGNQKNYLA	QSLLNSGNQKNY
1PKQ-L2	LLIYGASTRES	GASTRES	GASTRES	GAS
1PKQ-L3	QNDHSYPL	QNDHSYPLT	QNDHSYPLT	QNDHSYPLT
1PKQ-H1	YTFSSFWIE	SFWIE	GYTFSSF	GYTFSSFW
1PKQ-H2	WIGEILPGRGRTNY	EILPGRGRTNYNEKFKG	LPGRGR	ILPGRGRT
1PKQ-H3	TGNTMVNMPY	GNTMVNMPY	GNTMVNMPY	ATGNTMVNMPY
1PZ5-L1	QSLLHSDGNTYLH	RSSQSLLHSDGNTYLH	RSSQSLLHSDGNTYLH	QSLLHSDGNTY
1PZ5-L2	LLIYKVSNRFS	KVSNRFS	KVSNRFS	KVS
1PZ5-L3	SQTTHVP	SQTTHVPT	SQTTHVPT	SQTTHVPT
1PZ5-H1	FTFSNYWME	NYWME	GFTFSNY	GFTFSNYW
1PZ5-H2	WVAEIRLKSNNYATHY	EIRLKSNNYATHYAESVKG	RLKSNNYA	IRLKSNNYAT
1PZ5-H3	RGGAVGAMDY	GGAVGAMDY	GGAVGAMDY	TRGGAVGAMDY
1Q1J-L1	SSNIGNNYVL	SGSSSNIGNNYVL	SGSSSNIGNNYVL	SSNIGNNY
1Q1J-L2	LLIYGNNKRPS	GNNKRPS	GNNKRPS	GNN
1Q1J-L3	ATWDSGLSADW	ATWDSGLSADWV	ATWDSGLSADWV	ATWDSGLSADWV
1Q1J-H1	FTFSDVWLN	DVWLN	GFTFSDV	GFTFSDVW
1Q1J-H2	WVGRIKSRTDGGTTDY	RIKSRTDGGTTDYAASVKG	KSRTDGGT	IKSRTDGGTT
1Q1J-H3	TDGFIMIRGVSEDYYYYY MDV	DGFIMIRGVSEDYYYYYMD V	DGFIMIRGVSEDYYYYY MDV	TTDGFIMIRGVSEDYYY YYMDV
1QFU-L1	QTLVHSNGNTYLH	RSSQTLVHSNGNTYLH	RSSQTLVHSNGNTYLH	QTLVHSNGNTY
1QFU-L2	LLIYKVSNRFS	KVSNRFS	KVSNRFS	KVS
1QFU-L3	SQNTHVPY	SQNTHVPYT	SQNTHVPYT	SQNTHVPYT
1QFU-H1	YTLTTYWMN	TYWMN	GYTLTTY	GYTLTTYW
1QFU-H2	WIGRIDPYDSETHY	RIDPYDSETHYNQKFKD	DPYDSE	IDPYDSET
1QFU-H3	RFLQITTIIYGMDY	FLQITTIIYGMDY	FLQITTIIYGMDY	TRFLQITTIIYGMDY
1QFW-L1	ESVDSYGNSFMQ	RASEDSYGNSFMQ	RASEDSYGNSFMQ	EDSYGNSF
1QFW-L2	LLIYRASNLES	RASNLES	RASNLES	RAS
1QFW-L3	QQSDEYPYMY	QQSDEYPYMYT	QQSDEYPYMYT	QQSDEYPYMYT
1QFW-H1	YTFTSYWMH	SYWMH	DYTFTSY	DYTFTSYW
1QFW-H2	WIGEINPTNGRTYY	EINPTNGRTYYNEK	NPTNGR	INPTNGRT
1QFW-H3	RRYGNSFDY	RYGNSFDY	RYGNSFDY	ARRYGNSFDY
1QKZ-L1	QSLVHSNGNTYLH	RSSQSLVHSNGNTYLH	RSSQSLVHSNGNTYLH	QSLVHSNGNTY
1QKZ-L2	LLIYTVSNRFS	TVSNRFS	TVSNRFS	TVS
1QKZ-L3	SQSTHFP	SQSTHFPT	SQSTHFPT	SQSTHFPT
1QKZ-H1	FTFSDYYMF	DYYMF	GFTFSDY	GFTFSDYY
1QKZ-H2	WVATISDGGAYTYY	TISDGGAYTYYPDSVKG	SDGGAY	ISDGGAYT
1QKZ-H3	RDPLEYYGMDY	DPLEYYGMDY	DPLEYYGMDY	ARDPLEYYGMDY
1RJL-L1	QNINVWLN	HASQNINVWLN	HASQNINVWLN	QNINVW
1RJL-L2	LLIYMASNLHT	MASNLHT	MASNLHT	MAS
1RJL-L3	QQGQSFPL	QQGQSFPLT	QQGQSFPLT	QQGQSFPLT

**1RJL-H1**	F**T**F**TSSW**MH	**SSW**MH	GF**T**F**TSS**	GF**T**F**TSSW**
**1RJL-H2**	WIG**E**I**H**P**N**SGNT**H**Y	**E**I**H**P**N**SGNT**H**YNEKFKG	**H**P**N**SGN	I**H**P**N**SGNT
**1RJL-H3**	RMR**YGD**Y**Y**AMDN	MR**YGD**Y**Y**AMDN	MR**YGD**Y**Y**AMDN	ARMR**YGD**Y**Y**AMDN
**1RZK-L1**	ESVSSDLA	RASESVSSDLA	RASESVSSDLA	ESVSSD
**1RZK-L2**	LLIYGASTRAT	GASTRAT	GASTRAT	GAS
**1RZK-L3**	QQYNN**WPP**RY	QQYNN**WPP**RYT	QQYNN**WPP**RYT	QQYNN**WPP**RYT
**1RZK-H1**	DTFIRYSFT	RYSFT	GDTFIRY	GDTFIRYS
**1RZK-H2**	WMGRI**I**T**IL**D**V**A**H**Y	RI**I**T**IL**D**V**A**H**YAPHLQG	**I**T**IL**DV	I**I**T**IL**D**V**A
**1RZK-H3**	GVYEG**EA**D**EGEYDN**NGFLKH	VYEG**EA**D**EGEYDN**NGFLKH	VYEG**EA**D**EGEYDN**NGFLKH	AGVYEG**EA**D**EGEYDN**NGFLKH
**1S78-L1**	QDVSIGVA	KASQDVSIGVA	KASQDVSIGVA	QDVSIG
**1S78-L2**	**L**LIY**S**AS**Y**R**YT**	**S**AS**Y**R**YT**	**S**AS**Y**R**YT**	**S**AS
**1S78-L3**	QQYYI**Y**PY	QQYYI**Y**PYT	QQYYI**Y**PYT	QQYYI**Y**PYT
**1S78-H1**	F**T**F**TDYT**MD	**DYT**MD	GF**T**F**TDY**	GF**T**F**TDYT**
**1S78-H2**	WVADV**NPNSGGSIY**	DV**NPNSGGSIYNQ**RF**K**G	**NPNSGG**	V**NPNSGGS**
**1S78-H3**	**R**N**LGPS**F**Y**F**DY**	N**LGPS**F**Y**F**DY**	N**LGPS**F**Y**F**DY**	A**R**N**LGPS**F**Y**F**DY**
**1SM3-L1**	TGAVTT**S**N**Y**AN	RSSTGAVTT**S**N**Y**AN	RSSTGAVTT**S**N**Y**AN	TGAVTT**S**N**Y**
**1SM3-L2**	GLIGGTNNR**AP**	GTNNR**AP**	GTNNR**AP**	GTN
**1SM3-L3**	AL**W**Y**SN**H**W**	AL**W**Y**SN**H**W**V	AL**W**Y**SN**H**W**V	AL**W**Y**SN**H**W**V
**1SM3-H1**	**FT**FS**NYW**MN	**NYW**MN	**GFT**FS**NY**	**GFT**FS**NYW**
**1SM3-H2**	WVA**E**I**RL**KSNNYATHY	**E**I**RL**KSNNYATHYAESVKG	**RL**KSNNYA	I**RL**KSNNYAT
**1SM3-H3**	G**VGQ**F**AY**	**VGQ**F**AY**	**VGQ**F**AY**	TG**VGQ**F**AY**
**1SY6-L1**	SSV**SY**MN	SASSSV**SY**MN	SASSSV**SY**MN	SSV**SY**
**1SY6-L2**	RWIY**D**TSKLAS	**D**TSKLAS	**D**TSKLAS	**D**TS
**1SY6-L3**	QQ**WSSN**PF	QQ**WSSN**PFT	QQ**WSSN**PFT	QQ**WSSN**PFT
**1SY6-H1**	YTFT**RYT**M**H**	**RYT**M**H**	GYTFT**RY**	GYTFT**RYT**
**1SY6-H2**	WIG**Y**I**N**P**SR**G**Y**T**N**Y	**Y**I**N**P**SR**G**Y**T**N**YNQKFKD	**N**P**SR**G**Y**	I**N**P**SR**G**Y**T
**1SY6-H3**	R**Y**Y**DD**H**Y**CLDY	**Y**Y**DD**H**Y**CLDY	**Y**Y**DD**H**Y**CLDY	AR**Y**Y**DD**H**Y**CLDY
**1TET-L1**	QSIV**HSSGN**T**Y**FE	KSSQSIV**HSSGN**T**Y**FE	KSSQSIV**HSSGN**T**Y**FE	QSIV**HSSGN**T**Y**
**1TET-L2**	LLIY**K**VSNRFS	**K**VSNRFS	**K**VSNRFS	**K**VS
**1TET-L3**	FQ**GSHI**P**F**	FQ**GSHI**P**F**T	FQ**GSHI**P**F**T	FQ**GSHI**P**F**T
**1TET-H1**	YT**FTTYGMS**	**TYGMS**	GYT**FTTY**	GYT**FTTYG**
**1TET-H2**	**W**MG**WINTYS**GVPTY	**WINTYS**GVPTYADDFKG	**NTYS**GV	**INTYS**GVP
**1TET-H3**	R**RSW**Y**F**DV	**RSW**Y**F**DV	**RSW**Y**F**DV	AR**RSW**Y**F**DV
**1TJI-L1**	**Q**GVTSALA	RA**SQ**GVTSALA	RA**SQ**GVTSALA	**Q**GVTSA
**1TJI-L2**	LLIYDASSLES	DASSLES	DASSLES	DAS
**1TJI-L3**	QQ**LHFYPH**	QQ**LHFYPH**T	QQ**LHFYPH**T	QQ**LHFYPH**T
**1TJI-H1**	FSLSD**FGV**GVG	D**FGV**GVG	GFSLSD**FGV**	GFSLSD**FG**
**1TJI-H2**	WLAII**Y**S**D**D**D**K**R**Y	II**Y**S**D**D**D**K**R**YSPSLNT	**Y**S**D**D**D**	I**Y**S**D**D**D**K
**1TJI-H3**	H**RRGP**TTLFGV**PIARG**P**VN**AMDV	**RRGP**TTLFGV**PIARG**P**VN**AMDV	**RRGP**TTLFGV**PIARG**P**VN**AMDV	AH**RRGP**TTLFGV**PIARG**P**VN**AMDV
**1TPX-L1**	QSLL**D**S**DGKTYLN**	KSSQSLL**D**S**DGKTYLN**	KSSQSLL**D**S**DGKTYLN**	QSLL**D**S**DGKTY**
**1TPX-L2**	**R**LIY**L**VS**RLDS**	**L**VS**RLDS**	**L**VS**RLDS**	**L**VS
**1TPX-L3**	**WQGSHF**P**Q**	**WQGSHF**P**Q**T	**WQGSHF**P**Q**T	**WQGSHF**P**Q**T
**1TPX-H1**	Y**T**F**TNYGMN**	**NYGMN**	GY**T**F**TNY**	GY**T**F**TNYG**
**1TPX-H2**	**W**MG**WINTFT**GEPTY	**WINTFT**GEPTYADDFKG	**NTFT**GE	**INTFT**GEP
**1TPX-H3**	**RGTD**Y	**GTD**Y	**GTD**Y	T**RGTD**Y
**1TZH-L1**	**QASY**SSVA	RAS**QASY**SSVA	RAS**QASY**SSVA	**QASY**SS
**1TZH-L2**	LLIYAASYLYS	AASYLYS	AASYLYS	AAS
**1TZH-L3**	QSSASPA	QSSASPAT	QSSASPAT	QSSASPAT
**1TZH-H1**	FDIYDDDIH	DDDIH	GFDIYDD	GFDIYDDD

**1TZH-H2**	WVAYIAPS**YGYT**DY	YIAPS**YGYT**DYADSVKG	APS**YGY**	IAPS**YGYT**
**1TZH-H3**	RSSDASYSYSAMDY	SSDASYSYSAMDY	SSDASYSYSAMDY	SRSSDASYSYSAMDY
**1TZI-L1**	QS**Y**AYAVA	RASQS**Y**AYAVA	RASQS**Y**AYAVA	QS**Y**AYA
**1TZI-L2**	LLIYDASYLYS	DASYLYS	DASYLYS	DAS
**1TZI-L3**	QQA**Y**SSPD	QQA**Y**SSPDT	QQA**Y**SSPDT	QQA**Y**SSPDT
**1TZI-H1**	FAI**Y**DYDIH	DYDIH	GFAI**Y**DY	GFAI**Y**DYD
**1TZI-H2**	WVADIA**PYAG**ATAY	DIA**PYAG**ATAYADSVKG	A**PYAG**A	IA**PYAG**AT
**1TZI-H3**	RSS**Y**AYYAAMDY	SS**Y**AYYAAMDY	SS**Y**AYYAAMDY	SRSS**Y**AYYAAMDY
**1U8L-L1**	QGVTS**A**LA	RASQGVTS**A**LA	RASQGVTS**A**LA	QGVTS**A**
**1U8L-L2**	LLIYDASSLES	DASSLES	DASSLES	DAS
**1U8L-L3**	Q**QLHFYPH**	Q**QLHFYPH**T	Q**QLHFYPH**T	Q**QLHFYPH**T
**1U8L-H1**	FSLSD**FGV**GVG	D**FGV**GVG	GFSLSD**FGV**	GFSLSD**FG**
**1U8L-H2**	WLAII**YSDDD**K**R**Y	II**YSDDD**K**R**YSPSLNT	**YSDDD**	I**YSDDD**K
**1U8L-H3**	H**RRGP**TTLFGVPIA**R**GP**VN**AMDV	**RRGP**TTLFGVPIA**R**GP**VN**AMDV	**RRGP**TTLFGVPIA**R**GP**VN**AMDV	AH**RRGP**TTLFGVPIA**R**GP**VN**AMDV
**1U8M-L1**	QGVTSALA	RASQGVTSALA	RASQGVTSALA	QGVTSA
**1U8M-L2**	LLIYDASSLES	DASSLES	DASSLES	DAS
**1U8M-L3**	QQ**LHFYPH**	QQ**LHFYPH**T	QQ**LHFYPH**T	QQ**LHFYPH**T
**1U8M-H1**	FSLSD**FGV**GVG	D**FGV**GVG	GFSLSD**FGV**	GFSLSD**FG**
**1U8M-H2**	WLAII**Y**S**D**D**D**K**R**Y	II**Y**S**D**D**D**K**R**YSPSLNT	**Y**S**D**D**D**	I**Y**S**D**D**D**K
**1U8M-H3**	H**RRGP**TTLFGVPIA**R**GP**VN**AMDV	**RRGP**TTLFGVPIA**R**GP**VN**AMDV	**RRGP**TTLFGVPIA**R**GP**VN**AMDV	AH**RRGP**TTLFGVPIA**R**GP**VN**AMDV
**1U8N-L1**	QGVTSALA	RASQGVTSALA	RASQGVTSALA	QGVTSA
**1U8N-L2**	LLIYDASSLES	DASSLES	DASSLES	DAS
**1U8N-L3**	QQ**LHFYPH**	QQ**LHFYPH**T	QQ**LHFYPH**T	QQ**LHFYPH**T
**1U8N-H1**	FSLSD**FGV**GVG	D**FGV**GVG	GFSLSD**FGV**	GFSLSD**FG**
**1U8N-H2**	WLAII**Y**S**D**D**D**K**R**Y	II**Y**S**D**D**D**K**R**YSPSLNT	**Y**S**D**D**D**	I**Y**S**D**D**D**K
**1U8N-H3**	H**RRGP**TTLFGVPIA**R**GP**VN**AMDV	**RRGP**TTLFGVPIA**R**GP**VN**AMDV	**RRGP**TTLFGVPIA**R**GP**VN**AMDV	AH**RRGP**TTLFGVPIA**R**GP**VN**AMDV
**1U8O-L1**	QGV**T**SALA	RASQGV**T**SALA	RASQGV**T**SALA	QGV**T**SA
**1U8O-L2**	LLIYDASSLES	DASSLES	DASSLES	DAS
**1U8O-L3**	QQ**LHFYPH**	QQ**LHFYPH**T	QQ**LHFYPH**T	QQ**LHFYPH**T
**1U8O-H1**	FSLSDF**G**VGVG	DF**G**VGVG	GFSLSDF**G**V	GFSLSDF**G**
**1U8O-H2**	WLAII**Y**S**D**D**D**K**R**Y	II**Y**S**D**D**D**K**R**YSPSLNT	**Y**S**D**D**D**	I**Y**S**D**D**D**K
**1U8O-H3**	H**RRGP**TTLFGVPIA**R**GP**VN**AMDV	**RRGP**TTLFGVPIA**R**GP**VN**AMDV	**RRGP**TTLFGVPIA**R**GP**VN**AMDV	AH**RRGP**TTLFGVPIA**R**GP**VN**AMDV
**1U8P-L1**	QGVTSALA	RASQGVTSALA	RASQGVTSALA	QGVTSA
**1U8P-L2**	LLIYDASSLES	DASSLES	DASSLES	DAS
**1U8P-L3**	QQ**LHFYPH**	QQ**LHFYPH**T	QQ**LHFYPH**T	QQ**LHFYPH**T
**1U8P-H1**	FSLSD**FGV**GVG	D**FGV**GVG	GFSLSD**FGV**	GFSLSD**FG**
**1U8P-H2**	WLAII**Y**S**D**D**D**K**R**Y	II**Y**S**D**D**D**K**R**YSPSLNT	**Y**S**D**D**D**	I**Y**S**D**D**D**K
**1U8P-H3**	H**RRGP**TTLFGVPIA**R**GP**VN**AMDV	**RRGP**TTLFGVPIA**R**GP**VN**AMDV	**RRGP**TTLFGVPIA**R**GP**VN**AMDV	AH**RRGP**TTLFGVPIA**R**GP**VN**AMDV
**1U8Q-L1**	QG**VTSA**LA	RASQG**VTSA**LA	RASQG**VTSA**LA	QG**VTSA**
**1U8Q-L2**	LLIYDASSLES	DASSLES	DASSLES	DAS
**1U8Q-L3**	Q**QLHFYPH**	Q**QLHFYPH**T	Q**QLHFYPH**T	Q**QLHFYPH**T
**1U8Q-H1**	FSLSD**FGV**GVG	D**FGV**GVG	GFSLSD**FGV**	GFSLSD**FG**
**1U8Q-H2**	WLA**I**I**Y**S**D**D**D**K**R**Y	**I**I**Y**S**D**D**D**K**R**YSPSLNT	**Y**S**D**D**D**	I**Y**S**D**D**D**K
**1U8Q-H3**	H**RRGP**TTLFGVPIA**R**GP**VN**AMDV	**RRGP**TTLFGVPIA**R**GP**VN**AMDV	**RRGP**TTLFGVPIA**R**GP**VN**AMDV	AH**RRGP**TTLFGVPIA**R**GP**VN**AMDV
**1U92-L1**	QGVTS**A**LA	RASQGVTS**A**LA	RASQGVTS**A**LA	QGVTS**A**
**1U92-L2**	LLIYDASSLES	DASSLES	DASSLES	DAS

**1U92-L3**	QQ**LHFYPH**	QQ**LHFYPH**T	QQ**LHFYPH**T	QQ**LHFYPH**T
**1U92-H1**	FSLSD**FGV**GVG	D**FGV**GVG	GFSLSD**FGV**	GFSLSD**FG**
**1U92-H2**	WLAII**Y**S**DDD**K**R**Y	II**Y**S**DDD**K**R**YSPSLNT	**Y**S**DDD**	I**Y**S**DDD**K
**1U92-H3**	H**RRGP**TTLFGVPIA**R**GP**V** **N**AMDV	**RRGP**TTLFGVPIA**R**GP**VN**A MDV	**RRGP**TTLFGVPIA**R**GP**V** **N**AMDV	AH**RRGP**TTLFGVPIA**R**G P**VN**AMDV
**1U93-L1**	QGV**T**SALA	RASQGV**T**SALA	RASQGV**T**SALA	QGV**T**SA
**1U93-L2**	LLIYDASSLES	DASSLES	DASSLES	DAS
**1U93-L3**	QQ**LHFYPH**	QQ**LHFYPH**T	QQ**LHFYPH**T	QQ**LHFYPH**T
**1U93-H1**	FSLSD**FGV**GVG	D**FGV**GVG	GFSLSD**FGV**	GFSLSD**FG**
**1U93-H2**	WLAII**Y**S**D**D**D**K**R**Y	II**Y**S**D**D**D**K**R**YSPSLNT	**Y**S**D**D**D**	I**Y**S**D**D**D**K
**1U93-H3**	H**RRGP**TTLFGVPIA**R**GP**V** **N**AMDV	**RRGP**TTLFGVPIA**R**GP**VN**A MDV	**RRGP**TTLFGVPIA**R**GP**V** **N**AMDV	AH**RRGP**TTLFGVPIA**R**G P**VN**AMDV
**1U95-L1**	QGVTSALA	RASQGVTSALA	RASQGVTSALA	QGVTSA
**1U95-L2**	LLIYDASSLES	DASSLES	DASSLES	DAS
**1U95-L3**	QQ**LHFYPH**	QQ**LHFYPH**T	QQ**LHFYPH**T	QQ**LHFYPH**T
**1U95-H1**	FSLSD**FGV**GVG	D**FGV**GVG	GFSLSD**FGV**	GFSLSD**FG**
**1U95-H2**	WLAII**Y**S**D**D**D**K**R**Y	II**Y**S**D**D**D**K**R**YSPSLNT	**Y**S**D**D**D**	I**Y**S**D**D**D**K
**1U95-H3**	H**RRGP**TTLFGVPIA**R**GP**V** **N**AMDV	**RRGP**TTLFGVPIA**R**GP**VN**A MDV	**RRGP**TTLFGVPIA**R**GP**V** **N**AMDV	AH**RRGP**TTLFGVPIA**R**G P**VN**AMDV
**1UAC-L1**	QS**IGNN**LH	RASQS**IGNN**LH	RASQS**IGNN**LH	QS**IGNN**
**1UAC-L2**	LLI**KY**A**SQ**SIS	**Y**A**SQ**SIS	**Y**A**SQ**SIS	**Y**A**S**
**1UAC-L3**	QQ**SNSW**P**Y**	QQ**SNSW**P**Y**T	QQ**SNSW**P**Y**T	QQ**SNSW**P**Y**T
**1UAC-H1**	**D**SI**TSDY**WS	**SDY**WS	G**D**SI**TSD**	G**D**SI**TSDY**
**1UAC-H2**	YMG**Y**V**SSF**G**S**T**F**Y	**Y**V**SSF**G**S**T**F**YNPSLKS	**SSF**G**S**	V**SSF**G**S**T
**1UAC-H3**	**NWD**GDY	**WD**GDY	**WD**GDY	A**NWD**GDY
**1UJ3-L1**	**Q**DI**K**S**F**LS	KAS**Q**DI**K**S**F**LS	KAS**Q**DI**K**S**F**LS	**Q**DI**K**S**F**
**1UJ3-L2**	SLIY**Y**ATSLAD	**Y**ATSLAD	**Y**ATSLAD	**Y**AT
**1UJ3-L3**	LQ**HGESPY**	LQ**HGESPY**T	LQ**HGESPY**T	LQ**HGESPY**T
**1UJ3-H1**	**FNIKDYY**MH	**DYY**MH	G**FNIKDY**	G**FNIKDYY**
**1UJ3-H2**	WIGG**NDPANGHSMY**	G**NDPANGHSMY**DPKF**Q**G	**DPANGH**	**NDPANGHS**
**1UJ3-H3**	R**DSGYA**MDY	**DSGYA**MDY	**DSGYA**MDY	AR**DSGYA**MDY
**1UWX-L1**	QTIGT**W**LA	LASQTIGT**W**LA	LASQTIGT**W**LA	QTIGT**W**
**1UWX-L2**	LLIYAATSLAD	AATSLAD	AATSLAD	AAT
**1UWX-L3**	QQ**LSS**TP**Y**	QQ**LSS**TP**Y**T	QQ**LSS**TP**Y**T	QQ**LSS**TP**Y**T
**1UWX-H1**	YT**FTNFGL**N	**NFGL**N	GYT**FTNF**	GYT**FTNFG**
**1UWX-H2**	WMG**WINTYT**GESTY	**WINTYT**GESTYADDFKG	**NTYT**GE	**INTYT**GES
**1UWX-H3**	R**GFYYYG**SR**YF**YFDY	**GFYYYG**SR**YF**YFDY	**GFYYYG**SR**YF**YFDY	AR**GFYYYG**SR**YF**YFDY
**1V7M-L1**	SSV**SY**M**Y**	SASSSV**SY**M**Y**	SASSSV**SY**M**Y**	SSV**SY**
**1V7M-L2**	**L**WI**YS**TS**N**LAS	**S**TS**N**LAS	**S**TS**N**LAS	**S**TS
**1V7M-L3**	QQ**R**SG**Y**P**R**	QQ**R**SG**Y**P**R**T	QQ**R**SG**Y**P**R**T	QQ**R**SG**Y**P**R**T
**1V7M-H1**	F**T**FS**DAWM**D	**DAWM**D	GF**T**FS**DA**	GF**T**FS**DAW**
**1V7M-H2**	WVA**E**I**RS**K**VN**NHAIHY	**E**I**RS**K**VN**NHAIHYAESVKG	**RS**K**VN**NHA	I**RS**K**VN**NHAI
**1V7M-H3**	**GWS**F**LY**	**WS**F**LY**	**WS**F**LY**	S**GWS**F**LY**
**1VFB-L1**	G**NIHNY**LA	RASG**NIHNY**LA	RASG**NIHNY**LA	G**NIHNY**
**1VFB-L2**	LLV**YY**T**TT**LAD	**Y**T**TT**LAD	**Y**T**TT**LAD	**Y**T**T**
**1VFB-L3**	QH**FWST**P**R**	QH**FWST**P**R**T	QH**FWST**P**R**T	QH**FWST**P**R**T
**1VFB-H1**	F**S**L**TGYG**VN	**GYG**VN	GF**S**L**TGY**	GF**S**L**TGYG**
**1VFB-H2**	WLGMI**WGDG**NT**D**Y	MI**WGDG**NT**D**YNSALKS	**WGDG**N	I**WGDG**NT
**1VFB-H3**	**R**E**RDYR**LDY	E**RDYR**LDY	E**RDYR**LDY	A**R**E**RDYR**LDY
**1W72-L1**	NIGSRSVH	GGNNIGSRSVH	GGNNIGSRSVH	NIGSRS
**1W72-L2**	LVVYDDSDRPS	DDSDRPS	DDSDRPS	DDS

**1W72-L3**	QV**W**D**S**RT**D**HW	QV**W**D**S**RT**D**HWV	QV**W**D**S**RT**D**HWV	QV**W**D**S**RT**D**HWV
**1W72-H1**	FTFDDYAMH	DYAMH	GFTFDDY	GFTFDDYA
**1W72-H2**	WVSGI**SWN**S**GS**IGY	GI**SWN**S**GS**IGYADSVKG	**SWN**S**GS**	I**SWN**S**GS**I
**1W72-H3**	RGRGF**H**Y**Y**YYGMDI	GRGF**H**Y**Y**YYGMDI	GRGF**H**Y**Y**YYGMDI	ARGRGF**H**Y**Y**YYGMDI
**1WEJ-L1**	G**NIHNYL**A	RASG**NIHNYL**A	RASG**NIHNYL**A	G**NIHNY**
**1WEJ-L2**	LLVY**N**A**K**TLAD	**N**A**K**TLAD	**N**A**K**TLAD	**N**A**K**
**1WEJ-L3**	QH**FWS**TP**W**	QH**FWS**TP**W**T	QH**FWS**TP**W**T	QH**FWS**TP**W**T
**1WEJ-H1**	FNI**KDTY**MH	**DTY**MH	GFNI**KDT**	GFNI**KDTY**
**1WEJ-H2**	WIG**R**I**D**PA**S**G**N**T**K**Y	**R**I**D**PA**S**G**N**T**K**YDPKFQD	**D**PA**S**G**N**	I**D**PA**S**G**N**T
**1WEJ-H3**	GY**DYG**NFDY	Y**DYG**NFDY	Y**DYG**NFDY	AGY**DYG**NFDY
**1XCQ-L1**	QSLL**N**S**RTR**KN**Y**LA	KSSQSLL**N**S**RTR**KN**Y**LA	KSSQSLL**N**S**RTR**KN**Y**LA	QSLL**N**S**RTR**KN**Y**
**1XCQ-L2**	VLIY**W**ASTRES	**W**ASTRES	**W**ASTRES	**W**AS
**1XCQ-L3**	KQ**AYIP**P**L**	KQ**AYIP**P**L**T	KQ**AYIP**P**L**T	KQ**AYIP**P**L**T
**1XCQ-H1**	**YT**FT**DF**SM**H**	**DF**SM**H**	**GYT**FT**DF**	**GYT**FT**DF**S
**1XCQ-H2**	**W**MG**W**V**N**TE**T**GEP**T**Y	**W**V**N**TE**T**GEP**T**YADDFKG	**N**TE**T**GE	V**N**TE**T**GEP
**1XCQ-H3**	**RFLLRQ**YF**D**V	**FLLRQ**YF**D**V	**FLLRQ**YF**D**V	A**RFLLRQ**YF**D**V
**1XCT-L1**	QSLL**N**S**RT**RKN**Y**LA	KSSQSLL**N**S**RT**RKN**Y**LA	KSSQSLL**N**S**RT**RKN**Y**LA	QSLL**N**S**RT**RKN**Y**
**1XCT-L2**	VLIY**W**ASTRES	**W**ASTRES	**W**ASTRES	**W**AS
**1XCT-L3**	KQ**AYIP**P**L**	KQ**AYIP**P**L**T	KQ**AYIP**P**L**T	KQ**AYIP**P**L**T
**1XCT-H1**	YTF**TDFSMH**	**DFSMH**	GYTF**TDF**	GYTF**TDFS**
**1XCT-H2**	**W**MG**WVNTET**G**E**P**T**Y	**WVNTET**G**E**P**T**YADDFKG	**NTET**G**E**	**VNTET**G**E**P
**1XCT-H3**	R**FLLRQ**YFDV	**FLLRQ**YFDV	**FLLRQ**YFDV	AR**FLLRQ**YFDV
**1XGU-L1**	Q**SISNN**LH	RASQ**SISNN**LH	RASQ**SISNN**LH	Q**SISNN**
**1XGU-L2**	LLI**KY**AS**Q**SIS	**Y**AS**Q**SIS	**Y**AS**Q**SIS	**Y**AS
**1XGU-L3**	QQ**SNSW**P**Y**	QQ**SNSW**P**Y**T	QQ**SNSW**P**Y**T	QQ**SNSW**P**Y**T
**1XGU-H1**	**D**SV**TSDF**WS	**SDF**WS	G**D**SV**TSD**	G**D**SV**TSDF**
**1XGU-H2**	YMG**Y**I**SYSGS**T**Y**Y	**Y**I**SYSGS**T**Y**YHPSLKS	**SYSGS**	I**SYSGS**T
**1XGU-H3**	S**WG**GDV	**WG**GDV	**WG**GDV	AS**WG**GDV
**1XGY-L1**	KSLL**H**S**N**GIT**Y**L**Y**	RSSKSLL**H**S**N**GIT**Y**L**Y**	RSSKSLL**H**S**N**GIT**Y**L**Y**	KSLL**H**S**N**GIT**Y**
**1XGY-L2**	LLI**YR**MSNLAS	**R**MSNLAS	**R**MSNLAS	**R**MS
**1XGY-L3**	GQ**MLEH**P**L**	GQ**MLEH**P**L**T	GQ**MLEH**P**L**T	GQ**MLEH**P**L**T
**1XGY-H1**	YTFTD**YYIN**	D**YYIN**	GYTFTD**Y**	GYTFTD**YY**
**1XGY-H2**	**W**IG**W**IFPRNGNTKY	**W**IFPRNGNTKYNEKFKG	FPRNGN	IFPRNGNT
**1XGY-H3**	**TTVSYVM**DY	**TVSYVM**DY	**TVSYVM**DY	**ATTVSYVM**DY
**1XIW-L1**	QDI**R**N**Y**LN	RASQDI**R**N**Y**LN	RASQDI**R**N**Y**LN	QDI**R**N**Y**
**1XIW-L2**	LLIY**Y**TSRLHS	**Y**TSRLHS	**Y**TSRLHS	**Y**TS
**1XIW-L3**	QQ**GNTL**P**W**	QQ**GNTL**P**W**T	QQ**GNTL**P**W**T	QQ**GNTL**P**W**T
**1XIW-H1**	Y**S**F**TG**Y**T**MN	**G**Y**T**MN	GY**S**F**TG**Y	GY**S**F**TG**Y**T**
**1XIW-H2**	WMG**L**I**N**P**YK**G**VST**Y	**L**I**N**P**YK**G**VST**YNQKFKD	**N**P**YK**G**V**	I**N**P**YK**G**VS**
**1XIW-H3**	RS**GYYGD**S**DW**YFDV	S**GYYGD**S**DW**YFDV	S**GYYGD**S**DW**YFDV	ARS**GYYGD**S**DW**YFDV
**1YJD-L1**	QN**IY**V**W**LN	HASQN**IY**V**W**LN	HASQN**IY**V**W**LN	QN**IY**V**W**
**1YJD-L2**	LLIY**K**ASNLHT	**K**ASNLHT	**K**ASNLHT	**K**AS
**1YJD-L3**	QQ**GQT**YP**Y**	QQ**GQT**YP**Y**T	QQ**GQT**YP**Y**T	QQ**GQT**YP**Y**T
**1YJD-H1**	Y**T**F**TS**Y**Y**IH	**S**Y**Y**IH	GY**T**F**TS**Y	GY**T**F**TS**Y**Y**
**1YJD-H2**	WIGCI**Y**P**GN**VNTNY	CI**Y**P**GN**VNTNYNEKFKD	**Y**P**GN**VN	I**Y**P**GN**VNT
**1YJD-H3**	RS**HYGLDW**NFDV	S**HYGLDW**NFDV	S**HYGLDW**NFDV	TRS**HYGLDW**NFDV
**1YNT-L1**	QDI**S**N**Y**LN	RASQDI**S**N**Y**LN	RASQDI**S**N**Y**LN	QDI**S**N**Y**
**1YNT-L2**	LLIY**Y**TS**R**LHS	**Y**TS**R**LHS	**Y**TS**R**LHS	**Y**TS
**1YNT-L3**	QQ**GNTL**P**Y**	QQ**GNTL**P**Y**T	QQ**GNTL**P**Y**T	QQ**GNTL**P**Y**T
**1YNT-H1**	YT**FTDYGMH**	**DYGMH**	GYT**FTDY**	GYT**FTDYG**

**1YNT-H2**	WIG**IISTYSGDASY**	**IISTYSGDASY**N**Q**KFKG	**STYSGD**	**ISTYSGDA**
**1YNT-H3**	R**SSTWY**YFDY	**SSTWY**YFDY	**SSTWY**YFDY	AR**SSTWY**YFDY
**1YQV-L1**	SSV**NY**M**Y**	SASSSV**NY**M**Y**	SASSSV**NY**M**Y**	SSV**NY**
**1YQV-L2**	RWIY**D**TSKLAS	**D**TSKLAS	**D**TSKLAS	**D**TS
**1YQV-L3**	Q**QWGRNP**	Q**QWGRNP**T	Q**QWGRNP**T	Q**QWGRNP**T
**1YQV-H1**	YTF**SDYWIE**	**DYWIE**	GYTF**SDY**	GYTF**SDYW**
**1YQV-H2**	**W**IG**E**I**L**PG**SGSTN**Y	**E**I**L**PG**SGSTN**YHERFKG	**L**PG**SGS**	I**L**PG**SGST**
**1YQV-H3**	H**GNYD**FDG	**GNYD**FDG	**GNYD**FDG	LH**GNYD**FDG
**1YY9-L1**	**QS**IGT**N**IH	RA**SQS**IGT**N**IH	RA**SQS**IGT**N**IH	**QS**IGT**N**
**1YY9-L2**	LLI**KY**ASESIS	**Y**ASESIS	**Y**ASESIS	**Y**AS
**1YY9-L3**	QQ**NNNWPT**	QQ**NNNWPT**T	QQ**NNNWPT**T	QQ**NNNWPT**T
**1YY9-H1**	FSLT**N**YGVH	**N**YGVH	GFSLT**N**Y	GFSLT**N**YG
**1YY9-H2**	WLGVI**WSGGNTDY**	VI**WSGGNTDY**NTPFTS	**WSGGN**	I**WSGGNT**
**1YY9-H3**	RAL**TYYDYE**FAY	AL**TYYDYE**FAY	AL**TYYDYE**FAY	ARAL**TYYDYE**FAY
**1Z3G-L1**	SSVS**Y**MN	SASSSVS**Y**MN	SASSSVS**Y**MN	SSVS**Y**
**1Z3G-L2**	RWIYDTSKLAS	DTSKLAS	DTSKLAS	DTS
**1Z3G-L3**	QQ**W**SSNPP	QQ**W**SSNPPT	QQ**W**SSNPPT	QQ**W**SSNPPT
**1Z3G-H1**	Y**T**F**TSYW**M**H**	**SYW**M**H**	GY**T**F**TSY**	GY**T**F**TSYW**
**1Z3G-H2**	WIG**M**I**H**P**HS**G**STN**Y	**M**I**H**P**HS**G**STN**YNEKFKS	**H**P**HS**G**S**	I**H**P**HS**G**ST**
**1Z3G-H3**	**RGWD**VAY	**GWD**VAY	**GWD**VAY	A**RGWD**VAY
**1ZA3-L1**	**QDVNTA**VA	RAS**QDVNTA**VA	RAS**QDVNTA**VA	**QDVNTA**
**1ZA3-L2**	LLIY**A**ASYLYS	**A**ASYLYS	**A**ASYLYS	**A**AS
**1ZA3-L3**	QSS**SSPY**	QSS**SSPY**T	QSS**SSPY**T	QSS**SSPY**T
**1ZA3-H1**	FSIY**S**YSIH	**S**YSIH	GFSIY**S**Y	GFSIY**S**YS
**1ZA3-H2**	WVASISP**YSGYT**SY	SISP**YSGYT**SYA**DSVKG**	SP**YSGY**	ISP**YSGYT**
**1ZA3-H3**	R**Y**S**SYYSYY**YSSSS**Y**S**YA**MDY	**Y**S**SYYSYY**YSSSS**Y**S**Y**AMDY	**Y**S**SYYSYY**YSSSS**Y**S**YA**MDY	SR**Y**S**SYYSYY**YSSSS**Y**S**Y**AMDY
**1ZTX-L1**	QDV**STA**VA	KASQDV**STA**VA	KASQDV**STA**VA	QDV**STA**
**1ZTX-L2**	LLIS**W**ASTRHT	**W**ASTRHT	**W**ASTRHT	**W**AS
**1ZTX-L3**	QQ**HYTT**P**L**	QQ**HYTT**P**L**T	QQ**HYTT**P**L**T	QQ**HYTT**P**L**T
**1ZTX-H1**	**YTF**S**DYWIE**	**DYWIE**	**GYTF**S**DY**	**GYTF**S**DYW**
**1ZTX-H2**	WIGDI**L**CGTG**R**T**R**Y	DI**L**CGTG**R**T**R**YNEKLKA	**L**CGTG**R**	I**L**CGTG**R**T
**1ZTX-H3**	**RSASYGDY**ADY	**SASYGDY**ADY	**SASYGDY**ADY	A**RSASYGDY**ADY
**2A6I-L1**	QDISN**Y**LN	RASQDISN**Y**LN	RASQDISN**Y**LN	QDISN**Y**
**2A6I-L2**	LLI**Y**YTSR**L**HS	YTSR**L**HS	YTSR**L**HS	YTS
**2A6I-L3**	QQGNT**L**P**R**	QQGNT**L**P**R**T	QQGNT**L**P**R**T	QQGNT**L**P**R**T
**2A6I-H1**	YTF**TSYGIN**	**SYGIN**	GYTF**TSY**	GYTF**TSYG**
**2A6I-H2**	**W**IG**YINP**GNGYTKY	**YINP**GNGYTKYNEKFKG	**NP**GNGY	**INP**GNGYT
**2A6I-H3**	**RSVYYGG**S**YYF**DY	**SVYYGG**S**YYF**DY	**SVYYGG**S**YYF**DY	**ARSVYYGG**S**YYF**DY
**2ADF-L1**	QDINK**Y**IA	KASQDINK**Y**IA	KASQDINK**Y**IA	QDINK**Y**
**2ADF-L2**	LLI**HY**TS**T**L**QP**	**Y**TS**T**L**QP**	**Y**TS**T**L**QP**	**Y**TS
**2ADF-L3**	LQ**YDNLR**	LQ**YDNLR**T	LQ**YDNLR**T	LQ**YDNLR**T
**2ADF-H1**	Y**T**F**INYG**M**N**	**NYG**M**N**	GY**T**F**INY**	GY**T**F**INYG**
**2ADF-H2**	**W**MG**W**K**N**T**NT**G**E**TTY	**WKN**T**NT**G**E**TTYGEEFRG	**N**T**NT**G**E**	K**N**T**NT**G**E**T
**2ADF-H3**	**RDNPYY**ALD**Y**	**DNPYY**ALD**Y**	**DNPYY**ALD**Y**	A**RDNPYY**ALD**Y**
**2AEP-L1**	QN**V**G**TN**VA	KASQN**V**G**TN**VA	KASQN**V**G**TN**VA	QN**V**G**TN**
**2AEP-L2**	PLM**YS**A**SYR**Y**S**	**S**A**SYR**Y**S**	**S**A**SYR**Y**S**	**S**A**S**
**2AEP-L3**	QQ**FN**R**Y**PL	QQ**FN**R**Y**PLT	QQ**FN**R**Y**PLT	QQ**FN**R**Y**PLT
**2AEP-H1**	F**T**F**IDY**YMS	**DY**YMS	GF**T**F**IDY**	GF**T**F**IDYY**
**2AEP-H2**	WLG**L**I**RNKGNGY**T**ME**Y	**L**I**RNKGNGY**T**ME**YSASLKG	**RNKGNGY**T	I**RNKGNGY**T**M**
**2AEP-H3**	RV**DYGTN**YDY	V**DYGTN**YDY	V**DYGTN**YDY	ARV**DYGTN**YDY

**2AP2-L1**	SLL**N**SG**N**QKN**Y**LTW	KSSQSLL**N**SG**N**QKN**Y**LT	KSSQSLL**N**SG**N**QKN**Y**LT	QSLL**N**SG**N**QKN**Y**
**2AP2-L2**	LIY**W**ASTRESG	**W**ASTRES	**W**ASTRES	**W**AS
**2AP2-L3**	**NDYSY**P**L**T	Q**NDYSY**P**L**T	Q**NDYSY**P**L**T	Q**NDYSY**P**L**T
**2AP2-H1**	FNIKDD**F**MH	DD**F**MH	GFNIKDD	GFNIKDD**F**
**2AP2-H2**	WIG**R**I**D**PA**N**DNT**K**Y	**R**I**D**PA**N**DNT**K**YAPKFQD	**D**PA**N**DN	I**D**PA**N**DNT
**2AP2-H3**	R**REVYSYYS**PLDV	**REVYSYYS**PLDV	**REVYSYYS**PLDV	AR**REVYSYYS**PLDV
**2ARJ-L1**	QNVR**NN**IA	KASQNVR**NN**IA	KASQNVR**NN**IA	QNVR**NN**
**2ARJ-L2**	LLI**YY**AS**Y**RYT	**Y**AS**Y**RYT	**Y**AS**Y**RYT	**Y**AS
**2ARJ-L3**	QR**IYNS**P**Y**	QR**IYNS**P**Y**T	QR**IYNS**P**Y**T	QR**IYNS**P**Y**T
**2ARJ-H1**	F**S**L**TSNS**VH	**SNS**VH	GF**S**L**TSN**	GF**S**L**TSNS**
**2ARJ-H2**	WMGGI**WGD**G**D**TDY	GI**WGD**G**D**TDYNSALKS	**WGD**G**D**	I**WGD**G**D**T
**2ARJ-H3**	P**LIGSWY**F**D**F	**LIGSWY**F**D**F	**LIGSWY**F**D**F	TP**LIGSWY**F**D**F
**2B0S-L1**	SSNVE**NNY**V**Y**	SGTSSNVE**NNY**V**Y**	SGTSSNVE**NNY**V**Y**	SSNVE**NNY**
**2B0S-L2**	LLIY**RN**DHRSS	**RN**DHRSS	**RN**DHRSS	**RN**D
**2B0S-L3**	AA**WDD**SR**GGPD**W	AA**WDD**SR**GGPD**WV	AA**WDD**SR**GGPD**WV	AA**WDD**SR**GGPD**WV
**2B0S-H1**	YSF**SDYW**IG	**DYW**IG	GYSF**SDY**	GYSF**SDYW**
**2B0S-H2**	WMG**I**F**Y**P**GD**S**DSR**Y	**I**F**Y**P**GD**S**DSR**YSPSFEG	**Y**P**GD**S**D**	F**Y**P**GD**S**DS**
**2B0S-H3**	R**L**GG**DYEDSGAD**AFDF	**L**GG**DYEDSGAD**AFDF	**L**GG**DYEDSGAD**AFDF	AR**L**GG**DYEDSGAD**AFDF
**2B1A-L1**	SSNVE**NNYVY**	SGTSSNVE**NNYVY**	SGTSSNVE**NNYVY**	SSNVE**NNY**
**2B1A-L2**	LLIY**RN**DHRSS	**RN**DHRSS	**RN**DHRSS	**RN**D
**2B1A-L3**	AA**WDD**SR**GGP**DW	AA**WDD**SR**GGP**DWV	AA**WDD**SR**GGP**DWV	AA**WDD**SR**GGP**DWV
**2B1A-H1**	YSFS**DYW**IG	**DYW**IG	GYSFS**DY**	GYSFS**DYW**
**2B1A-H2**	WMG**I**F**Y**PG**D**S**DSR**Y	**I**F**Y**PG**D**S**DSR**YSPSFEG	**Y**PG**D**S**D**	F**Y**PG**D**S**DS**
**2B1A-H3**	R**L**G**GDYEDSGAD**AFDF	**L**G**GDYEDSGAD**AFDF	**L**G**GDYEDSGAD**AFDF	AR**L**G**GDYEDSGAD**AFDF
**2B1H-L1**	SSNVE**NNY**V**Y**	SGTSSNVE**NNY**V**Y**	SGTSSNVE**NNY**V**Y**	SSNVE**NNY**
**2B1H-L2**	LLIY**R**NDHRSS	**R**NDHRSS	**R**NDHRSS	**R**ND
**2B1H-L3**	AA**WDD**SR**GGP**DW	AA**WDD**SR**GGP**DWV	AA**WDD**SR**GGP**DWV	AA**WDD**SR**GGP**DWV
**2B1H-H1**	YSFS**DYW**IG	**DYW**IG	GYSFS**DY**	GYSFS**DYW**
**2B1H-H2**	WMG**I**F**Y**PG**D**S**DSR**Y	**I**F**Y**PG**D**S**DSR**YSPSFEG	**Y**PG**D**S**D**	F**Y**PG**D**S**DS**
**2B1H-H3**	R**L**GG**D**Y**EDSGAD**AFDF	**L**GG**D**Y**EDSGAD**AFDF	**L**GG**D**Y**EDSGAD**AFDF	AR**L**GG**D**Y**EDSGAD**AFDF
**2B2X-L1**	**SQVNH**MF	SAS**SQVNH**MF	SAS**SQVNH**MF	**SQVNH**
**2B2X-L2**	PWI**YL**TS**Y**LAS	**L**TS**Y**LAS	**L**TS**Y**LAS	**L**TS
**2B2X-L3**	Q**QWSGNPW**	Q**QWSGNPW**T	Q**QWSGNPW**T	Q**QWSGNPW**T
**2B2X-H1**	FTF**SR**Y**T**MS	**R**Y**T**MS	GFTF**SR**Y	GFTF**SR**Y**T**
**2B2X-H2**	**W**VA**VISGG**G**H**T**YY**	**VISGG**G**H**T**YYL**DSVEG	**SGG**G**H**	**ISGG**G**H**T
**2B2X-H3**	RG**FGDGG**YFDV	G**FGDGG**YFDV	G**FGDGG**YFDV	TRG**FGDGG**YFDV
**2B4C-L1**	QSV**S**SGSLA	RASQSV**S**SGSLA	RASQSV**S**SGSLA	QSV**S**SGS
**2B4C-L2**	LLIYGASTRAT	GASTRAT	GASTRAT	GAS
**2B4C-L3**	QQY**GTSP**Y	QQY**GTSP**YT	QQY**GTSP**YT	QQY**GTSP**YT
**2B4C-H1**	G**T**F**SM**YGFN	**M**YGFN	GG**T**F**SM**Y	GG**T**F**SM**YG
**2B4C-H2**	WMGGIIP**IF**G**T**S**NY**	GIIP**IF**G**T**S**NY**A**Q**KF**R**G	IP**IF**G**T**	IIP**IF**G**T**S
**2B4C-H3**	RDFG**PDWE**D**GDSYDG**SGR GFFDF	DFG**PDWE**D**GDSYDG**SGRGF FDF	DFG**PDWE**D**GDSYDG**SGR GFFDF	ARDFG**PDWE**D**GDSYDG**S GRGFFDF
**2BDN-L1**	EDI**YNR**LA	KATEDI**YNR**LA	KATEDI**YNR**LA	EDI**YNR**
**2BDN-L2**	LLI**SGA**T**S**L**ET**	**GA**T**S**L**ET**	**GA**T**S**L**ET**	**GA**T
**2BDN-L3**	QQ**FW**SAPY	QQ**FW**SAPYT	QQ**FW**SAPYT	QQ**FW**SAPYT
**2BDN-H1**	**LNIKDTY**MH	**DTY**MH	**GLNIKDT**	**GLNIKDTY**
**2BDN-H2**	WIG**R**I**DPA**NGNTKF	**R**I**DPA**NGNTKFDPKFQG	**DPA**NGN	I**DPA**NGNT
**2BDN-H3**	**RGVF**G**F**F**DY**	**GVF**G**F**F**DY**	**GVF**G**F**F**DY**	A**RGVF**G**F**F**DY**
**2BOC-L1**	**QS**IGT**D**IH	RAS**QS**IGT**D**IH	RAS**QS**IGT**D**IH	**QS**IGT**D**
**2BOC-L2**	LLIK**Y**ASESIS	**Y**ASESIS	**Y**ASESIS	**Y**AS

**2BOC-L3**	QQ**SNRWPF**	QQ**SNRWPF**T	QQ**SNRWPF**T	QQ**SNRWPF**T
**2BOC-H1**	YTF**TSDW**I**H**	**SDW**I**H**	GYTF**TSD**	GYTF**TSDW**
**2BOC-H2**	WIG**E**I**I**P**SY**G**R**A**N**Y	**E**I**I**P**SY**G**R**A**N**YN**E**KIQK	**I**P**SY**G**R**	I**I**P**SY**G**R**A
**2BOC-H3**	R**ERGDG**YFAV	**ERGDG**YFAV	**ERGDG**YFAV	AR**ERGDG**YFAV
**2BRR-L1**	SSVSSSYLH	RASSSVSSSYLH	RASSSVSSSYLH	SSVSSSY
**2BRR-L2**	LWIYSTSNLAS	STSNLAS	STSNLAS	STS
**2BRR-L3**	QQYSG**Y**P**Y**	QQYSG**Y**P**Y**T	QQYSG**Y**P**Y**T	QQYSG**Y**P**Y**T
**2BRR-H1**	YT**FTNYGMN**	**NYGMN**	GYT**FTNY**	GYT**FTNYG**
**2BRR-H2**	WMG**WINTYT**GEPTY	**WINTYT**GEPTYADDFKE	**NTYT**GE	**INTYT**GEP
**2BRR-H3**	R**D**Y**YGSTYP**Y**Y**AMDY	**D**Y**YGSTYP**Y**Y**AMDY	**D**Y**YGSTYP**Y**Y**AMDY	AR**D**Y**YGSTYP**Y**Y**AMDY
**2CK0-L1**	QNLL**H**SITRKN**Y**LA	KSSQNLL**H**SITRKN**Y**LA	KSSQNLL**H**SITRKN**Y**LA	QNLL**H**SITRKN**Y**
**2CK0-L2**	LLIYWASTRGS	WASTRGS	WASTRGS	WAS
**2CK0-L3**	K**QSYNLY**	K**QSYNLY**T	K**QSYNLY**T	K**QSYNLY**T
**2CK0-H1**	FTFNTDAM**N**	TDAM**N**	GFTFNTD	GFTFNTDA
**2CK0-H2**	**W**VA**R**I**R**S**KGFN**F**A**T**Y**Y	**R**I**R**S**KGFN**F**A**T**Y**YADSVRD	**R**S**KGFN**F**A**	I**R**S**KGFN**F**A**T
**2CK0-H3**	RGR**DG**EAMDY	GR**DG**EAMDY	GR**DG**EAMDY	VRGR**DG**EAMDY
**2CMR-L1**	EGI**YHW**LA	RASEGI**YHW**LA	RASEGI**YHW**LA	EGI**YHW**
**2CMR-L2**	LLIY**K**ASSLAS	**K**ASSLAS	**K**ASSLAS	**K**AS
**2CMR-L3**	QQ**YSNY**PL	QQ**YSNY**PLT	QQ**YSNY**PLT	QQ**YSNY**PLT
**2CMR-H1**	DTF**SSYA**IS	**SYA**IS	GDTF**SSY**	GDTF**SSYA**
**2CMR-H2**	**W**MG**GII**P**IFGTANY**	**GII**P**IFGTANY**AQAFQG	**I**P**IFGT**	**II**P**IFGTA**
**2CMR-H3**	R**DNPTLLG**SDY	**DNPTLLG**SDY	**DNPTLLG**SDY	AR**DNPTLLG**SDY
**2DD8-L1**	**N**I**GSK**SVH	GGN**N**I**GSK**SVH	GGN**N**I**GSK**SVH	**N**I**GSK**S
**2DD8-L2**	LVVYDDSDRPS	DDSDRPS	DDSDRPS	DDS
**2DD8-L3**	QV**WDSSSDY**	QV**WDSSSDY**V	QV**WDSSSDY**V	QV**WDSSSDY**V
**2DD8-H1**	GTFS**SYTI**S	**SYTI**S	GGTFS**SY**	GGTFS**SYT**
**2DD8-H2**	WMG**GIT**P**IL**G**IANY**	**GIT**P**IL**G**IANY**AQKFQG	**T**P**IL**G**I**	**IT**P**IL**G**IA**
**2DD8-H3**	R**DTVMGG**MDV	**DTVMGG**MDV	**DTVMGG**MDV	AR**DTVMGG**MDV
**2DTG-L1**	QSLL**YSSN**Q**K**N**F**LA	KSSQSLL**YSSN**Q**K**N**F**LA	KSSQSLL**YSSN**Q**K**N**F**LA	QSLL**YSSN**Q**K**N**F**
**2DTG-L2**	LLIY**W**ASTRES	**W**ASTRES	**W**ASTRES	**W**AS
**2DTG-L3**	QQ**YFR**YR	QQ**YFR**YRT	QQ**YFR**YRT	QQ**YFR**YRT
**2DTG-H1**	F**P**L**TAYG**VN	**AYG**VN	GF**P**L**TAY**	GF**P**L**TAYG**
**2DTG-H2**	WLGMI**WGD**G**N**T**D**Y	MI**WGD**G**N**T**D**YNSALKS	**WGD**G**N**	I**WGD**G**N**T
**2DTG-H3**	R**DPYGSK**PMDY	**DPYGSK**PMDY	**DPYGSK**PMDY	AR**DPYGSK**PMDY
**2EH8-L1**	QSLL**YSN**G**K**T**YLN**	KSSQSLL**YSN**G**K**T**YLN**	KSSQSLL**YSN**G**K**T**YLN**	QSLL**YSN**G**K**T**Y**
**2EH8-L2**	**R**LI**YL**VSKLDS	**L**VSKLDS	**L**VSKLDS	**L**VS
**2EH8-L3**	**VQGTHFPQ**	**VQGTHFPQ**T	**VQGTHFPQ**T	**VQGTHFPQ**T
**2EH8-H1**	YAFSSS**W**M**N**	SS**W**M**N**	GYAFSSS	GYAFSSS**W**
**2EH8-H2**	**W**IG**R**IYPGDGDT**N**Y	**R**IYPGDGDT**N**YAQKFQG	YPGDGD	IYPGDGDT
**2EH8-H3**	**REYD**EAY	**EYD**EAY	**EYD**EAY	**AREYD**EAY
**2F58-L1**	QGV**DFDGA**S**F**MN	KASQGV**DFDGA**S**F**MN	KASQGV**DFDGA**S**F**MN	QGV**DFDGA**S**F**
**2F58-L2**	LLIFAASTLES	AASTLES	AASTLES	AAS
**2F58-L3**	Q**QSHEDPL**	Q**QSHEDPL**T	Q**QSHEDPL**T	Q**QSHEDPL**T
**2F58-H1**	YSITSG**YS**W**H**	SG**YS**W**H**	GYSITSG**Y**	GYSITSG**Y**
**2F58-H2**	WMG**Y**I**HYS**A**GTN**Y	**Y**I**HYS**A**GTN**YNPSLKS	**HYS**A**G**	I**HYS**A**GT**
**2F58-H3**	R**E**EAMP**Y**GNQ**AYYY**AMDC	**E**EAMP**Y**GNQ**AYYY**AMDC	**E**EAMP**Y**GNQ**AYYY**AMDC	AR**E**EAMP**Y**GNQ**AYYY**AMDC
**2FJG-L1**	Q**DVS**TAVA	RASQ**DVS**TAVA	RASQ**DVS**TAVA	Q**DVS**TA
**2FJG-L2**	LLIYSASFLYS	SASFLYS	SASFLYS	SAS
**2FJG-L3**	QQS**YT**TPP	QQS**YT**TPPT	QQS**YT**TPPT	QQS**YT**TPPT
**2FJG-H1**	FTI**S**DY**W**IH	DY**W**IH	GFTI**S**DY	GFTI**S**DY**W**

2FJG-H2	WVAGITPAGGYTYY	GITPAGGYTYYADSVKG	TPAGGY	ITPAGGYT
2FJG-H3	RFVFFLPYAMDY	FVFFLPYAMDY	FVFFLPYAMDY	ARFVFFLPYAMDY
2FJH-L1	QVIRRSLA	RASQVIRRSLA	RASQVIRRSLA	QVIRRS
2FJH-L2	LLIYAASNLAS	AASNLAS	AASNLAS	AAS
2FJH-L3	QQSNTSPL	QQSNTSPLT	QQSNTSPLT	QQSNTSPLT
2FJH-H1	FTINASWIH	ASWIH	GFTINAS	GFTINASW
2FJH-H2	WVGAIYPYSGYTNY	AIYPYSGYTNYADSVKG	YPYSGY	IYPYSGYT
2FJH-H3	RWGHSTSPWAMDY	WGHSTSPWAMDY	WGHSTSPWAMDY	ARWGHSTSPWAMDY
2FX7-L1	QSVGNNKLA	RASQSVGNNKLA	RASQSVGNNKLA	QSVGNNK
2FX7-L2	LLIYGASSRPS	GASSRPS	GASSRPS	GAS
2FX7-L3	QQYGQSLS	QQYGQSLST	QQYGQSLST	QQYGQSLST
2FX7-H1	GSFSTYALS	TYALS	GGSFSTY	GGSFSTYA
2FX7-H2	WMGGVIPLLTITNY	GVIPLLTITNYAPRFQG	IPLLTI	VIPLLTIT
2FX7-H3	REGTTGWGWLGKPIGAFAH	EGTTGWGWLGKPIGAFAH	EGTTGWGWLGKPIGAFAH	AREGTTGWGWLGKPIGAFAH
2G5B-L1	QSLFNSKTRRNYLA	KSSQSLFNSKTRRNYLA	KSSQSLFNSKTRRNYLA	QSLFNSKTRRNY
2G5B-L2	LLIYWASTRES	WASTRES	WASTRES	WAS
2G5B-L3	KQSYNLR	KQSYNLRT	KQSYNLRT	KQSYNLRT
2G5B-H1	FTFIDNYMS	DNYMS	GFTFIDN	GFTFIDNY
2G5B-H2	WLGFIRNKVNGYTTEY	FIRNKVNGYTTEYGPSVKG	RNKVNGYT	IRNKVNGYTT
2G5B-H3	RDNGSDYRWYFDV	DNGSDYRWYFDV	DNGSDYRWYFDV	VRDNGSDYRWYFDV
2GSI-L1	KSVRTSGYSYMD	GASKSVRTSGYSYMD	GASKSVRTSGYSYMD	KSVRTSGYSY
2GSI-L2	RLIYLVSNLES	LVSNLES	LVSNLES	LVS
2GSI-L3	SHIRELPR	SHIRELPRS	SHIRELPRS	SHIRELPRS
2GSI-H1	NIKDYYMYW	DYYMY	GFNIKDY	GFNIKDYY
2GSI-H2	IGWIDPENGDTEYV	WIDPENGDTEYVPTFQG	DPENGD	IDPENGDT
2GSI-H3	GVITMMGYQA	GVITMAMDY	GVITMAMDY	NAGVITMAMDY
2H1P-L1	QSLVHSNGNTYLH	RSSQSLVHSNGNTYLH	RSSQSLVHSNGNTYLH	QSLVHSNGNTY
2H1P-L2	LLIYKVSNRFS	KVSNRFS	KVSNRFS	KVS
2H1P-L3	SQSTHVPW	SQSTHVPWT	SQSTHVPWT	SQSTHVPWT
2H1P-H1	FTFSSYFLS	SYFLS	GFTFSSY	GFTFSSYF
2H1P-H2	LVATINSNGDKTYH	TINSNGDKTYHPDTMKG	NSNGDK	INSNGDKT
2H1P-H3	RRDSSASLYFDY	RDSSASLYFDY	RDSSASLYFDY	ARRDSSASLYFDY
2H9G-L1	QDVSTAVA	RASQDVSTAVA	RASQDVSTAVA	QDVSTA
2H9G-L2	LLIYSASFLYS	SASFLYS	SASFLYS	SAS
2H9G-L3	QQSYTTPP	QQSYTTPPT	QQSYTTPPT	QQSYTTPPT
2H9G-H1	FSIGKSGIH	KSGIH	GFSIGKS	GFSIGKSG
2H9G-H2	WVAVIYPHDGNTAY	VIYPHDGNTAYADSVKG	YPHDGN	IYPHDGNT
2H9G-H3	RRLALVRMWMDY	RLALVRMWMDY	RLALVRMWMDY	ARRLALVRMWMDY
2HFG-L1	QDVSTAVA	RASQDVSTAVA	RASQDVSTAVA	QDVSTA
2HFG-L2	LLIYSASFLYS	SASFLYS	SASFLYS	SAS
2HFG-L3	QQSQISPP	QQSQISPPT	QQSQISPPT	QQSQISPPT
2HFG-H1	FTISSSSIH	SSSIH	GFTISSS	GFTISSSS
2HFG-H2	WVAWVLPSVGFTDY	WVLPSVGFTDYADSVKG	LPSVGF	VLPSVGFT
2HFG-H3	RRVCYNRLGVCAGGMDY	RVCYNRLGVCAGGMDY	RVCYNRLGVCAGGMDY	ARRVCYNRLGVCAGGMDY
2HH0-L1	QDIGNNLN	RASQDIGNNLN	RASQDIGNNLN	QDIGNN
2HH0-L2	RLIYATSSLDS	ATSSLDS	ATSSLDS	ATS
2HH0-L3	LQHDTFPL	LQHDTFPLT	LQHDTFPLT	LQHDTFPLT
2HH0-H1	NIEDSYIHW	DSYIH	GFNIEDS	GFNIEDSY
2HH0-H2	IGRIDPEDGETKYA	RIDPEDGETKYAPKFQG	DPEDGE	IDPEDGET

2HH0-H3	GAYYI	GAYYIKEDF	GAYYIKEDF	GRGAYYIKEDF
2HKF-L1	QSLVHSNGNTYLH	RSSQSLVHSNGNTYLH	RSSQSLVHSNGNTYLH	QSLVHSNGNTY
2HKF-L2	LLIYKVSNRFS	KVSNRFS	KVSNRFS	KVS
2HKF-L3	SQSTHVPF	SQSTHVPFT	SQSTHVPFT	SQSTHVPFT
2HKF-H1	STLNNYAMN	NYAMN	GSTLNNY	GSTLNNYA
2HKF-H2	WVARIRSKSNNYATYY	RIRSKSNNYATYYADSVKD	RSKSNNYA	IRSKSNNYAT
2HKF-H3	TYGNHPFAY	YGNHPFAY	YGNHPFAY	VTYGNHPFAY
2HRP-L1	ESVDYYGKSFMN	RASESVDYYGKSFMN	RASESVDYYGKSFMN	ESVDYYGKSF
2HRP-L2	LLIYAASNQGS	AASNQGS	AASNQGS	AAS
2HRP-L3	QQSKEVPW	QQSKEVPWT	QQSKEVPWT	QQSKEVPWT
2HRP-H1	FTFMRFGMH	RFGMH	GFTFMRF	GFTFMRFG
2HRP-H2	WVAYISSGSSTIYY	YISSGSSTIYYADTVKG	SSGSST	ISSGSSTI
2HRP-H3	RSGGIERYDGTYYVMDY	SGGIERYDGTYYVMDY	SGGIERYDGTYYVMDY	ARSGGIERYDGTYYVMDY
2I9L-L1	QTLLNSRTRKNYLA	KSSQTLLNSRTRKNYLA	KSSQTLLNSRTRKNYLA	QTLLNSRTRKNY
2I9L-L2	LLIYWASTRES	WASTRES	WASTRES	WAS
2I9L-L3	KQSYNLW	KQSYNLWT	KQSYNLWT	KQSYNLWT
2I9L-H1	YTFTRYWMH	RYWMH	GYTFTRY	GYTFTRYW
2I9L-H2	WIGYINPSTGYTEY	YINPSTGYTEYNQKFKD	NPSTGY	INPSTGYT
2I9L-H3	RTTVDGYDFAY	TTVDGYDFAY	TTVDGYDFAY	ARTTVDGYDFAY
2IFF-L1	SSVNYMY	SASSSVNYMY	SASSSVNYMY	SSVNY
2IFF-L2	RWIYDTSKLAS	DTSKLAS	DTSKLAS	DTS
2IFF-L3	QQWGRNP	QQWGRNPT	QQWGRNPT	QQWGRNPT
2IFF-H1	YTFSDYWIE	DYWIE	GYTFSDY	GYTFSDYW
2IFF-H2	WIGEILPGSGSTNY	EILPGSGSTNYHERFKG	LPGSGS	ILPGSGST
2IFF-H3	HGNYDFDG	GNYDFDG	GNYDFDG	LHGNYDFDG
2IGF-L1	QTILLSDGDTYLE	RSNQTILLSDGDTYLE	RSNQTILLSDGDTYLE	QTILLSDGDTY
2IGF-L2	LLIYKVSNRFS	KVSNRFS	KVSNRFS	KVS
2IGF-L3	FQGSHVPP	FQGSHVPPT	FQGSHVPPT	FQGSHVPPT
2IGF-H1	FTFSRCAMS	RCAMS	GFTFSRC	GFTFSRCA
2IGF-H2	WVAGISSGGSYTFY	GISSGGSYTFYPDTVKG	SSGGSY	ISSGGSYT
2IGF-H3	RYSSDPFYFDY	YSSDPFYFDY	YSSDPFYFDY	TRYSSDPFYFDY
2IPU-L1	QSIVHSNGNTYLE	RSSQSIVHSNGNTYLE	RSSQSIVHSNGNTYLE	QSIVHSNGNTY
2IPU-L2	LLIYKVSNRFS	KVSNRFS	KVSNRFS	KVS
2IPU-L3	FQGSHVPL	FQGSHVPLT	FQGSHVPLT	FQGSHVPLT
2IPU-H1	FSLSTSGMGVG	TSGMGVG	GFSLSTSGM	GFSLSTSG
2IPU-H2	WLAHIWWDDDRSY	HIWWDDDRSYNPSLKS	WWDDD	IWWDDDR
2IPU-H3	RRAHTTVLGDWFAY	RAHTTVLGDWFAY	RAHTTVLGDWFAY	VRRAHTTVLGDWFAY
2J4W-L1	SSVSYMH	RARSSVSYMH	RARSSVSYMH	SSVSY
2J4W-L2	PWIHATSNLAS	ATSNLAS	ATSNLAS	ATS
2J4W-L3	QQWSSHPP	QQWSSHPPT	QQWSSHPPT	QQWSSHPPT
2J4W-H1	FIFSDYYMY	DYYMY	GFIFSDY	GFIFSDYY
2J4W-H2	WVATISDGNSYTYY	TISDGNSYTYYVDSVKG	SDGNSY	ISDGNSYT
2J4W-H3	RDGPTDSSGYGGFGY	DGPTDSSGYGGFGY	DGPTDSSGYGGFGY	ARDGPTDSSGYGGFGY
2J5L-L1	SSVSYMH	RARSSVSYMH	RARSSVSYMH	SSVSY
2J5L-L2	PWIHATSNLAS	ATSNLAS	ATSNLAS	ATS
2J5L-L3	QQWSSHPP	QQWSSHPPT	QQWSSHPPT	QQWSSHPPT
2J5L-H1	FIFSDYYMY	DYYMY	GFIFSDY	GFIFSDYY
2J5L-H2	WVATISDGNSYTYY	TISDGNSYTYYVDSVKG	SDGNSY	ISDGNSYT
2J5L-H3	RDGPTDSSGYGGFGY	DGPTDSSGYGGFGY	DGPTDSSGYGGFGY	ARDGPTDSSGYGGFGY
2J6E-L1	SSNIGSNYVY	SGSSSNIGSNYVY	SGSSSNIGSNYVY	SSNIGSNY

**2J6E-L2**	LLIY**R**NN**Q**RPS	**R**NN**Q**RPS	**R**NN**Q**RPS	**R**NN
**2J6E-L3**	AT**W**D**D**SL**S**AV	AT**W**D**D**SL**S**AVI	AT**W**D**D**SL**S**AVI	AT**W**D**D**SL**S**AVI
**2J6E-H1**	GSISRGSH**Y**WG	RGSH**Y**WG	GGSISRGSH	GGSISRGS
**2J6E-H2**	WIGSI**Y**YSGNT**Y**F	SI**Y**YSGNT**Y**FNPSLKS	**Y**YSGN	I**Y**YSGNT
**2J6E-H3**	RLGP**DDYTLDG**MDV	LGP**DDYTLDG**MDV	LGP**DDYTLDG**MDV	ARLGP**DDYTLDG**MDV
**2J88-L1**	N**IYSY**L**T**W	RASEN**IYSY**L**T**	RASEN**IYSY**L**T**	EN**IYSY**
**2J88-L2**	L**VYN**AKTLAEG	**N**AKTLAE	**N**AKTLAE	**N**AK
**2J88-L3**	H**HYGTR**T	QH**HYGTR**T	QH**HYGTR**T	QH**HYGTR**T
**2J88-H1**	FSLST**SGMG**VS	T**SGMG**VS	GFSLST**SGM**	GFSLST**SG**
**2J88-H2**	WLA**H**I**YWD**D**D**K**R**Y	**H**I**YWD**D**D**K**R**YNPSLKS	**YWD**D**D**	I**YWD**D**D**K
**2J88-H3**	L**YYGSVD**Y	**YYGSVD**Y	**YYGSVD**Y	TL**YYGSVD**Y
**2JEL-L1**	QSIV**HGN**G**N**T**Y**LE	RSSQSIV**HGN**G**N**T**Y**LE	RSSQSIV**HGN**G**N**T**Y**LE	QSIV**HGN**G**N**T**Y**
**2JEL-L2**	LLIY**K**ISNRFS	**K**ISNRFS	**K**ISNRFS	**K**IS
**2JEL-L3**	FQ**GSHV**P**Y**	FQ**GSHV**P**Y**T	FQ**GSHV**P**Y**T	FQ**GSHV**P**Y**T
**2JEL-H1**	YT**FTTYAMH**	**TYAMH**	GYT**FTTY**	GYT**FTTYA**
**2JEL-H2**	WIG**LISTYS**G**Y**T**N**Y	**LISTYS**G**Y**T**N**YNQKFKG	**STYS**G**Y**	**ISTYS**G**Y**T
**2JEL-H3**	R**VMGEQY**FDV	**VMGEQY**FDV	**VMGEQY**FDV	AR**VMGEQY**FDV
**2JIX-L1**	QGIRNDLG	RASQGIRNDLG	RASQGIRNDLG	QGIRND
**2JIX-L2**	RLIYAA**SSLQS**	AA**SSLQS**	AA**SSLQS**	AA**S**
**2JIX-L3**	LQHNTYPP	LQHNTYPPT	LQHNTYPPT	LQHNTYPPT
**2JIX-H1**	ASISSYYWS	SYYWS	GASISSY	GASISSYY
**2JIX-H2**	WIGYIGGEGSTNY	YIGGEGSTNYNPSLKS	GGEGS	IGGEGST
**2JIX-H3**	RERLGIGDY	ERLGIGDY	ERLGIGDY	ARERLGIGDY
**2NR6-L1**	QIV**STA**VA	KASQIV**STA**VA	KASQIV**STA**VA	QIV**STA**
**2NR6-L2**	LLI**YS**A**SY**RYT	**S**A**SY**RYT	**S**A**SY**RYT	**S**A**S**
**2NR6-L3**	**QQHYNSPQ**	**QQHYNSPQ**T	**QQHYNSPQ**T	**QQHYNSPQ**T
**2NR6-H1**	FN**IKDTY**I**H**	**DTY**I**H**	GFN**IKDT**	GFN**IKDTY**
**2NR6-H2**	**W**IG**RIDPAN**GNT**R**Y	**RIDPAN**GNT**R**YGPKFLG	**DPAN**GN	**IDPAN**GNT
**2NR6-H3**	R**WVRQ**MDY	**WVRQ**MDY	**WVRQ**MDY	AR**WVRQ**MDY
**2NY7-L1**	HSIRSRRVA	RSSHSIRSRRVA	RSSHSIRSRRVA	HSIRSRR
**2NY7-L2**	LVIHGVSNRAS	GVSNRAS	GVSNRAS	GVS
**2NY7-L3**	QVYGASSY	QVYGASSYT	QVYGASSYT	QVYGASSYT
**2NY7-H1**	Y**R**F**SNFV**IH	**NFV**IH	GY**R**F**SNF**	GY**R**F**SNFV**
**2NY7-H2**	WMG**W**I**NPYN**G**NKE**F	**W**I**NPYN**G**NKE**FSAKFQD	**NPYN**G**N**	I**NPYN**G**NK**
**2NY7-H3**	R**VGPYSWDD**S**PQ**D**NY**YMDV	**VGPYSWDD**S**PQ**D**NY**YMDV	**VGPYSWDD**S**PQ**D**NY**YMDV	AR**VGPYSWDD**S**PQ**D**NY**YMDV
**2NZ9-L1**	**ES**V**DSYGHSF**MQ	RAS**ES**V**DSYGHSF**MQ	RAS**ES**V**DSYGHSF**MQ	**ES**V**DSYGHSF**
**2NZ9-L2**	LLIYRASNLEP	RASNLEP	RASNLEP	RAS
**2NZ9-L3**	QQ**GNEV**P**F**	QQ**GNEV**P**F**T	QQ**GNEV**P**F**T	QQ**GNEV**P**F**T
**2NZ9-H1**	FTF**SDHY**M**Y**	**DHY**M**Y**	GFTF**SDH**	GFTF**SDHY**
**2NZ9-H2**	WVA**T**I**SDGGSYTYY**	**T**I**SDGGSYTYY**SDSVEG	**SDGGSY**	I**SDGGSYT**
**2NZ9-H3**	R**Y**R**YD**DAMDY	**Y**R**YD**DAMDY	**Y**R**YD**DAMDY	SR**Y**R**YD**DAMDY
**2OQJ-L1**	**QSIE**T**W**LA	RAS**QSIE**T**W**LA	RAS**QSIE**T**W**LA	**QSIE**T**W**
**2OQJ-L2**	LLIYKASTLKT	KASTLKT	KASTLKT	KAS
**2OQJ-L3**	Q**HYAGYS**A	Q**HYAGYS**AT	Q**HYAGYS**AT	Q**HYAGYS**AT
**2OQJ-H1**	FRISAHTMN	AHTMN	NFRISAH	NFRISAHT
**2OQJ-H2**	WVASISTSS**TY**R**D**Y	SISTSS**TY**R**D**YADAVKG	STSS**TY**	ISTSS**TY**R
**2OQJ-H3**	RKGSDRLS**DN**DPFDA	KGSDRLS**DN**DPFDA	KGSDRLS**DN**DPFDA	ARKGSDRLS**DN**DPFDA
**2OR9-L1**	ESVDNYG**F**SFMN	RASESVDNYG**F**SFMN	RASESVDNYG**F**SFMN	ESVDNYG**F**SF
**2OR9-L2**	**L**LI**YA**I**SN**RGS	**A**I**SN**RGS	**A**I**SN**RGS	**A**I**S**
**2OR9-L3**	QQTKEVPW	QQTKEVPWT	QQTKEVPWT	QQTKEVPWT

**2OR9-H1**	FTF**SHYG**MS	**HYG**MS	GFTF**SHY**	GFTF**SHYG**
**2OR9-H2**	WVAT**IGSR**GTYTHY	T**IGSR**GTYTHYPDSVKG	**GSR**GTY	**IGSR**GTYT
**2OR9-H3**	R**RSEFYYYG**N**TYYY**S**A**M**D** Y	**RSEFYYYG**N**TYYY**S**A**M**D**Y	**RSEFYYYG**N**TYYY**S**A**M**D** Y	AR**RSEFYYYG**N**TYYY**S**A** M**D**Y
**2OSL-L1**	SSVSYIH	RASSSVSYIH	RASSSVSYIH	SSVSY
**2OSL-L2**	PWIYATSNLAS	ATSNLAS	ATSNLAS	ATS
**2OSL-L3**	QQ**W**T**SNPP**	QQ**W**T**SNPP**T	QQ**W**T**SNPP**T	QQ**W**T**SNPP**T
**2OSL-H1**	YTFTSY**NMH**	SY**NMH**	GYTFTSY	GYTFTSY**N**
**2OSL-H2**	**W**I**GAIY**PG**N**G**DTSY**	**AIY**PG**N**G**DTSY**NQKFKG	**Y**PG**N**G**D**	**IY**PG**N**G**DT**
**2OSL-H3**	R**ST**Y**Y**GGD**W**YFNV	**ST**Y**Y**GGD**W**YFNV	**ST**Y**Y**GGD**W**YFNV	AR**ST**Y**Y**GGD**W**YFNV
**2OTU-L1**	**SQHSTYTIE**	TL**SSQHSTYTIE**	TL**SSQHSTYTIE**	**SQHSTYT**
**2OTU-L2**	YVM**E**LKKDGSHSTGD	LKKDGSHSTGD	LKKDGSHSTGD	LKK
**2OTU-L3**	G**VGDTIK**EQ**F**VY	G**VGDTIK**EQ**F**VYV	G**VGDTIK**EQ**F**VYV	G**VGDTIK**EQ**F**VYV
**2OTU-H1**	FTF**RDYYMY**	**DYYMY**	GFTF**RDY**	GFTF**RDYY**
**2OTU-H2**	WVA**F**IS**N**GGGST**Y**Y	**F**IS**N**GGGST**Y**YPDTVKG	S**N**GGGS	IS**N**GGGST
**2OTU-H3**	**RGRGYVWF**AY	**GRGYVWF**AY	**GRGYVWF**AY	A**RGRGYVWF**AY
**2OZ4-L1**	QS**IGTS**I**H**	RASQS**IGTS**I**H**	RASQS**IGTS**I**H**	QS**IGTS**
**2OZ4-L2**	**L**LI**EY**ASES**IS**	**Y**ASES**IS**	**Y**ASES**IS**	**Y**AS
**2OZ4-L3**	**Q**Q**SNVW**P**F**	**Q**Q**SNVW**P**F**T	**Q**Q**SNVW**P**F**T	**Q**Q**SNVW**P**F**T
**2OZ4-H1**	Y**TFSEFTMH**	**EFTMH**	**G**Y**TFSEF**	**G**Y**TFSEFT**
**2OZ4-H2**	WIG**GINTINGGSS**Y	**GINTINGGSS**YKQSF**K**D	**NTINGG**	**INTINGGS**
**2OZ4-H3**	**TKGFAY**	**KGFAY**	**KGFAY**	A**TKGFAY**
**2Q8A-L1**	QSVSN**DVV**	KASQSVSN**DVV**	KASQSVSN**DVV**	QSVSN**D**
**2Q8A-L2**	**L**LI**YY**AS**IRYT**	**Y**AS**IRYT**	**Y**AS**IRYT**	**Y**AS
**2Q8A-L3**	QQ**GF**SSP**R**	QQ**GF**SSP**R**T	QQ**GF**SSP**R**T	QQ**GF**SSP**R**T
**2Q8A-H1**	**FKIKDTS**M**H**	**DTS**M**H**	**GFKIKDT**	**GFKIKDTS**
**2Q8A-H2**	WIG**R**I**D**P**A**NDNS**E**Y	**R**I**D**P**A**NDNS**E**YDPKFQG	**D**P**A**NDN	I**D**P**A**NDNS
**2Q8A-H3**	**LSHF**	**SHF**	**SHF**	T**LSHF**
**2QAD-L1**	QSISN**W**LA	RASQSISN**W**LA	RASQSISN**W**LA	QSISN**W**
**2QAD-L2**	LLMYKASSLKS	KASSLKS	KASSLKS	KAS
**2QAD-L3**	QQ**HDSS**P**Y**	QQ**HDSS**P**Y**T	QQ**HDSS**P**Y**T	QQ**HDSS**P**Y**T
**2QAD-H1**	GTFSNYAIN	NYAIN	GGTFSNY	GGTFSNYA
**2QAD-H2**	WMGGI**IPIF**N**IAH**Y	GI**IPIF**N**IAH**YAQRFQG	**IPIF**N**I**	I**IPIF**N**IA**
**2QAD-H3**	SPYPNDY**ND**Y**APEEGM**S**W** YFDL	PYPND**NDAPEEGM**S**W**YFDL	PYPND**NDAPEEGM**S**W**YF DL	ASPYPND**NDAPEEGM**S**W** YFDL
**2QHR-L1**	R**QHSTYTI**E	TLSR**QHSTYTI**E	TLSR**QHSTYTI**E	R**QHSTYT**
**2QHR-L2**	YVMELKKDGSHSTGD	LKKDGSHSTGD	LKKDGSHSTGD	LKK
**2QHR-L3**	G**VGDTIK**EQ**F**VY	G**VGDTIK**EQ**F**VYV	G**VGDTIK**EQ**F**VYV	G**VGDTIK**EQ**F**VYV
**2QHR-H1**	FAFS**S**Y**D**MS	**S**Y**D**MS	GFAFS**S**Y	GFAFS**S**Y**D**
**2QHR-H2**	WVA**Y**I**SRGGGY**T**Y**Y	**Y**I**SRGGGY**T**Y**YPDTVKG	**SRGGGY**	I**SRGGGY**T
**2QHR-H3**	R**HIYYG**S**SHYYA**MDY	**HIYYG**S**SHYYA**MDY	**HIYYG**S**SHYYA**MDY	SR**HIYYG**S**SHYYA**MDY
**2QQL-L1**	Q**D**V**S**TAVA	RASQ**D**V**S**TAVA	RASQ**D**V**S**TAVA	Q**D**V**S**TA
**2QQL-L2**	LLIYSASFLYS	SASFLYS	SASFLYS	SAS
**2QQL-L3**	QQA**WAY**LP	QQA**WAY**LPT	QQA**WAY**LPT	QQA**WAY**LPT
**2QQL-H1**	FTISGYGIH	GYGIH	GFTISGY	GFTISGYG
**2QQL-H2**	WVA**Y**I**Y**PD**S**G**Y**T**D**Y	**Y**I**Y**PD**S**G**Y**T**D**YADSVKG	**Y**PD**S**G**Y**	I**Y**PD**S**G**Y**T
**2QQL-H3**	R**E**D**FRNRR**RL**WY**VMDY	**E**D**FRNRR**RL**WY**VMDY	**E**D**FRNRR**RL**WY**VMDY	AR**E**D**FRNRR**RL**WY**VMDY
**2QQN-L1**	Q**YFSSY**LA	RASQ**YFSSY**LA	RASQ**YFSSY**LA	Q**YFSSY**
**2QQN-L2**	LLIYGAS**S**RAS	GAS**S**RAS	GAS**S**RAS	GAS
**2QQN-L3**	QQY**L**GSPP	QQY**L**GSPPT	QQY**L**GSPPT	QQY**L**GSPPT
**2QQN-H1**	FTFSSYAMS	SYAMS	GFTFSSY	GFTFSSYA

**2QQN-H2**	WVSQISPAGGYTNY	QISPAGGYTNYADSVKG	SPAGGY	ISPAGGYT
**2QQN-H3**	RGELP**YYRMS**KVMDV	GELP**YYRMS**KVMDV	GELP**YYRMS**KVMDV	ARGELP**YYRMS**KVMDV
**2QR0-L1**	QSV**S**SAVA	RASQSV**S**SAVA	RASQSV**S**SAVA	QSV**S**SA
**2QR0-L2**	LLIYSASSLYS	SASSLYS	SASSLYS	SAS
**2QR0-L3**	QQ**YSY**YYY**P**F	QQ**YSY**YYY**P**FT	QQ**YSY**YYY**P**FT	QQ**YSY**YYY**P**FT
**2QR0-H1**	FNFS**SSS**I**H**	**SSS**I**H**	GFNFS**SS**	GFNFS**SSS**
**2QR0-H2**	WVA**Y**I**Y**PS**Y**S**Y**TSY	**Y**I**Y**PS**Y**S**Y**TSYADSVKG	**Y**PS**Y**S**Y**	I**Y**PS**Y**S**Y**T
**2QR0-H3**	R**YYGTG**AMDY	**YYGTG**AMDY	**YYGTG**AMDY	AR**YYGTG**AMDY
**2QSC-L1**	QDISN**Y**LN	QASQDISN**Y**LN	QASQDISN**Y**LN	QDISN**Y**
**2QSC-L2**	LLIYTASNLET	TASNLET	TASNLET	TAS
**2QSC-L3**	QQ**YDNLG**DL	QQ**YDNLG**DLS	QQ**YDNLG**DLS	QQ**YDNLG**DLS
**2QSC-H1**	FNFSSY**VMH**	SY**VMH**	GFNFSSY	GFNFSSY**V**
**2QSC-H2**	YLS**AISS**DGE**TTY**H	**AISS**DGE**TTY**HANSVKG	**SS**DGE**T**	**ISS**DGE**TT**
**2QSC-H3**	R**D**R**Y**YETS**GSN**AFDV	**D**R**Y**YETS**GSN**AFDV	**D**R**Y**YETS**GSN**AFDV	AR**D**R**Y**YETS**GSN**AFDV
**2R0K-L1**	QDVSTAVA	RASQDVSTAVA	RASQDVSTAVA	QDVSTA
**2R0K-L2**	LLIYSASFLYS	SASFLYS	SASFLYS	SAS
**2R0K-L3**	Q**QSYTTPP**	Q**QSYTTPP**T	Q**QSYTTPP**T	Q**QSYTTPP**T
**2R0K-H1**	**FTITGSA**I**H**	**GSA**I**H**	**GFTITGS**	**GFTITGSA**
**2R0K-H2**	**W**VA**IINPNGGYT**YY	**IINPNGGYT**YYADSVKG	**NPNGGY**	**INPNGGYT**
**2R0K-H3**	**RSARF**S**FD**Y	**SARF**S**FD**Y	**SARF**S**FD**Y	A**RSARF**S**FD**Y
**2R0L-L1**	Q**DVS**T**A**VA	RASQ**DVS**T**A**VA	RASQ**DVS**T**A**VA	Q**DVS**T**A**
**2R0L-L2**	LLIYSASFLYS	SASFLYS	SASFLYS	SAS
**2R0L-L3**	**QQSYTTPP**	**QQSYTTPP**T	**QQSYTTPP**T	**QQSYTTPP**T
**2R0L-H1**	FTI**SNS**GI**H**	**NS**GI**H**	GFTI**SNS**	GFTI**SNS**G
**2R0L-H2**	**W**VG**W**I**YPTGGATD**Y	**W**I**YPTGGATD**YADSVKG	**YPTGGA**	I**YPTGGAT**
**2R0L-H3**	**RFWWR**S**F**DY	**FWWR**S**F**DY	**FWWR**S**F**DY	A**RFWWR**S**F**DY
**2R0W-L1**	QSI**VHSN**G**N**T**Y**LE	RSSQSI**VHSN**G**N**T**Y**LE	RSSQSI**VHSN**G**N**T**Y**LE	QSI**VHSN**G**N**T**Y**
**2R0W-L2**	LLIYKVSNRFS	KVSNRFS	KVSNRFS	KVS
**2R0W-L3**	FQ**GSHVPL**	FQ**GSHVPL**T	FQ**GSHVPL**T	FQ**GSHVPL**T
**2R0W-H1**	FSLRTS**G**MGVG	TS**G**MGVG	GFSLRTS**G**M	GFSLRTS**G**
**2R0W-H2**	**W**LA**H**I**WWD**D**D**K**N**Y	**H**I**WWD**D**D**K**N**YNPSLKS	**WWD**D**D**	I**WWD**D**D**K
**2R0W-H3**	R**RAHNV**VLG**D**WFAY	**RAHNV**VLG**D**WFAY	**RAHNV**VLG**D**WFAY	VR**RAHNV**VLG**D**WFAY
**2R29-L1**	ESVV**RYGN**S**F**MH	RASESVV**RYGN**S**F**MH	RASESVV**RYGN**S**F**MH	ESVV**RYGN**S**F**
**2R29-L2**	**L**LI**YR**AS**SLES**	**R**AS**SLES**	**R**AS**SLES**	**R**AS
**2R29-L3**	QQTNVDPW	QQTNVDPWA	QQTNVDPWA	QQTNVDPWA
**2R29-H1**	**FN**I**KDTY**MH	**DTY**MH	**GFN**I**KDT**	**GFN**I**KDTY**
**2R29-H2**	WIGRI**DPAN**GYSKY	RI**DPAN**GYSKYDPKFQG	**DPAN**GY	I**DPAN**GYS
**2R29-H3**	**RDYEG**F**AY**	**DYEG**F**AY**	**DYEG**F**AY**	A**RDYEG**F**AY**
**2R4S-L1**	QDINS**Y**L**S**	KASQDINS**Y**L**S**	KASQDINS**Y**L**S**	QDINS**Y**
**2R4S-L2**	**T**LI**YR**ANR**LVD**	**R**ANR**LVD**	**R**ANR**LVD**	**R**AN
**2R4S-L3**	LQ**YDEF**P**Y**	LQ**YDEF**P**Y**T	LQ**YDEF**P**Y**T	LQ**YDEF**P**Y**T
**2R4S-H1**	YIFT**DYYIN**	**DYYIN**	GYIFT**DY**	GYIFT**DYY**
**2R4S-H2**	WIG**E**I**Y**PG**SGN**IDY	**E**I**Y**PG**SGN**IDYNERFKD	**Y**PG**SGN**	I**Y**PG**SGN**I
**2R4S-H3**	**RGFGY**	**GFGY**	**GFGY**	V**RGFGY**
**2R56-L1**	QGI**SSRL**A	RASQGI**SSRL**A	RASQGI**SSRL**A	QGI**SSR**
**2R56-L2**	LLI**YAASSL**QS	**AASSL**QS	**AASSL**QS	**AAS**
**2R56-L3**	QQ**YH**SYPW	QQ**YH**SYPWT	QQ**YH**SYPWT	QQ**YH**SYPWT
**2R56-H1**	FTF**RHH**GMT	**HH**GMT	GFTF**RHH**	GFTF**RHH**G
**2R56-H2**	WVASL**SGSGTK**THF	SL**SGSGTK**THFADSVKG	**SGSGTK**	L**SGSGTK**T
**2R56-H3**	KA**KRVGAT**G**Y**FDL	A**KRVGAT**G**Y**FDL	A**KRVGAT**G**Y**FDL	AKA**KRVGAT**G**Y**FDL

**2UZI-L1**	QS**IS**S**Y**LN	RASQS**IS**S**Y**LN	RASQS**IS**S**Y**LN	QS**IS**S**Y**
**2UZI-L2**	LLIYSASVLQS	SASVLQS	SASVLQS	SAS
**2UZI-L3**	QQ**SVMI**P**M**	QQ**SVMI**P**M**T	QQ**SVMI**P**M**T	QQ**SVMI**P**M**T
**2UZI-H1**	F**T**F**STFSM**N	**TFSM**N	GF**T**F**STF**	GF**T**F**STFS**
**2UZI-H2**	WVS**Y**I**SRTSKT**I**Y**Y	**Y**I**SRTSKT**I**Y**YADSVKG	**SRTSKT**	I**SRTSKT**I
**2UZI-H3**	**RGRF**F**D**Y	**GRF**F**D**Y	**GRF**F**D**Y	A**RGRF**F**D**Y
**2V17-L1**	KSI**RKF**LA	RASKSI**RKF**LA	RASKSI**RKF**LA	KSI**RKF**
**2V17-L2**	LLI**YS**GSTLQS	**S**GSTLQS	**S**GSTLQS	**S**GS
**2V17-L3**	QQ**HNDY**P**L**	QQ**HNDY**P**L**T	QQ**HNDY**P**L**T	QQ**HNDY**P**L**T
**2V17-H1**	FTFTDY**Y**MS	DY**Y**MS	GFTFTDY	GFTFTDY**Y**
**2V17-H2**	WLA**L**I**R**NKA**K**GYTT**E**Y	**L**I**R**NKA**K**GYTT**E**YSASVKG	**R**NKA**K**GYT	I**R**NKA**K**GYTT
**2V17-H3**	RDN**GAARAT**FAY	DN**GAARAT**FAY	DN**GAARAT**FAY	ARDN**GAARAT**FAY
**2VDK-L1**	QG**ISSN**IG	HASQG**ISSN**IG	HASQG**ISSN**IG	QG**ISSN**
**2VDK-L2**	GLI**YY**GT**NL**V**D**	**Y**GT**NL**V**D**	**Y**GT**NL**V**D**	**Y**GT
**2VDK-L3**	VQ**YAQLPY**	VQ**YAQLPY**T	VQ**YAQLPY**T	VQ**YAQLPY**T
**2VDK-H1**	FNIK**DTY**VH	**DTY**VH	GFNIK**DT**	GFNIK**DTY**
**2VDK-H2**	WIG**R**I**D**PA**N**G**Y**T**K**Y	**R**I**D**PA**N**G**Y**T**K**YDPKFQG	**D**PA**N**G**Y**	I**D**PA**N**G**Y**T
**2VDK-H3**	**RPLYDYY**AMDY	**PLYDYY**AMDY	**PLYDYY**AMDY	V**RPLYDYY**AMDY
**2VIS-L1**	TGAVTTSNYAN	RSSTGAVTTSNYAN	RSSTGAVTTSNYAN	TGAVTTSNY
**2VIS-L2**	GLIGGTNNRAP	GTNNRAP	GTNNRAP	GTN
**2VIS-L3**	ALWYS**N**HW	ALWYS**N**HWV	ALWYS**N**HWV	ALWYS**N**HWV
**2VIS-H1**	F**L**L**ISN**GVH	**SN**GVH	GF**L**L**ISN**	GF**L**L**ISN**G
**2VIS-H2**	WLGVI**WAGGNTN**Y	VI**WAGGNTN**YNSALMS	**WAGGN**	I**WAGGNT**
**2VIS-H3**	R**DFYDYD**V**F**Y**Y**AMDY	**DFYDYD**V**F**Y**Y**AMDY	**DFYDYD**V**F**Y**Y**AMDY	AR**DFYDYD**V**F**Y**Y**AMDY
**2VWE-L1**	Q**D**I**S**N**F**LN	RASQ**D**I**S**N**F**LN	RASQ**D**I**S**N**F**LN	Q**D**I**S**N**F**
**2VWE-L2**	LLI**YY**TS**T**LHS	**Y**TS**T**LHS	**Y**TS**T**LHS	**Y**TS
**2VWE-L3**	QQG**K**TLPP	QQG**K**TLPPT	QQG**K**TLPPT	QQG**K**TLPPT
**2VWE-H1**	YTFTGFWIH	GFWIH	GYTFTGF	GYTFTGFW
**2VWE-H2**	WIGHINPGNGGTNY	HINPGNGGTNYNEKFKR	NPGNGG	INPGNGGT
**2VWE-H3**	RS**YSNYV**RAMDY	S**YSNYV**RAMDY	S**YSNYV**RAMDY	ARS**YSNYV**RAMDY
**2ZCK-L1**	QSVD**FDGD**S**Y**MN	KASQSVD**FDGD**S**Y**MN	KASQSVD**FDGD**S**Y**MN	QSVD**FDGD**S**Y**
**2ZCK-L2**	LLIFAASNLAS	AASNLAS	AASNLAS	AAS
**2ZCK-L3**	QQ**SNE**DPY	QQ**SNE**DPYT	QQ**SNE**DPYT	QQ**SNE**DPYT
**2ZCK-H1**	**YT**F**TTYY**IN	**TYY**IN	G**YT**F**TTY**	G**YT**F**TTYY**
**2ZCK-H2**	WIGR**IAPASGT**T**Y**S	R**IAPASGT**T**Y**SSEMFKD	**APASGT**	**IAPASGT**T
**2ZCK-H3**	RA**DYGFNS**G**E**AMDY	A**DYGFNS**G**E**AMDY	A**DYGFNS**G**E**AMDY	ARA**DYGFNS**G**E**AMDY
**3B2U-L1**	**Q**SVSS**Y**LA	RAS**Q**SVSS**Y**LA	RAS**Q**SVSS**Y**LA	**Q**SVSS**Y**
**3B2U-L2**	LLIY**D**ASNRAT	**D**ASNRAT	**D**ASNRAT	**D**AS
**3B2U-L3**	HQ**YGST**P**L**	HQ**YGST**P**L**T	HQ**YGST**P**L**T	HQ**YGST**P**L**T
**3B2U-H1**	GSIS**SGD**Y**Y**WS	**SGD**Y**Y**WS	GGSIS**SGD**Y	GGSIS**SGD**
**3B2U-H2**	WIG**Y**I**YYS**G**STDY**	**Y**I**YYS**G**STDY**NPSLKS	**YYS**G**S**	I**YYS**G**ST**
**3B2U-H3**	RVS**IFGVG**TFDY	VS**IFGVG**TFDY	VS**IFGVG**TFDY	ARVS**IFGVG**TFDY
**3BKJ-L1**	QSI**VHSN**GHT**Y**LE	RSSQSI**VHSN**GHT**Y**LE	RSSQSI**VHSN**GHT**Y**LE	QSI**VHSN**GHT**Y**
**3BKJ-L2**	LLIYQVSTRFS	QVSTRFS	QVSTRFS	QVS
**3BKJ-L3**	FQ**ASLVPL**	FQ**ASLVPL**T	FQ**ASLVPL**T	FQ**ASLVPL**T
**3BKJ-H1**	FSIRTS**K**VGV**S**	TS**K**VGV**S**	GFSIRTS**K**V	GFSIRTS**K**
**3BKJ-H2**	**W**LA**H**I**YWD**D**D**K**R**Y	**H**I**YWD**D**D**K**R**YNPSLES	**YWD**D**D**	I**YWD**D**D**K
**3BKJ-H3**	R**R**GFYG**RKYEVN**H**F**DY	**R**GFYG**RKYEVN**H**F**DY	**R**GFYG**RKYEVN**H**F**DY	AR**R**GFYG**RKYEVN**H**F**DY
**3BKY-L1**	SSVSYMH	RASSSVSYMH	RASSSVSYMH	SSVSY
**3BKY-L2**	PWIYAPSNLAS	APSNLAS	APSNLAS	APS

**3BKY-L3**	QQ**W**SF**N**P**P**	QQ**W**SF**N**P**P**T	QQ**W**SF**N**P**P**T	QQ**W**SF**N**P**P**T
**3BKY-H1**	YTFTSY**NMH**	SY**NMH**	GYTFTSY	GYTFTSY**N**
**3BKY-H2**	**W**IG**AIY**PG**N**G**DTSY**	**AIY**PG**N**G**DTSY**NQKFKG	**Y**PG**N**G**D**	**IY**PG**N**G**DT**
**3BKY-H3**	R**VVY**YSNSY**WY**FDV	**VVY**YSNSY**WY**FDV	**VVY**YSNSY**WY**FDV	AR**VVY**YSNSY**WY**FDV
**3BN9-L1**	Q**G**I**S**S**Y**LA	RASQ**G**I**S**S**Y**LA	RASQ**G**I**S**S**Y**LA	Q**G**I**S**S**Y**
**3BN9-L2**	LLIYAASSLQS	AASSLQS	AASSLQS	AAS
**3BN9-L3**	QQH**GNL**P**Y**	QQH**GNL**P**Y**T	QQH**GNL**P**Y**T	QQH**GNL**P**Y**T
**3BN9-H1**	**FTFSSYAM**S	**SYAM**S	G**FTFSSY**	G**FTFSSYA**
**3BN9-H2**	WVS**AISGSGGSTY**Y	**AISGSGGSTY**YADSVKG	**SGSGGS**	**ISGSGGST**
**3BN9-H3**	RPYL**TYPQRRGPQNV**SPFDN	PYL**TYPQRRGPQNV**SPFDN	PYL**TYPQRRGPQNV**SPFDN	ARPYL**TYPQRRGPQNV**SPFDN
**3BT2-L1**	SSV**SY**MH	SASSSV**SY**MH	SASSSV**SY**MH	SSV**SY**
**3BT2-L2**	PWIF**E**ISKLAS	**E**ISKLAS	**E**ISKLAS	**E**IS
**3BT2-L3**	QQ**WNYPF**	QQ**WNYPF**T	QQ**WNYPF**T	QQ**WNYPF**T
**3BT2-H1**	YSFTNF**Y**IH	NF**Y**IH	GYSFTNF	GYSFTNF**Y**
**3BT2-H2**	**W**IG**W**I**F**HGS**DNTE**Y	**W**I**F**HGS**DNTE**YNEKFKD	**F**HGS**DN**	I**F**HGS**DNT**
**3BT2-H3**	R**W**GP**HW**YFDV	**W**GP**HW**YFDV	**W**GP**HW**YFDV	AR**W**GP**HW**YFDV
**3C09-L1**	SSVT**Y**M**Y**	SASSSVT**Y**M**Y**	SASSSVT**Y**M**Y**	SSVT**Y**
**3C09-L2**	LLIY**D**TSNLAS	**D**TSNLAS	**D**TSNLAS	**D**TS
**3C09-L3**	QQ**W**S**SHIF**	QQ**W**S**SHIF**T	QQ**W**S**SHIF**T	QQ**W**S**SHIF**T
**3C09-H1**	YTF**TSHW**M**H**	**SHW**M**H**	GYTF**TSH**	GYTF**TSHW**
**3C09-H2**	WIG**E**F**N**P**SN**G**RTN**Y	**E**F**N**P**SN**G**RTN**YNEKFKS	**N**P**SN**G**R**	F**N**P**SN**G**RT**
**3C09-H3**	S**RDYDYAG**R**Y**FDY	**RDYDYAG**R**Y**FDY	**RDYDYAG**R**Y**FDY	AS**RDYDYAG**R**Y**FDY
**3C2A-L1**	SSNIGNN**Y**VL	SGSSSNIGNN**Y**VL	SGSSSNIGNN**Y**VL	SSNIGNN**Y**
**3C2A-L2**	LLIYGNNKRPS	GNNKRPS	GNNKRPS	GNN
**3C2A-L3**	AT**WD**SGL**SADW**	AT**WD**SGL**SADW**V	AT**WD**SGL**SADW**V	AT**WD**SGL**SADW**V
**3C2A-H1**	FTFSDV**W**LN	DV**W**LN	GFTFSDV	GFTFSDV**W**
**3C2A-H2**	WVG**R**I**K**SRT**D**GGTT**D**Y	**R**I**K**SRT**D**GGTT**D**YAASVKG	**K**SRT**D**GGT	I**K**SRT**D**GGTT
**3C2A-H3**	T**DG**FIMIRGV**SEDYYYYY**MDV	**DG**FIMIRGV**SEDYYYYY**MDV	**DG**FIMIRGV**SEDYYYYY**MDV	TT**DG**FIMIRGV**SEDYYYYY**MDV
**3CK0-L1**	QNLL**H**S**IT**RKN**Y**LA	KSSQNLL**H**S**IT**RKN**Y**LA	KSSQNLL**H**S**IT**RKN**Y**LA	QNLL**H**S**IT**RKN**Y**
**3CK0-L2**	LLIYWASTRGS	WASTRGS	WASTRGS	WAS
**3CK0-L3**	**K**Q**SYNLY**	**K**Q**SYNLY**T	**K**Q**SYNLY**T	**K**Q**SYNLY**T
**3CK0-H1**	FTFNTD**A**M**N**	TD**A**M**N**	GFTFNTD	GFTFNTD**A**
**3CK0-H2**	WVA**R**I**R**SKG**F**NFAT**Y**Y	**R**I**R**SKG**F**NFAT**Y**YADSVRD	**R**SKG**F**NFA	I**R**SKG**F**NFAT
**3CK0-H3**	R**GRDGEA**MDY	**GRDGEA**MDY	**GRDGEA**MDY	VR**GRDGEA**MDY
**3CSY-L1**	QSVL**Y**SS**N**N**K**S**Y**LA	KSSQSVL**Y**SS**N**N**K**S**Y**LA	KSSQSVL**Y**SS**N**N**K**S**Y**LA	QSVL**Y**SS**N**N**K**S**Y**
**3CSY-L2**	LLI**YW**AST**RES**	**W**AST**RES**	**W**AST**RES**	**W**AS
**3CSY-L3**	QQYYSAPL	QQYYSAPLT	QQYYSAPLT	QQYYSAPLT
**3CSY-H1**	**T**L**INY**RMN	**NY**RN	GF**T**L**INY**	GF**T**L**INY**R
**3CSY-H2**	WVSSI**SSSSSY**IHY	SI**SSSSSY**IHYADSVKG	**SSSSSY**	I**SSSSSY**I
**3CSY-H3**	VRE**GPRATGYS**M**A**DVF	E**GPRATGYSA**DVFDI	E**GPRATGYSA**DVFDI	VRE**GPRATGYSA**DVFDI
**3CVH-L1**	EDIYNRLA	KASEDIYNRLA	KASEDIYNRLA	EDIYNR
**3CVH-L2**	LLISGATSLET	GATSLET	GATSLET	GAT
**3CVH-L3**	QQY**WSTP**L	QQY**WSTP**LT	QQY**WSTP**LT	QQY**WSTP**LT
**3CVH-H1**	YTFTDY**N**MD	DY**N**MD	GYTFTDY	GYTFTDY**N**
**3CVH-H2**	WIG**D**INPNNGGT**I**Y	**D**INPNNGGT**I**YNQKFKG	NPNNGG	INPNNGGT
**3CVH-H3**	R**K**P**YYGNF**AWFAY	**K**P**YYGNF**AWFAY	**K**P**YYGNF**AWFAY	AR**K**P**YYGNF**AWFAY
**3CXD-L1**	ENIYS**FLA**	RASENIYS**FLA**	RASENIYS**FLA**	ENIYS**F**
**3CXD-L2**	**LLVYA**ATNLAD	**A**ATNLAD	**A**ATNLAD	**A**AT
**3CXD-L3**	**Q**H**FWGT**P**F**	**Q**H**FWGT**P**F**T	**Q**H**FWGT**P**F**T	**Q**H**FWGT**P**F**T

**3CXD-H1**	FTFN**IYA**MN	**IYA**MN	GFTFN**IY**	GFTFN**IYA**
**3CXD-H2**	WVA**RIRSQSN**NYTTYY	**RIRSQSN**NYTTYYADSVKD	**RSQSN**NYT	**IRSQSN**NYTT
**3CXD-H3**	R**QMGD**Y	**QMGD**Y	**QMGD**Y	VR**QMGD**Y
**3D85-L1**	QSISD**Y**L**H**	RASQSISD**Y**L**H**	RASQSISD**YLH**	QSISD**Y**
**3D85-L2**	LLIK**Y**ASQSIS	**Y**ASQSIS	**Y**ASQSIS	**Y**AS
**3D85-L3**	QN**GHSF**P**F**	QN**GHSF**P**F**T	QN**GHSF**P**F**T	QN**GHSF**P**F**T
**3D85-H1**	YTF**TSNV**M**H**	**SNV**M**H**	GYTF**TSN**	GYTF**TSNV**
**3D85-H2**	WIG**Y**I**N**P**YN**DG**TK**Y	**Y**I**N**P**YN**DG**TK**YNEKFKG	**N**P**YN**DG	I**N**P**YN**DG**T**
**3D85-H3**	**RNWDV**AY	**NWDV**AY	**NWDV**AY	A**RNWDV**AY
**3DVG-L1**	QSVSS**A**VA	RASQSVSS**A**VA	RASQSVSS**A**VA	QSVSS**A**
**3DVG-L2**	LLIYSARSLYS	SARSLYS	SARSLYS	SAR
**3DVG-L3**	QQ**Y**SSYS**S**L**F**	QQ**Y**SSYS**S**L**F**T	QQ**Y**SSYS**S**L**F**T	QQ**Y**SSYS**S**L**F**T
**3DVG-H1**	F**N**V**KTGL**I**H**	**TGL**I**H**	GF**N**V**KTG**	GF**N**V**KTGL**
**3DVG-H2**	WVA**Y**I**T**P**YY**GSTSY	**Y**I**T**P**YY**GSTSYADSVKG	**T**P**YY**GS	I**T**P**YY**GST
**3DVG-H3**	R**EYYR**W**YT**AIDY	**EYYR**W**YT**AIDY	**EYYR**W**YT**AIDY	AR**EYYR**W**YT**AIDY
