# Supplementary material for: Structural Consensus among Antibodies Defines the Antigen Binding Site
Source: PLoS Comput Biol. 2012 Feb 23;8(2):e1002388. doi: 10.1371/journal.pcbi.1002388 (PMC3285572; doi:10.1371/journal.pcbi.1002388)
Supplement: Table S6 — Train dataset. Contains the list of PDB structures used to construct Paratome. (PDF) [file pcbi.1002388.s008.pdf]

Table S6. Train dataset. Contains the list of PDB structures used to construct Paratome.

PDB ID	Heavy chain	Light chain	Ag chain
**2HH0**	H	L	P
**1NAK**	H	L	P
**3DGV**	B	A	X
**2B1A**	H	L	P
**1JHL**	H	L	A
**1XGY**	H	L	P
**1CFT**	B	A	C
**1AR1**	C	D	A
**2A6I**	B	A	P
**1SY6**	H	L	A
**1W72**	H	L	A
**2R4S**	H	L	A
**1ZA3**	B	A	S
**2CMR**	H	L	A
**3C09**	H	L	D
**2GDT**	A	B	E
**1U8N**	B	A	C
**2JEL**	H	L	P
**1TZH**	B	A	W
**1H0D**	B	A	C
**2QR0**	B	A	C
**2J88**	H	L	A
**1RJL**	B	A	C
**1TPX**	B	C	A
**2OQJ**	B	A	F
**2FJH**	H	L	V
**1ACY**	H	L	P
**1RZK**	H	L	C
**2QAD**	D	C	A
**1ORS**	B	A	C
**1N8Z**	B	A	C
**1PKQ**	B	A	E
**1E4X**	H	L	P
**2J4W**	H	L	D
**2CXD**	H	L	P

**2QSC**	H	L	P
**3C2A**	H	L	P
**2NZ9**	D	C	A
**2B2X**	H	L	A
**2JIX**	H	L	E
**1P4B**	H	L	P
**2E58**	H	L	P
**2R0L**	H	L	A
**1U95**	B	A	C
**3BKY**	H	L	P
**1QFW**	H	H	A
**1NDG**	B	A	C
**1U8M**	B	A	C
**1HYS**	D	C	A
**2QQL**	H	L	A
**1FSK**	C	B	A
**1MPA**	H	L	P
**2NR6**	D	C	A
**1U8Q**	B	A	C
**1JPS**	H	L	T
**2AP2**	B	A	F
**1TZI**	B	A	V
**1A3R**	H	L	P
**1PZ5**	B	A	C
**1UJ3**	B	A	C
**1DQJ**	B	A	C
**1CZ8**	H	L	W
**1CU4**	H	L	P
**2OZ4**	H	L	A
**1YQV**	H	L	Y
**2B0S**	H	L	P
**2ADF**	H	L	A
**2OR9**	H	L	P
**1ZTX**	H	L	E
**1CFS**	B	A	C
**1AFV**	H	L	A
**1BGX**	H	L	T
**1HI6**	B	A	C
**1NFD**	H	G	C

**3CK0**	H	L	P
**2UZI**	H	L	R
**1EO8**	H	L	A
**2R0W**	H	L	Q
**2FJG**	B	A	W
**1MHP**	H	L	A
**2CK0**	H	L	P
**2VWE**	E	C	A
**2BOC**	A	B	C
**1NSN**	H	L	S
**2I9L**	B	A	I
**1EGJ**	H	L	A
**2GSI**	B	A	W
**1S78**	D	C	A
**1QKZ**	H	L	A
**1XGU**	B	A	C
**1BVK**	B	A	C
**1U92**	B	A	C
**1KC5**	H	L	P
**2NY7**	H	L	G
**1FPT**	H	L	P
**1FBI**	H	L	X
**1GGI**	H	L	P
**1KTR**	H	L	P
**1P2C**	B	A	C
**1VFB**	B	A	C
**1I8K**	B	A	C
**2R0K**	H	L	A
**1JRH**	H	L	I
**1KEN**	H	L	F
**2J5L**	C	B	A
**2HFG**	H	L	R
**2B1H**	H	L	P
**1YNT**	B	A	F
**3B2U**	H	L	A
**1G9N**	H	L	C
**1BOG**	B	A	C
**1E4W**	H	L	P
**1OSP**	H	L	O

**1OAZ**	H	L	A
**2Q8A**	H	L	A
**2OSL**	H	L	P
**1FJ1**	B	A	F
**1KCS**	H	L	P
**1CE1**	H	L	P
**1FE8**	H	L	A
**1AHW**	B	A	F
**1CFN**	B	A	C
**1XIW**	D	C	A
**1ADQ**	H	L	A
**2VDK**	H	L	A
**2B4C**	H	L	C
**1QFU**	H	L	A
**1N6Q**	H	L	A
**1WEJ**	H	L	F
**3BKJ**	H	L	A
**1LKR**	H	L	A
**1ORQ**	B	A	C
**2HRP**	H	L	P
**2IFF**	H	L	Y
**2EH8**	H	L	P
**1Q1J**	H	L	Q
**2QQN**	H	L	A
**1UWX**	H	L	A
**1MLC**	B	A	E
**1FRG**	H	L	P
**2J6E**	H	L	A
**1E6J**	H	L	P
**1N64**	H	L	P
**1KCR**	H	L	P
**2DD8**	H	L	S
**1N0X**	H	L	R
**2BDN**	H	L	A
**3CSY**	A	B	N
**2OTU**	B	A	P
**1U8P**	B	A	C
**1FNS**	H	L	A
**1YJD**	H	L	C

**2AEP**	H	L	A
**1F90**	H	L	E
**2FX7**	H	L	P
**2VIS**	B	A	C
**1IGC**	H	L	A
**2IGF**	H	L	P
**2H1P**	H	L	P
**1NCD**	H	L	N
**1Z3G**	H	L	A
**3BNT**	D	C	B
**1TET**	H	L	P
**2QHR**	H	L	P
**1XCQ**	B	A	P
**1J1P**	H	L	Y
**1YY9**	D	C	A
**1U8O**	B	A	C
**1HIM**	L	H	P
**2ZCK**	L	H	P
**1OTS**	C	D	A
**1KB9**	J	K	F
**1U93**	B	A	C
**2V17**	H	L	A
**1V7M**	H	L	V
**1F58**	H	L	P
**3D85**	B	A	D
**1UAC**	H	L	Y
**1TJI**	H	L	P
**2ARJ**	H	L	Q
**1IQD**	B	A	C
**1XCT**	B	A	P
**3CVH**	H	L	A
**2HKF**	H	L	P
**1SM3**	H	L	P
**1NL0**	H	L	G
**3BT2**	H	L	A
**1NDM**	B	A	C
**1KB5**	H	L	A
**1HH9**	B	A	C
**2H9G**	B	A	R

**1NMA**	H	L	N
**1U8L**	B	A	C
**2BRR**	H	L	P
**2G5B**	B	A	I
**2R56**	H	L	A
**2IPU**	H	L	Q
**1OB1**	B	A	F
**2R29**	H	L	A
**1I9R**	H	L	A
