# Supplementary material for: Structural Consensus among Antibodies Defines the Antigen Binding Site
Source: PLoS Comput Biol. 2012 Feb 23;8(2):e1002388. doi: 10.1371/journal.pcbi.1002388 (PMC3285572; doi:10.1371/journal.pcbi.1002388)
Supplement: Table S7 — Test dataset. Contains the list of PDB structures used to test Paratome. (PDF) [file pcbi.1002388.s009.pdf]

Table S7. Test dataset. Contains the list of PDB structures used to test Paratome.

PDB ID	Heavy chain	Light chain	Ag chain
3LEY	H	L	P
3O6L	H	L	C
3MLU	H	L	P
3LEX	H	L	P
3L5W	H	L	I
3MOD	H	L	P
3MLY	H	L	P
3O6M	H	L	C
3O0R	H	L	C
3O0R	H	L	B
3IVK	H	L	M
2XTJ	D	B	A
3LDB	C	B	A
3A6B	H	L	Y
3MA9	H	L	A
3AB0	B	C	A
3L95	B	A	X
3A67	H	L	Y
3K2U	H	L	A
3NIF	H	L	A
3NH7	H	L	A
3MNW	B	A	P
3MOA	H	L	P
2XQY	G	K	A
3L5X	H	L	A
3LD8	C	B	A
3NID	H	L	A
2WUC	H	L	A
3L5Y	H	L	A
3LHP	H	L	S
3A6C	H	L	Y
3KRS	H	L	D
3KJ6	H	L	A
3MNZ	B	A	P
3LOH	A	B	E
3LOH	C	D	E

**3IXT**	H	L	P
**3LH2**	H	L	S
**3JWD**	H	L	A
**3MAC**	H	L	A
**3N85**	H	L	A
**3MLV**	H	L	P
**3O6R**	A	B	C
**3LIZ**	H	L	A
**3MLX**	H	L	P
**3PNW**	B	A	C
**3MLS**	H	L	P
**3MXW**	H	L	A
**3LEV**	H	L	A
**3OR7**	A	B	C
**3O41**	H	L	P
**3MLZ**	H	L	P
**3KLH**	D	C	B
**2XQB**	H	L	A
**3MLT**	H	L	P
**2XRA**	H	L	A
**3KJ4**	H	L	A
**3LQA**	H	L	C
**3LQA**	H	L	G
**3O30**	H	L	A
**2WUB**	H	L	A
**3NGB**	H	L	G
**3MOB**	H	L	P
**3MLR**	H	L	P
**3NIG**	H	L	A
**3O45**	H	L	P
**3JWO**	H	L	A
**3O2D**	H	L	A
