# Supplementary material for: Structural Consensus among Antibodies Defines the Antigen Binding Site
Source: PLoS Comput Biol. 2012 Feb 23;8(2):e1002388. doi: 10.1371/journal.pcbi.1002388 (PMC3285572; doi:10.1371/journal.pcbi.1002388)
Supplement: Table S8 — The amino acid composition of Paratome-unique Ag binding residues. (PDF) [file pcbi.1002388.s010.pdf]

Table 8S. The amino acid composition of Paratome-unique Ag binding residues.

%	Amino acid	%	Amino acid
1.95	T	35.3	Y
1.3	V	31.4	W
1.3	S	9.8	L
1.3	E	5.25	K
1.3	H	3.95	G
0.65	A	3.25	I
0.65	F	2.6	R
