# Supplementary material for: Structural Consensus among Antibodies Defines the Antigen Binding Site
Source: PLoS Comput Biol. 2012 Feb 23;8(2):e1002388. doi: 10.1371/journal.pcbi.1002388 (PMC3285572; doi:10.1371/journal.pcbi.1002388)
Supplement: Table S9 — The percentage of Paratome-unique residues for each ABR/CDR. (PDF) [file pcbi.1002388.s011.pdf]

To find out from which ABR/CDR Paratome-unique Ag binding residues originate from, we computed the percentage of residues originating from each ABR/CDR (i.e. L1-3, H1-3). To gain additional insight into the differences between the Ag binding residues identified by Paratome and the various CDR identification methods, we computed for each method its own Paratome-unique set of Ag binding regions. For instance, Paratome-Kabat unique is the set of all Ag binding residues identified by Paratome yet not identified by Kabat. Table S9 depicts the results.

The CDRs from which residues that are not identified by the commonly used CDR identification methods originate:

Table 9S. The percentage of Paratome-unique residues for each ABR/CDR**.**

ABR/CDR	Paratome-unique	Paratome-Kabat unique	Paratome-Chothia unique	Paratome-IMGT unique
**L1**	-	-	-	7.4%
**L2**	64.7%	24.3%	11.05%	29.2%
**L3**	-	-	-	-
**H1**	-	48.45%	29.7%	18%
**H2**	35.3%	13.15%	52.95%	45.4%
**H3**	-	14.1%	6.3%	-
